# Selective P450_BM3_ Hydroxylation of the Spiro[3.3]heptane Core as a Route to Potential Drug Fragment Molecules

**DOI:** 10.1021/acs.orglett.5c01265

**Published:** 2025-08-27

**Authors:** Xinxin Zhang, Xiaoning Zhang, Luet L. Wong, Jeremy Robertson

**Affiliations:** † 665886 Oxford Suzhou Centre for Advanced Research, Ruo Shui Road, Suzhou Industrial Park, Jiangsu 215123, P. R. China; ‡ Department of Chemistry, 6396 University of Oxford, Inorganic Chemistry Laboratory, South Parks Road, Oxford OX1 3QR, United Kingdom; § Department of Chemistry, University of Oxford, Chemistry Research Laboratory, Mansfield Road, Oxford OX1 3TA, United Kingdom

## Abstract

Engineered P450_BM3_ enzyme variants, developed from an initial screening panel of 42 enzymes, convert *N-*benzyl spiro[3.3]­heptane-2-carboxamide into three distally monohydroxylated regioisomers with essentially complete enantioselectivity. Two α-hydroxyamide derivatives are also produced. Elaboration of the metabolites by tethered C–H amination leads to spiro[3.3]­heptane motifs substituted with three different functional groups ready for further derivatization.

## Graphical abstract

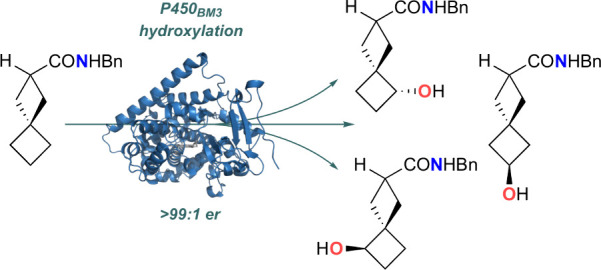

The spiro[3.3]­heptane ring system, first described in 1907,^1^ is gaining in prominence within early stage drug discovery efforts, including as a template for fragment development^2^ and as a bioisosteric replacement for benzene (Figure 1).^3^ This interest has developed sharply over the past decade, no doubt stimulated by widely promoted alternatives to ‘traditional’ medicinal discovery chemistry strategies as exemplified by Lovering’s influential paper correlating the fraction of sp^3^ carbons (Fsp^3^) and number of stereogenic carbons with success in progressing from hit through lead to clinical drug.^4^ Taking benzene as a representative “flatland” template (Fsp^3^ = 0), for which there are only three possible isomers of a given disubstituted derivative, in contrast there are 18 ways to arrange two different substituents around separate rings of the three-dimensional spiro[3.3]­heptane core (Fsp^3^ = 1.0) and all 18 isomers are chiral.^5^ Gaining access to all these isomers would allow a thorough exploration of the relationship between the disposition of functionality around the core and the nature and strength of binding with a given biological target; however, to do so using current methods would be onerous because each isomer would require its own tailored synthesis.

**Figure 1:**
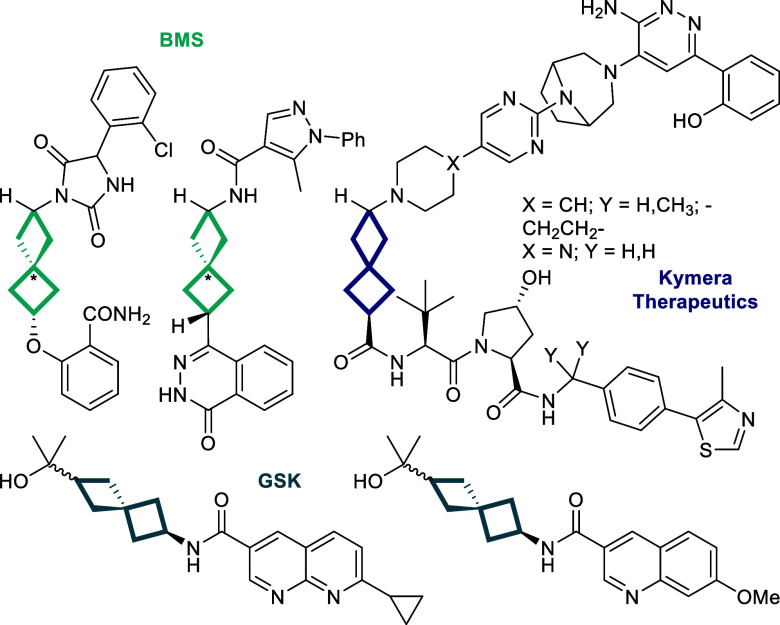
Spiro­[3.3]­bicyclohepta-2,6-diyl linking motifs in ROCK inhibitors (BMS),^10^ anticancer SMARCA degraders (Kymera),^11^ and H-PGDS inhibitors (GSK).^12^

In this communication we illustrate a synthetic route to representative 2,6- and 1,6- disubstituted spiro[3.3]­heptanes, substitution patterns which represent around 90% and 10%, respectively, of the almost 9,000 reported spiro[3.3]­heptanes bearing a single substituent in both rings.^6^ Our strategy of diversifying from a single starting point by P450_BM3_-biocatalytic hydroxylation,^7^ broadly follows the workflow summarized in our recent publications,^7a,8^ here extending the range of cyclobutane-containing hydroxylation substrates to incorporate spirocycles.^9^

In this project, spiro[3.3]­heptane-2-carboxamide derivative **1** (Scheme 1) was selected for screening based on its ease of preparation by sequential Perkin ring synthesis,^13^ lack of chirality to avoid complications arising from the differential reactivity of enantiomers, incorporation of polar functionality to bias productive conformations within the enzyme active site, and inclusion of a UV chromophore to aid analytical detection. From a preliminary screening of 95 P450_BM3_ variants, 42 converted the substrate into hydroxylation products **2**–**6** ^14^ along with several minor, unidentified products (Supporting Information (SI), Table S2.2).

**Scheme 1:**
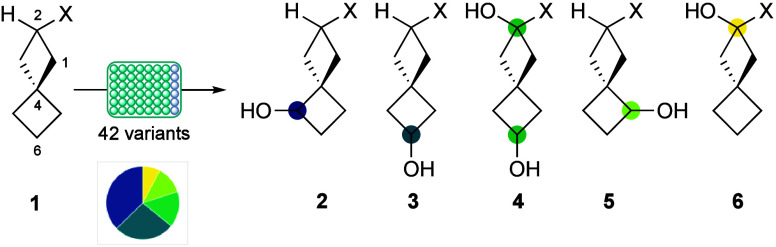
Products from the Initial Screen of Amide **1** ^a^

a X = CONHBn; the products are presented in decreasing overall order of abundance, and the pie chart depicts this as the fraction (GC) of each product summed across all variants.

Taken together, the enzyme variants effected hydroxylation at the *trans-*5 (**2**)- and 6 (**3**, **4**)-positions in roughly equal ratio, these three products comprising about 80% of the total. From this first screening, of the variants producing the most monohydroxylation product **2**, K19/F87V/I263G, rather than GQ/I263G/A330L, was selected for optimization via iterative docking-guided mutagenesis (IDGM)^8^ because of its stability and reliable expression. These docking studies (SI, S7.1–S7.4) suggested that both substrate conversion and selectivity for **2** would be improved by introducing bulkier residues at S72 and L75, smaller residues at F87, and residues at A330 to encourage binding of the benzyl group. Nine single-point variants were screened (SI, Table S2.3a), six of which showed either improved substrate conversion or improved selectivity for **2**. All gave the same major enantiomer, later assigned as (2*S*,4*r*,5*R*)-**2** by Mosher’s ester analysis (SI, S5.1). Of these, the A330F variant delivered complete substrate conversion and the highest ratio of **2** with respect to the next most abundant product (**2**/**3** = 81:19) and so this was used for a second IDGM round focused on improving both regio- and stereoselectivity while retaining conversion. From this, 23 new single- and double-residue variants were generated aiming primarily to block nonproductive docking poses (SI, Table S2.3b); five of these were more regioselective and six were more enantioselective than K19/F87V/I263G. The F87L variant gave 100% conversion, a **2**/**3** ratio of 90:10, and the major product (2*S*,4*r*,5*R*)-**2** in 90% ee; figures for the next best **2**-producing variant (F87I/A330I) were 95% conversion, 84:16 **2**/**3** ratio, and >99% ee.

From the first screening, maximum production of the 6-hydroxylated metabolite **3** was achieved by variant GVQ/I263G and so the IDGM approach was applied from this starting point with single-point variants at four residues (SI, S7.5). Bulkier residues at F87 (V87 in GVQ) and T260 were intended to block nonproductive poses. The F and W variants of A330 were intended to encourage phenyl group binding, with L and I variants employed for comparison purposes. Three F87/A328 double variants were also screened. Primers for four S332 variants became available from a separate project and were also introduced (SI, Table S2.4). Many of the 17 new variants generated (2*R*,4*r*,6*R*)-**3** (assigned by X-ray crystallography, SI, S6.1) with essentially complete enantiopurity, and it was notable that all the variants retaining F87 gave either a lower ee or resulted in production of the opposite enantiomer (up to 70% ee). Five variants showed higher selectivity for **3** relative to **2** although there were no clear correlations in this aspect with residue changes. Of the four variants giving an increased production of this metabolite at higher ee, the A330F variant was the most regioselective (**3**/**2** = 75:25); the F87I variant was marginally less reactive than this but hydroxylation gave both **3** and **2** in >99% ee and with a higher ratio (70:30) than GVQ/I263G itself (**3**/**2** = 65:35; ee 95% and 90%, respectively).

Just three of the 42 variants provided reasonable conversion to the final (*cis-*5-) distally monohydroxylated product (**5**). Of these, variant R19/F87A was chosen for optimization based on its demonstrated high enantioselectivity for this product as well as its relatively clean product profile in which the only minor component observed by GC was the *trans-*5- isomer **2** (**5**/**2** = 76:24). Based on IDGM (SI, S7.6), aiming to disfavor nonproductive poses, single-point replacements of L75 and T260 were made (aromatic F or W; bulkier L or I; SI, Table S2.5). The L75F variant gave slightly improved conversion while maintaining complete enantioselectivity for (2*R*,4*s*,5*R*)-**5** assigned by Mosher’s ester analysis (SI, S5.2) and confirmed by X-ray crystallography (SI, S6.2). Against the IDGM guidance, the T260 variants resulted in poor activity or much lower selectivity for **5**.

Reactions conducted on an ∼1 mmol scale for product characterization afforded moderate isolated yields of the individual monohydroxylated regioisomers **2** (38% from K19/F87I/I263G/A330I), **3** (36% from GVQ/I263G/A330F), and **5** (30% from R19/L75F/F87A). For efficient gram-scale production (≥4.5 mmol) it was necessary to lower the substrate/enzyme ratio, maintain a slightly alkaline reaction pH (7.9), ensure efficient agitation (overhead stirrer) and oxygenation (air bubbling); and include cyclodextrin to aid substrate solubilization. With these parameters established, each isomer was obtained reliably in synthetically useful quantities (Scheme 2) from reaction of ∼1.5–1.6 g of amide **1**.

**Scheme 2:**
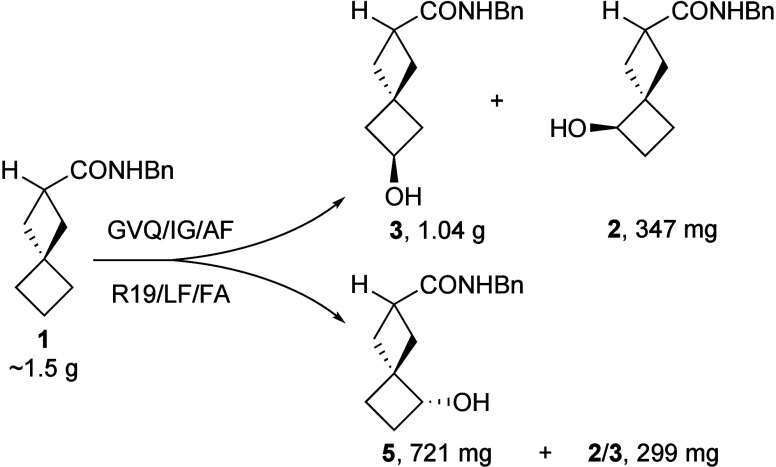
Gram-Scale Preparation of Monohydroxylated Products **2**, **3**, and **5**

The products are able to be elaborated trivially by acylation, or by substitution without rearrangement under conditions which favor an S_N_2 mechanism.^15,16^ The corresponding ketones **7**–**9** were prepared without complication for the production of racemic standards (Scheme 3). Such transformations maintain the 2,5- and 2,6-substitution pattern in the derivatives but this spirocyclic system afforded an opportunity to develop trisubstituted motifs by engaging the newly introduced hydroxyl group to facilitate a second C–H bond functionalization. In earlier work we had discovered that Rh­(II) nitrenoid insertions at nonactivated methylene sites were unacceptably inefficient.^17^ Thermolytic reactions of analogous azidoformates^18,19^ were more successful but side reactions dominate with flexible substrates such as medium ring cyclic amines. The relative rigidity of the spiro[3.3]­heptane framework was, however, expected to promote more predictable reactivity. Thermolysis of azidoformate **10** (Scheme 4) was complete within 3 h at 140 °C to afford ketone **7**, along with the 1,2- and 1,3-C–H amination products **13** and **14**, respectively, in roughly equal proportions. Similarly, azidoformate **11** produced ketone **8** and the 1,2-C–H amination regioisomers **15** and **16** in ∼20:50:30 ratio, respectively. Thermolysis of azidoformate **12** was the least efficient, generating only 1,2-C–H amination product **17** in low yield, with ketone **9** being the major product from this isomer.

**Scheme 3:**
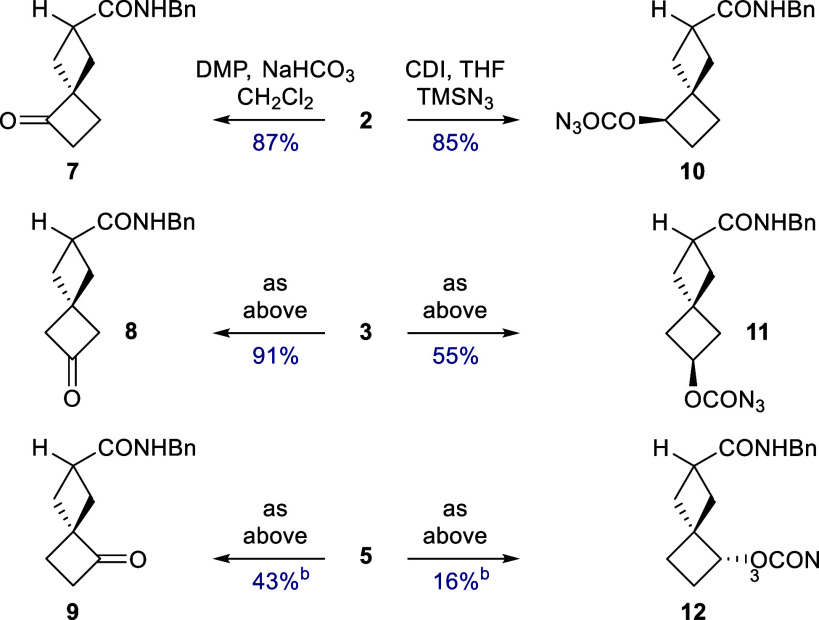
Derivatives of the Monohydroxylation Products^a^

a DMP, Dess–Martin periodinane; CDI, carbonyl diimidazole.
b The yields reflect the relatively low reactivity of the alcohol in **5** which is both neopentylic and *cis-*disposed to the bulky CONHBn group.

**Scheme 4:**
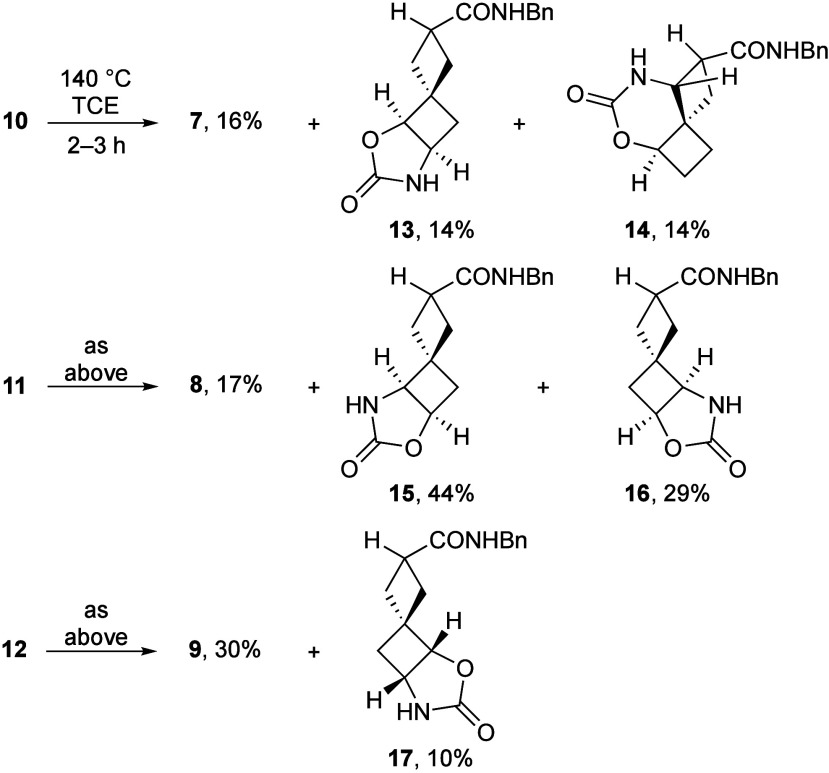
C–H Insertion Products from Thermolysis of Azidoformates **10**–**12**

In conclusion, this work demonstrates that each site of the unfunctionalized cyclobutane ring in amide **1** can be accessed by biocatalytic hydroxylation to produce the *trans* (**2**)- and *cis* (**5**)-5-ol, and 6-ol (**3**) isomers, essentially enantiomerically pure at a synthetically useful scale (>multihundred mg). Combined with a secondary C–H insertion event, made possible by the initial hydroxylation,^20^ trisubstituted spiro[3.3]­heptane derivatives are accessible in just three steps from a common substrate. The so-formed templates bear spatially distinct combinations of carboxyl, hydroxyl, and amino functionality (Figure 2), each of which could be elaborated independently to yield a wide range of derivatives for drug development applications.^21^

**Figure 2:**
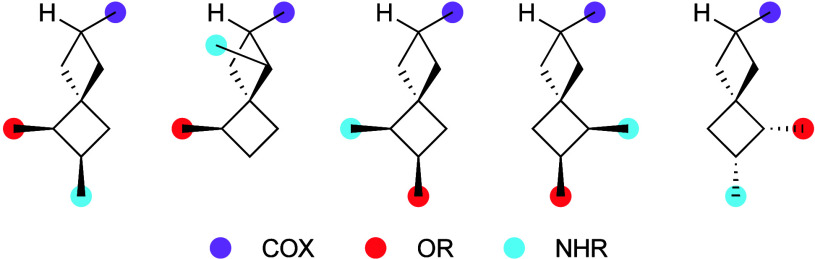
Trisubstituted spiro[3.3]­heptane motifs available by sequential C–H functionalization from amide **1**.

This general approach is equally applicable to other monosubstituted spiro[3.3]­heptane derivatives. For example, in a preliminary screening of *N*-Boc-spiro­[3.3]­heptyl-2-amine (**1**, X = NHBoc), nine variants of the enzyme library (SI, Table S2.1) converted the substrate into three major products in variable ratio: the *trans-*1-, *cis-*5-, and *trans-*5-hydroxylated derivatives.^22^ Validation and optimization of these hydroxylations, and analysis of their absolute stereochemical course will be the subject of further work in this area.

## Supplementary Material



## Data Availability

The data underlying this study are available in the published article and its Supporting Information.

## Notes

Enzyme variants, experimental procedures, characterization data, brief computational details, copies of ^1^H and ^13^C NMR spectra for all compounds, and crystallographic details (PDF)

## References

[1] Fecht H. (1907). Über
Spirocyclane. Ber..

[2] Chernykh A. V., Radchenko D. S., Grygorenko O. O., Daniliuc C. G., Volochnyuk D. M., Komarov I. V. (2015). Synthesis and Structural
Analysis of Angular Monoprotected Diamines Based on Spiro[3.3]­heptane
Scaffold. J. Org. Chem..

[3] Prysiazhniuk K., Datsenko O. P., Polishchuk O., Shulha S., Shablykin O., Nikandrova Y., Horbatok K., Bodenchuk I., Borysko P., Shepilov D., Pishel I., Kubyshkin V., Mykhailiuk P. K. (2024). Spiro­[3.3]­heptane
as a Saturated Benzene Bioisostere. Angew. Chem.,
Int. Ed..

[4] Lovering F., Bikker J., Humblet C. (2009). Escape from Flatland: Increasing
Saturation as an Approach to Improving Clinical Success. J. Med. Chem..

[5] Comprising eight (1X,5Y), four (1X,6Y), four (2X,5Y), and two (2X,6Y) isomers.

[6] From SciFinder: 8390 2,6-disubstituted, 911 1,6-disubstituted, 53 1,5-disubstituted; accessed 20th January 2025.

[7] b Lee, K. W. A. D.Phil. Thesis; University of Oxford, 2023.

[8] Zhang Y., Xiong Z., Li Y., Wilson M., Christensen K. E., Jaques E., Hernández-Lladó P., Robertson J., Wong L. L. (2022). Enantioselective Oxidation of Unactivated
C–H Bonds in Cyclic Amines by Iterative Docking-guided Mutagenesis
of P450_BM3_ (CYP102A1). Nature Synth.

[9] Müller G., Berkenbosch T., Benningshof J. C. J., Stumpfe D., Bajorath J. (2017). Charting Biologically
Relevant Spirocyclic Compound Space. Chem. Eur.
J..

[10] Xie Y., Yue L., Shi Y., Su X., Gan C., Liu H., Xue T., Ye T. (2023). Application
and Study of ROCK Inhibitors in Pulmonary Fibrosis: Recent Developments
and Future Perspectives. J. Med. Chem..

[11] Sabnis R. W. (2022). Novel SMARCA
Degraders for Treating Cancer. ACS Med. Chem.
Lett..

[12] Deaton D. N., Do Y., Holt J. H., Jeune M. R., Kramer H. F., Larkin A. L., Orband-Miller L. A., Peckham G. E., Poole C., Price D. J., Schaller L. T., Shen Y., Shewchuk L. M., Stewart E. L., Stuart J. D., Thomson S. A., Ward P., Wilson J. W., Xu T., Guss J. H., Musetti C., Rendina A. R., Affleck K., Anders D., Hancock A. P., Hobbs H., Hodgson S. T., Hutchinson J., Leveridge M. V., Nicholls H., Smith I. E. D., Somers D. O., Sneddon H. F., Uddin S., Cleasby A., Mortenson P. N., Richardson C., Saxty G. (2019). The Discovery of Quinoline-3-carboxamides
as Hematopoietic Prostaglandin D Synthase (H-PGDS) Inhibitors. Bioorg. Med. Chem..

[13] Opie C. R., Noda H., Shibasaki M., Kumagai N. (2019). All Non-Carbon B_3_NO_2_ Exotic Heterocycles:
Synthesis, Dynamics, and
Catalysis. Chem. Eur. J..

[14] O’Dowd H., Manske J. L., Freedman S. A., Cochran J. E. (2022). Ketoreductase-Catalyzed
Access to Axially Chiral 2,6-Disubstituted Spiro[3.3]­heptane Derivatives. Org. Lett..

[15] Examples of hydroxyl activation and azide displacement are provided in ref 14.

[16] Reactions which promote ionization result in ring contraction. For example, treatment of alcohols **2** or **5** with DAST in DMF resulted in *N-*benzyl 3-cyclopropyl-3-fluorocyclobutane-1-carboxamide; similar reactivity was displayed during tosylation to give the 3-cyclopropyl-3-hydroxy analogue.

[17] Zhang, X. Evolved P450 Mutants as General Oxidation Catalysts for Target Synthesis via Early-/Late-Stage Hydroxylation. DPhil Thesis, University of Oxford, 2022.

[18] Lowe G., Swain S. (1983). Synthesis of 7β-Phenylacetamido-6-oxo-2-oxabicyclo[3.2.0]­heptane-4α-carboxyIic:
acid, a Cyclobutanone Analogue of a β-Lactam Antibiotic. J. Chem. Soc. Chem. Commun..

[19] Yuan P., Plourde R., Shoemaker M. R., Moore C. L., Hansen D. E. (1995). A Mimic
of Both a Torsionally-Distorted
Peptide Ground State and the Transition State for Peptide Bond Hydrolysis:
Synthesis of a Spiro[4.4]­nonyl Derivative. J.
Org. Chem..

[20] He J., Yokoi K., Wixted B., Zhang B., Kawamata Y., Renata H., Baran P. (2024). Biocatalytic
C–H Oxidation
Meets Radical Cross-Coupling: Simplifying Complex Piperidine Synthesis. Science.

[21] Transformations of the carboxamide group also widen access to diverse functional group combinations; for example Hofmann rearrangement^14^ connects to the 2,5- and 2,6-aminoalcohol series, of interest as potential aminophenol isosteres.

[22] Quantities of the mono-hydroxylation products sufficient for spectroscopic characterization were obtained by preparative-scale reactions with RT2/S72G/A330W and RT2/V78A/I263G/A330W.

