# Supplementary material for: Selective P450BM3 Hydroxylation of the Spiro[3.3]heptane Core as a Route to Potential Drug Fragment Molecules
Source: Org Lett. 2025 Aug 27;27(36):9849–53. doi: 10.1021/acs.orglett.5c01265 (PMC12442065; doi:10.1021/acs.orglett.5c01265)

Supporting Information

Selective P450$_{BM3}$ Hydroxylation of the Spiro[3.3]heptane Core as a Route to Potential Drug Fragment Molecules

Xinxin Zhang,[a] Xiaoning Zhang,[a] Luet L. Wong,[a,b] Jeremy Robertson[a,c]*

[a]Oxford Suzhou Centre for Advanced Research, Ruo Shui Road, Suzhou Industrial Park, Jiangsu 215123, P. R. China.
[b]Department of Chemistry, University of Oxford, Inorganic Chemistry Laboratory, South Parks Road, Oxford OX1 3QR, UK.
[c]Department of Chemistry, University of Oxford, Chemistry Research Laboratory, Mansfield Road, Oxford OX1 3TA, UK.

Table of Contents

S1 General methods S3

S1.1 Synthesis S3

S1.2 Enzymes and molecular biology S4

S1.3 Gas chromatographic and supercritical fluid chromatographic analysis S4

S1.4 Molecular dynamics (MD) simulations and substrate docking S5

S1.5 General protocol for analytical screening reactions S5

S2 Enzyme variants and screening data S6

Table S2.1 Screening library S6

Table S2.2 Initial screening data with substrate ***2*** S8

Table S2.3 Screening results for IDGM variants on (a) K19/F87/I263G and (b) K19/F87/I263G/A330F S9

Table S2.4 Screening results for IDGM variants on GVQ/I263G S10

Table S2.5 Screening results for IDGM variants on R19/F87A S10

S3 Chiral SFC traces of isolated metabolites S11

*S3.1 trans-N-Benzyl-5-hydroxyspiro[3.3]heptane-2-carboxamide (**2**)* S11

*S3.2 N-Benzyl-6-hydroxyspiro[3.3]heptane-2-carboxamide (**3**)* S13

*S3.3 cis-N-Benzyl-5-hydroxyspiro[3.3]heptane-2-carboxamide (**5**)* S15

S4 Synthesis procedures S16

*S4.1 N-Benzylspiro[3.3]heptane-2-carboxamide (**1**)* S16

S4.2 General procedure for preparative enzymatic hydroxylations S16

*S4.3 (2S,4r,5R)-N-Benzyl-5-hydroxyspiro[3.3]heptane-2-carboxamide (**2**)* S17

*S4.4 (2R,4r,6R)-N-Benzyl-6-hydroxyspiro[3.3]heptane-2-carboxamide (**3**)* S17

*S4.5 (2S,4s,6S)-N-Benzyl-6-hydroxyspiro[3.3]heptane-2-carboxamide (ent-**3**)* S18

*S4.6 (2R,4s,5R)-N-Benzyl-5-hydroxyspiro[3.3]heptane-2-carboxamide (**5**)* S18

*S4.7 N-benzyl-2,6-dihydroxyspiro[3.3]heptane-2-carboxamide (**4**) and N-Benzyl-2-hydroxyspiro[3.3]heptane-2-carboxamide (**6**)* S18

S4.8 Gram-scale reaction with GVQ/I263G/A330F S19

S4.9	*Gram-scale reaction with R19/F87A/L75F*	S19
S4.10	*General procedure A (preparation of ketones 7–**9**)*	S20
S4.11	*General procedure B (preparation of azidoformates **10–12**)*	S20
S4.12	*General procedure C (preparation of Mosher's esters **S1–S6**)*	S20
S4.13	*trans-N-Benzyl-5-oxospiro[3.3]heptane-2-carboxamide (7)*	S21
S4.14	*N-Benzyl-6-oxospiro[3.3]heptane-2-carboxamide (**8**)*	S21
S4.15	*cis-N-Benzyl-5-oxospiro[3.3]heptane-2-carboxamide (**9**)*	S21
S4.16	*(1R,4r,6S)-6-(Benzylcarbamoyl)spiro[3.3]heptan-1-yl carbonazidate (**10**)*	S22
S4.17	*(2R,4r,6R)-6-(Benzylcarbamoyl)spiro[3.3]heptan-2-yl carbonazidate (**11**)*	S22
S4.18	*(1R,4s,6R)-6-(Benzylcarbamoyl)spiro[3.3]heptan-1-yl carbonazidate (**12**)*	S22
S4.19	*Thermolysis of azidoformate **10***	S23
S4.20	*Thermolysis of azidoformate **11***	S24
S4.21	*Thermolysis of azidoformate **12***	S24
S4.22	*(1R,4r,6S)-6-(Benzylcarbamoyl)spiro[3.3]heptan-1-yl (S)-3,3,3-trifluoro-2-methoxy-2-phenylpropanoate (**S1**)*	S25
S4.23	*(1R,4r,6S)-6-(Benzylcarbamoyl)spiro[3.3]heptan-1-yl (R)-3,3,3-trifluoro-2-methoxy-2-phenylpropanoate (**S2**)*	S25
S4.24	*(2R,4s,6R)-6-(Benzylcarbamoyl)spiro[3.3]heptan-2-yl (S)-3,3,3-trifluoro-2-methoxy-2-phenylpropanoate (**S3**)*	S26
S4.25	*(2R,4s,6R)-6-(Benzylcarbamoyl)spiro[3.3]heptan-2-yl (R)-3,3,3-trifluoro-2-methoxy-2-phenylpropanoate (**S4**)*	S26
S4.26	*(1R,4s,6R)-6-(Benzylcarbamoyl)spiro[3.3]heptan-1-yl (S)-3,3,3-trifluoro-2-methoxy-2-phenylpropanoate (**S5**)*	S27
S4.27	*(1R,4s,6R)-6-(Benzylcarbamoyl)spiro[3.3]heptan-1-yl (R)-3,3,3-trifluoro-2-methoxy-2-phenylpropanoate (**S6**)*	S27
**S5**	**Moshers ester analysis**	S28
S5.1	*Mosher ester analysis for (2S,4r,5R)-N-benzyl-5-hydroxyspiro[3.3]heptane-2-carboxamide **2***	S28
S5.2	*Mosher ester analysis for (2R,4s,5R)-N-benzyl-5-hydroxyspiro[3.3]heptane-2-carboxamide **5***	S28
**S6**	**Crystallographic details**	S29
S6.1	*Crystallographic details for azidoformate **11***	S29
S6.2	*Crystallographic details for azidoformate **12***	S30
**S7**	**Molecular docking analysis and mutation design**	S31
S7.1	*Docking of **1** into the MD-simulated structure of variant K19/F87V/I263G […] for trans alcohol **2***	S31
S7.2	*Docking of **1** into the MD-simulated structure of variant K19/F87V/I263G/A330F […] for trans alcohol **2***	S32
S7.3	*Docking of **1** into the MD-simulated structure of variant K19/F87V/I263G/A330I […] for trans alcohol **2***	S33
S7.4	*Docking of **1** into the MD-simulated structure of variant K19/F87V/I263G/A330F/A82W […] for trans alcohol **2***	S34
S7.5	*Docking of **1** into the MD-simulated structure of variant GVQ/I263G […] for β' alcohol **3***	S35
S7.6	*Docking of **1** into the MD-simulated structure of variant R19/F87A […] for cis alcohol **5***	S36
**S8**	**^{1}H and ^{13}C NMR spectra for all compounds**	S37–S59

S1 General methods

S1.1 Synthesis

All solvents for anhydrous reactions, and commercially-available reagents were used as supplied or purified by standard techniques. Spiro[3.3]heptane-2-carboxylic acid (**1**) was purchased from Bidepharm, China. HPLC grade solvents were purchased from Adamas Reagent Ltd., China. 'Petrol' refers to petroleum ether bp 60–90 °C. All glassware was oven-dried before use and all organic reactions were run under nitrogen unless otherwise stated. Merck aluminum-backed TLC silica gel 60 F_{254} plates (0.2 mm) were used for TLC analysis and were visualized with ultra-violet light before staining with $KMnO_4$, phosphomolybdic acid or vanillin solutions, developing with heat. General-Reagent silica gel (60 Å, 230–400 mesh, 40–63 µm) or Biotage Selekt automated flash purification systems were used for column chromatography, and the solvent system used and retention factors are recorded.

Proton (^1H) and carbon (^{13}C) NMR spectra were recorded on a Jeol ECZ 400 MHz spectrometer. Chemical shift (δ_H and δ_C) values are recorded to the nearest 0.01 ppm and 0.1 ppm, respectively; coupling constants have been rounded to the nearest 0.5 Hz. Peak multiplicities are described as broad (br.), singlet (s), doublet (d), triplet (t), quartet (q), multiplet (m) etc. NMR spectra are referenced (in MestReNova) to the appropriate solvent resonance: $CDCl_3$ 7.26/77.16 ppm; acetone-d_6 2.05/206.26 ppm. Peak assignments were made based on chemical shift, integration, coupling constants, COSY and HSQC spectra. HMBC spectra were obtained in selected cases as necessary. Infra-red (IR) spectra were recorded using a SHIMADZU (IRAffinity-1S) FT-IR spectrometer. Absorption maxima are reported in wavenumbers/cm^{-1} and described as strong (s), medium (m), weak (w) and broad (br.) relative to the most intense peak; weak peaks not attributed to a specific functional group are not reported. High resolution mass spectra (HRMS) were obtained for novel compounds on a Thermo Scientific Orbitrap Elite by ESI or Atmospheric Pressure Chemical Ionisation (APCI); mass to charge ratios (*m/z*) are reported in Daltons. Melting points were recorded in degrees Celsius (°C) using a Hanon MP450 automatic melting point apparatus. Polarimetry data were acquired on an Anton Paar MCP 150 polarimeter; measurements were taken at 25 °C using a wavelength of 589.44 nm; the pathlength of the cell was 1 dm. Specific rotations, $[\alpha]^{25}_D$ were calculated by the formula $[\alpha]^{25}_D = a/(l \times c)$, where a is the average of three readings taken from the polarimeter, l is the pathlength in dm, and c is the sample concentration in g/mL.

S1.2 Enzymes and molecular biology

Bacterial culture media components were supplied by Adamas-beta, Sinopharm, and General-Reagent, China; Kanamycin, IPTG and Lysozyme by Macklin Biochemicals, China; NADP+ by Bidepharm, China; and glucose dehydrogenase (GDH) by Angel Yeast, China. Oligonucleotides for site directed mutagenesis were supplied by Genewiz, China. The standard phosphate buffer used as reaction media in biotransformations was prepared from KH_2PO_4 (2.40 g) and K_2HPO_4 (31.7 g) made up to 1.0 L in deionized water, and the pH was adjusted to 7.9 with 1.0 M aq. KH_2PO_4 solution before use.

Genes encoding $P450_{BM3}$ enzymes were cloned in the pET28+ vector by NcoI and BamHI restriction sites. Site-directed mutagenesis was carried out by standard PCR-based protocols using a KOD Hot Start DNA Polymerase kit from Sigma-Aldrich, UK. The presence of the target mutation(s) was confirmed by DNA sequencing. The relevant plasmid was transformed into chemically competent *E. coli* BL21 (DE3) cells for enzyme production and subsequent purification as described in the following section. P450 content was quantified using the CO-difference method, using UV-vis spectra acquired on a SHIMAZU UV-1900 UV-VS spectrophotometer at 30 °C using 1 cm pathlength quartz cuvettes.

S1.3 Gas chromatographic and supercritical fluid chromatographic analysis

Gas chromatographic (GC) analyses were carried out with a Shimadzu *Nexis* GC-2030 instrument equipped with a flame ionisation detector (FID) and an AOC-20i Plus autosampler using a DB-1fused silica column (30 m × 0.25 mm i.d. × 0.25 μm film thickness) from Agilent Technologies using helium as the carrier gas at a flow rate of 1.5 mL/min. For normal phase analyses, both the injector and the FID were held at 250 °C.

For initial screening analysis of the metabolites, the oven temperature was held at 180 °C for 0.5 min then raised at 20 °C/min to 280 °C and held for another 1 min (**Ramp 1**). Retention times are: *N*-benzyl-2-hydroxyspiro[3.3]heptane-2-carboxamide (**6**), 4.15 min; *cis*-*N*-benzyl-5-hydroxyspiro[3.3]heptane-2-carboxamide (**5**), 4.86 min; *N*-benzyl-6-hydroxyspiro[3.3]heptane-2-carboxamide (**3**) and *trans*-*N*-benzyl-5-hydroxyspiro[3.3]heptane-2-carboxamide (**2**), 4.98 min; *N*-benzyl-2,6-dihydroxyspiro[3.3]heptane-2-carboxamide (**4**), 5.11 min.

For second/third generation library screening analysis of the metabolites, the oven temperature was held at 180 °C for 0.5 min then raised at 3 °C/min to 215 °C (**Ramp 2**). Retention time for compounds are: *cis*-*N*-benzyl-5-hydroxyspiro[3.3]heptane-2-carboxamide (**5**), 10.86 min; *N*-benzyl-6-hydroxyspiro[3.3]heptane-2-carboxamide (**3**), 11.22 min; *trans*-*N*-benzyl-5-hydroxyspiro[3.3]heptane-2-carboxamide (**2**), 11.31 min.

Supercritical fluid chromatographic (SFC) analyses of enantiomeric ratio were carried out on a Shimadzu Nexera UC system, Japan, and monitored using a photodiode array detector using chiral Trefoil CEL1 or CEL2 columns (3.0 mm×150 mm×2.5 µm) from Waters Co., USA and MeOH/liquid CO_2 as mobile phase.

For chiral SFC analysis of the enantiomeric excess of product *trans*-*N*-benzyl-5-hydroxyspiro[3.3]heptane-2-carboxamide (**2**), using chiral column CEL1, the mobile phase was set at 10% MeOH/liquid CO_2 with a flow of 1.5 mL/min (**M1**). Retention times for the pair of enantiomers are (2*S*,4*r*,5*R*)-**2**, 3.27 min and (2*R*,4*r*,5*S*)-**2**, 3.55 min.

For chiral SFC analysis of the enantiomeric excess of product *N*-benzyl-6-hydroxyspiro[3.3]heptane-2-carboxamide (**3**), using chiral column CEL1, the mobile phase was set at 10% MeOH/liquid CO_2 with a flow rate of 1.5 mL/min (**M1**). Retention times for the pair of enantiomers are (2*R*,4*r*,6*R*)-**3**, 4.69 min and (2*S*,4*s*,6*S*)-**3**, 5.22 min.

For chiral SFC analysis of the enantiomeric excess of product *cis*-*N*-benzyl-5-hydroxyspiro[3.3]heptane-2-carboxamide (**5**), using chiral column CEL2, the mobile phase was set at 15% MeOH/liquid CO_2 with a flow of 0.5 mL/min (**M2**). Retention times for the pair of enantiomers are (2*R*,4*s*,5*R*)-**5**, 7.11 min and (2*S*,4*s*,5*S*)-**5**, 7.40 min.

S1.4 Molecular dynamics (MD) simulations and substrate docking

Full details are provided in Zhang *et al.*, *Nature Synth.* **2022**, *1*, 936–945.

S1.5 General protocol for analytical screening reactions

Library screening reactions were carried out in a volume of 1.0 mL in phosphate buffer (200 mM, pH 7.9) in 24-well plates. To each vial, substrate was added as stock solution in ethanol (10 µL, 200 mM) to a final concentration of 2 mM and $P450_{BM3}$ variants as stock solution (100 µL, 10 µM) to a working concentration of 1 µM, giving a 2000:1 ratio of substrate to enzyme. Solutions of GDH (10 µL, 4 U/mL) and glucose (100 µL, 100 mM) were added to regenerate the NADPH cofactor. Reactions were initiated by the addition of a solution of NADP+ (5 µL, 40 µM). The 24-well plates were shaken at 120 rpm for 14 h at 25 °C. Each reaction was then extracted with ethyl acetate (200 µL). After centrifugation at 14300 g, the organic phase was analysed by GC. Conversions <1% are denoted as ‘0’.

S2 Enzyme variants and screening data

Table S2.1 Screening library

Variant	**R** 47	**Y** 51	**S** 72	**A** 74	**V** 78	**F** 87	**H** 171	**A** 184	**L** 188	**A** 191	**N** 239	**I** 259	**I** 263	**A** 264	**E** 267	**A** 276	**Q** 307	**N** 319	**A** 328	**A** 330	**L** 353	**I** 401
**GGQ**/IG				**G**		**G**			**Q**				G									
**GIQ**/IG/AL				**G**		**I**			**Q**				G			L						
**GQ**/IG				**G**					**Q**				G									
**GQ**/IG/AF				**G**					**Q**				G			F						
**GQ**/IG/AI				**G**					**Q**				G			I						
**GQ**/IG/AL				**G**					**Q**				G			L						
**GV**/A184I/A264G/A328G				**G**		**V**		I						G					G			
**GV**/A184I/A328G				**G**		**V**		I											G			
**GV**/A184I/I263G/A328G				**G**		**V**		I					G						G			
**GVQ**				**G**		**V**			**Q**													
**GVQ**/A264G				**G**		**V**			**Q**					G								
**GVQ**/A330W				**G**		**V**			**Q**											W		
**GVQ**/I263G				**G**		**V**			**Q**				G									
**GVQ**/I263G/A264G				**G**		**V**			**Q**				G	G								
I263G													G									
**K19**/A264G							**L**							G			**H**	**Y**				
**K19**/A328G							**L**										**H**	**Y**	G			
**K19**/F87A						A	**L**										**H**	**Y**				
**K19**/F87A/I263A						A	**L**						A				**H**	**Y**				
**K19**/F87A/I263A/A330W						A	**L**						A				**H**	**Y**		W		
**K19**/F87A/I263G/						A	**L**						G				**H**	**Y**				
**K19**/F87L						L	**L**										**H**	**Y**				
**K19**/F87L/I263G/						L	**L**						G				**H**	**Y**				
**K19**/F87V/A264G						V	**L**							G			**H**	**Y**				
**K19**/F87V/I263G						V	**L**						G				**H**	**Y**				
**KT2**										**T**	**H**	**V**				**T**					**I**	
**R19**	**L**	**F**					**L**										**H**	**Y**				

**R19**/F87A	**L**	**F**				A	**L**										**H**	**Y**				
**R19**/F87A/A328L	**L**	**F**				A	**L**										**H**	**Y**	L			
**R19**/F87I	**L**	**F**				I	**L**										**H**	**Y**				
**RLYF**/H171L/I263G	**L**	**F**					L						**G**									
**RP**/V78I/E267V	**L**	**F**			I										V							**P**
**RT2**	**L**	**F**								**T**	**H**	**V**				**T**					**I**	
**RT2**/A330H	**L**	**F**								**T**	**H**	**V**				**T**				H	**I**	
**RT2**/A330P	**L**	**F**								**T**	**H**	**V**				**T**				P	**I**	
**RT2**/A330W	**L**	**F**								**T**	**H**	**V**				**T**				W	**I**	
**RT2**/I263G/A330W	**L**	**F**								**T**	**H**	**V**	G			**T**				W	**I**	
**RT2**/S72G/A330W	**L**	**F**	G							**T**	**H**	**V**				**T**				W	**I**	
**RT2**/S72L/A330W	**L**	**F**	L							**T**	**H**	**V**				**T**				W	**I**	
**RT2**/S72L/I263G/A330W	**L**	**F**								**T**	**H**	**V**				**T**					**I**	
**RT2**/V78A/I263G/A330W	**L**	**F**			A					**T**	**H**	**V**	G			**T**				W	**I**	
WT																						

Table S2.2 Initial screening data with substrate **2** (2000:1 substrate-to-enzyme ratio), ordered by conversion (where 1 = 100%); the three variants taken forward for IDGM are shown in bold; the top three metabolites in each case (≥10% abundance) are highlighted in green, yellow, and pink, respectively.

Variant	Conv.	2	3	4	5	6
GVQ/A330W	0.83	0.05	0.28	0	0.51	0.16
GQ/IG/AL	0.8	1	0	0	0	0
**K19/F87V/I263G**	0.75	0.75	0.25	0	0	0
GV/A184I/A328G	0.73	0.04	0.26	0.17	0	0.53
K19/F87A	0.67	0.1	0.2	0.04	0.63	0.03
K19/F87A/I263G	0.67	0.83	0.1	0.02	0.05	0
**GVQ/I263G**	0.64	0.35	0.65	0	0	0
RP/V78I/E267V	0.61	0.02	0.18	0.69	0	0.11
GVQ/I263G/A264G	0.44	0.33	0.63	0.04	0	0
R19	0.44	0	0.24	0.51	0.25	0
RLYF/H171L/I263G	0.43	0.95	0	0.03	0.01	0.01
K19/F87L/I263G	0.42	0.74	0.22	0.04	0	0
K19/F87V/A264G	0.42	0.03	0.22	0.63	0	0.12
RT2/S72G/A330W	0.42	0.08	0.3	0.39	0.03	0.2
K19/F87A/I263A	0.4	0.67	0.21	0.1	0	0.02
GV/A184I/A264G/A328G	0.39	0.04	0.31	0.15	0	0.5
RT2/A330H	0.38	0.13	0.29	0.46	0.05	0.07
**R19/F87A**	0.37	0.24	0	0	0.76	0
GV/A184I/I263G/A328G	0.35	0.24	0.74	0	0	0.02
RT2	0.33	0	0.34	0.42	0.24	0
GGQ/IG	0.32	0.66	0.3	0.04	0	0
K19/F87A/I263A/A330W	0.3	0.42	0.46	0	0	0.12
GVQ/A264G	0.29	0.04	0.42	0.5	0	0.04
KT2	0.29	0.03	0.38	0.35	0.21	0.03
GQ/IG/AF	0.23	0.79	0.21	0	0	0
GIQ/IG/AL	0.2	1	0	0	0	0
K19/A328G	0.17	0.82	0	0	0.18	0
RT2/A330W	0.14	0.74	0.26	0	0	0
WT	0.14	0.24	0	0.33	0.43	0
GVQ	0.13	0.05	0.34	0.03	0.53	0.05
K19/F87L	0.13	0.06	0.32	0.51	0.11	0
GQ/IG/AI	0.12	0.86	0.14	0	0	0
RT2/S72L/A330W	0.12	0.07	0.63	0.17	0	0.13
RT2/V78A/I263G/A330W	0.09	0	1	0	0	0
GQ/IG	0.08	0.69	0.31	0	0	0
K19/A264G	0.07	0	0.14	0.46	0.4	0
R19/F87I	0.07	1	0	0	0	0
R19/F87A/A328L	0.06	1	0	0	0	0
RT2/A330P	0.04	0	1	0	0	0
RT2/S72L/I263G/A330W	0.04	0.13	0.39	0.48	0	0
RT2/I263G/A330W	0.03	0.7	0.3	0	0	0
I263G	0.01	0	1	0	0	0
**SUMs**		**15.89**	**13.02**	**6.56**	**4.39**	**2.14**

Table S2.3 Screening results for IDGM variants on (a) K19/F87/I263G and (b) K19/F87/I263G/A330F (2000:1 substrate-to-enzyme ratio).

(a) Base enzyme (green)	Variant	Conv.	2	2-ee/%	3	3-ee/%	5	Other	2:3 ratio
K19/F87V/I263G	A330F	1	0.81	74	0.19	>99	0	0	4.3
K19/F87V/I263G	S72W	1	0.5	84	0.5	>99	0.03	0	1.0
K19/F87V/I263G	F87I	0.98	0.67	>99	0.3	>99	0.04	0	2.2
K19/F87V/I263G	A330I	0.96	0.72	84	0.25	>99	0.03	0	2.9
K19/F87V/I263G	A330L	0.95	0.55	84	0.43	>99	0.02	0	1.3
K19/F87V/I263G	—	0.75	0.75	84	0.25	>99	0	0	3.0
K19/F87V/I263G	A330W	0.69	0.79	84	0.21	>99	0.02	0	3.8
K19/F87V/I263G	S72F	0.68	0.62	90	0.32	>99	0	0.06	1.9
K19/F87V/I263G	F87L	0.53	0.37	86	0.63	>99	0	0	0.6
K19/F87V/I263G	L75W	0.4	0.06	>99	0.94	>99	0	0	0.1

(b) Base enzyme (green)	Variant	Conv.	2	2-ee/%	3	3-ee/%	5	Other	2:3 ratio
K19/F87V/I263G/A330F	F87L	1	0.9	90	0.1	>99	0	0	9.0
K19/F87V/I263G/A330F	F87I	1	0.83	72	0.17	>99	0	0.18	4.9
K19/F87V/I263G/A330F	—	1	0.81	74	0.19	>99	0	0	4.3
K19/F87V/I263G/A330F	L181M	1	0.26	94	0.2	>99	0.54	0	1.3
K19/F87V/I263G/A330F	L437F	0.98	0.6	62	0.4	>99	0	0	1.5
K19/F87V/I263G/A330F	L181W	0.98	0.57	61	0.43	>99	0	0	1.3
K19/F87V/I263G/A330F	F87I/A330I	0.95	0.84	>99	0.16	>99	0	0	5.3
K19/F87V/I263G/A330F	S72W	0.9	0.8	84	0.18	>99	0	0	4.4
K19/F87V/I263G/A330F	A82I	0.9	0.55	24	0.44	50	0	0	1.3
K19/F87V/I263G/A330F	A82Y/A330I	0.9	0.36	70	0.64	40	0	0	0.6
K19/F87V/I263G/A330F	A82L/A330I	0.85	0.55	40	0.45	44	0	0	1.2
K19/F87V/I263G/A330F	A82L	0.74	0.19	64	0.4	70	0	0.41	0.5
K19/F87V/I263G/A330F	A82F/A330I	0.72	0.61	74	0.37	24	0	0	1.6
K19/F87V/I263G/A330F	A82Y	0.64	0.81	66	0.19	72	0	0	4.3
K19/F87V/I263G/A330F	S72F	0.53	0.27	>99	0.45	>99	0	0.29	0.6
K19/F87V/I263G/A330F	A82W/A330I	0.52	0.04	–8	0.32	–22	0	0.16	0.1
K19/F87V/I263G/A330F	A82F	0.45	0.82	70	0.18	74	0	0	4.6
K19/F87V/I263G/A330F	A82W	0.29	0.6	>99	0.29	>99	0	0.11	2.1
K19/F87V/I263G/A330F	A82H	0.14	0.57	48	0.43	56	0	0	1.3
K19/F87V/I263G/A330F	A82H/A330I	0.12	0.47	60	0.53	40	0	0	0.9
K19/F87V/I263G/A330F	A82C	0							
K19/F87V/I263G/A330F	A82R	0							
K19/F87V/I263G/A330F	L437W	0							
K19/F87V/I263G/A330F	L437M	0							

Table S2.4 Screening results for IDGM variants on GVQ/I263G (2000:1 substrate-to-enzyme ratio).

Base enzyme (green)	Variant	Conv.	2	2-ee/%	3	3-ee/%	5	3:2 ratio
GVQ/I263G	A330F	1	0.25	78	0.75	>99	0	3
GVQ/I263G	F87F/A328F	0.95	0.5	60	0.5	38	0	1
GVQ/I263G	F87I	0.82	0.3	>99	0.7	>99	0	2.3
GVQ/I263G	A330W	0.66	0.29	94	0.71	>99	0	2.4
GVQ/I263G		0.61	0.35	90	0.65	95	0	1.9
GVQ/I263G	A330I	0.6	0.36	>99	0.64	>99	0	1.8
GVQ/I263G	A330L	0.59	0.36	>99	0.64	>99	0	1.8
GVQ/I263G	S332W	0.58	0.36	90	0.63	>99	0	1.8
GVQ/I263G	S332F	0.58	0.35	92	0.65	>99	0	1.9
GVQ/I263G	F87L	0.51	0.3	>99	0.7	>99	0	2.3
GVQ/I263G	F87F/A328L	0.48	0.6	52	0.34	–60	0.07	0.6
GVQ/I263G	S332L	0.43	0.37	>99	0.63	>99	0	1.7
GVQ/I263G	S332I	0.4	0.46	>99	0.54	–70	0	1.2
GVQ/1263G	A184I	0.31	0.3	>99	0.7	>99	0	2.3
GIQ/I263G	F87I/A328L	0.29	0.64	>99	0.2	>99	0.16	0.3
GVQ/I263G	F87F	0.12	0.37	26	0.43	–68	0.2	1.2
GVQ/I263G	T260I	0.09	0.6	>99	0.4	>99	0	0.7
GVQ/I263G	T260F	0.03	0.38	>99	0.62	>99	0	1.6

Table S2.5 Screening results for IDGM variants on R19/F87A (2000:1 substrate-to-enzyme ratio).

Base enzyme (green)	Variant	Conv.	5	5-ee/%	2 + 3	Other
R19/F87A	L75F	0.91	0.68	>99	0.31	0.01
R19/F87A		0.88	0.65		0.25	0.1
R19/F87A	T260L	0.64	0.03		0.97	0
R19/F87A	T260I	0.17	0		1	0
R19/F87A	L75W	0.02	1		0	0
R19/F87A	T260W	0				
R19/F87A	T260F	0				

S3 Chiral SFC traces of isolated metabolites

S3.1 trans-N-Benzyl-5-hydroxyspiro[3.3]heptane-2-carboxamide (**2**)

Racemate

Trefoil CEL1 column (3.0 mm ×150 mm × 2.5 µm); mobile phase: 10% MeOH/liquid CO_2, flow: 1.5 mL/min.

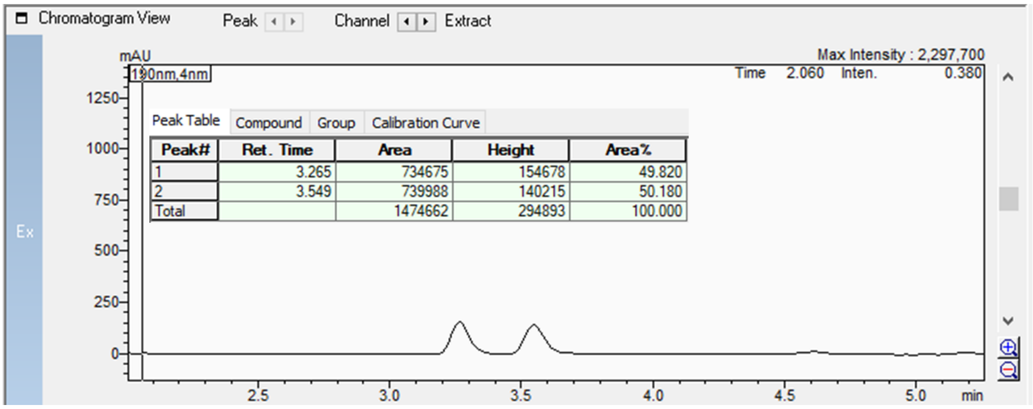

Peak#	Ret. Time	Area	Height	Area%
1	3.265	734675	154678	49.820
2	3.549	739988	140215	50.180
Total		1474662	294893	100.000

(2S,4r,5R)-N-Benzyl-5-hydroxyspiro[3.3]heptane-2-carboxamide

(from GVQ/I263G/A330W)

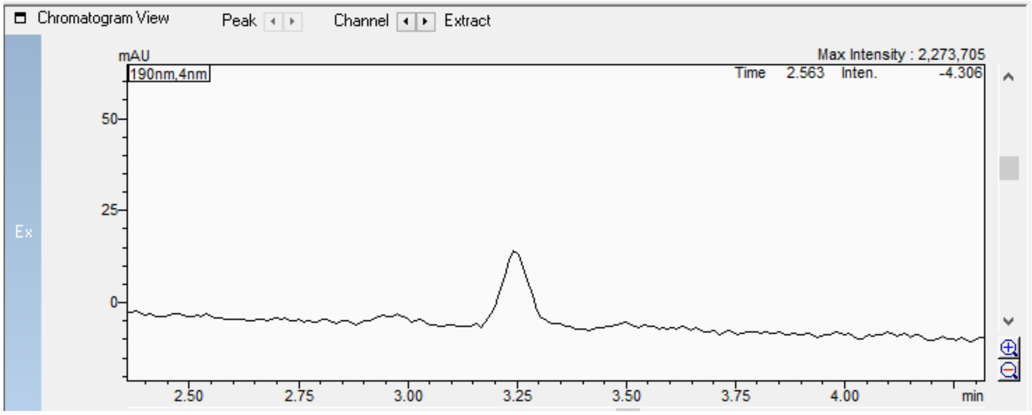

(from K19/F87I/I263G/A330I)

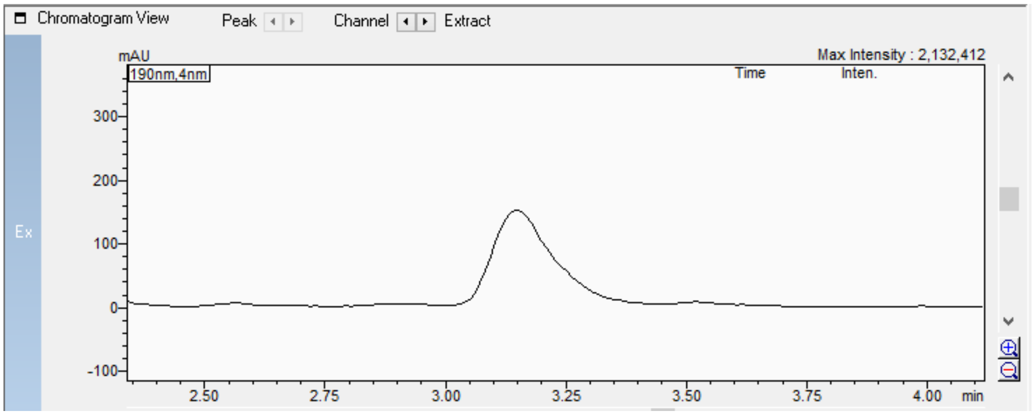

(from GVQ/I263G/A330F) – 78% *ee*

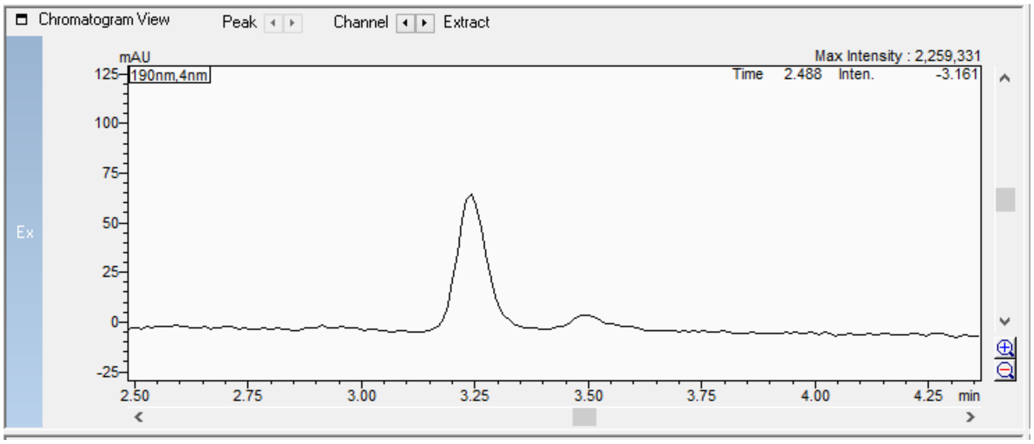

S3.2 N-Benzyl-6-hydroxyspiro[3.3]heptane-2-carboxamide (**3**)

Racemate

Trefoil CEL1 column (3.0 mm ×150 mm × 2.5 μm); mobile phase: 10% MeOH/liquid CO_2, flow: 1.5 mL/min.

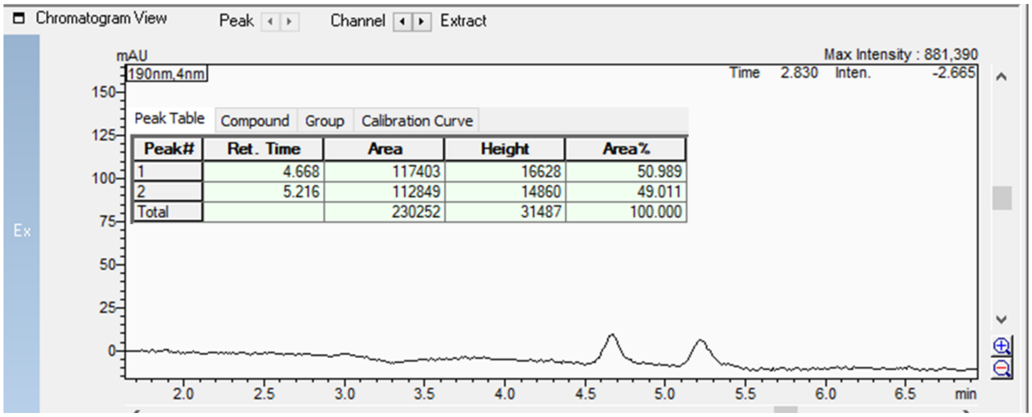

(2R,4r,6R)-N-Benzyl-6-hydroxyspiro[3.3]heptane-2-carboxamide

(from GVQ/I263G/A330F)

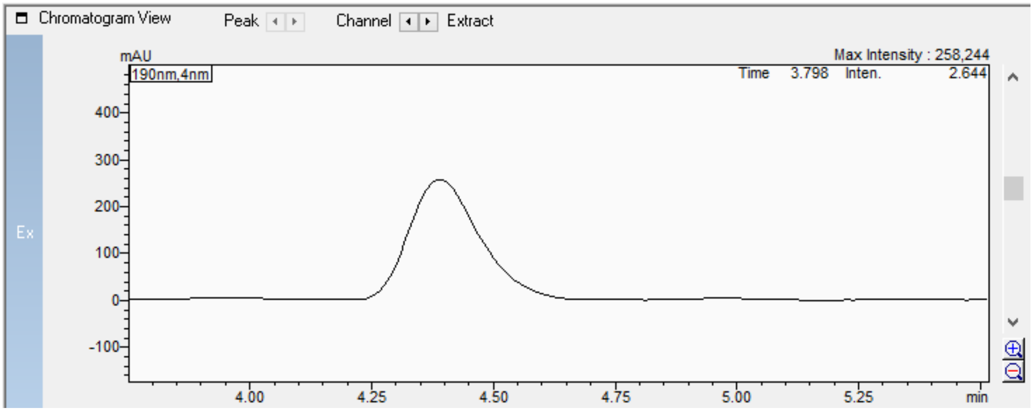

(from K19/F87V/I263G)

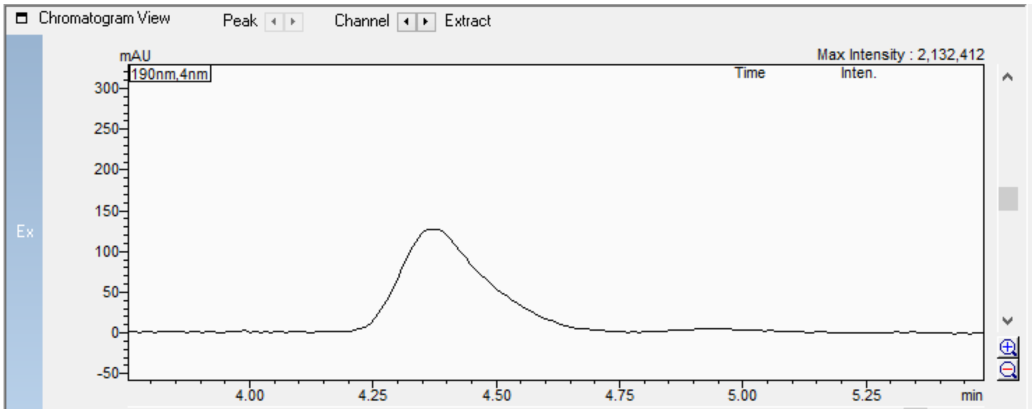

(from K19/F87I/I263G/A330I)

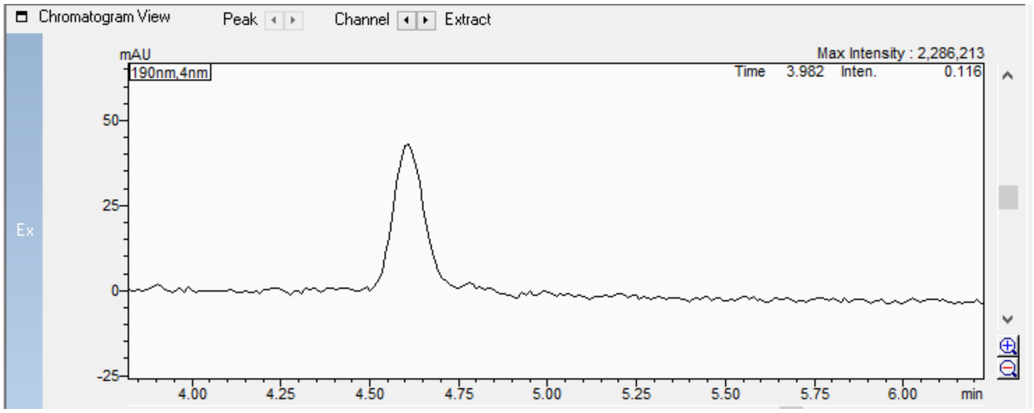

(2S,4s,6S)-N-Benzyl-6-hydroxyspiro[3.3]heptane-2-carboxamide

(from R19)

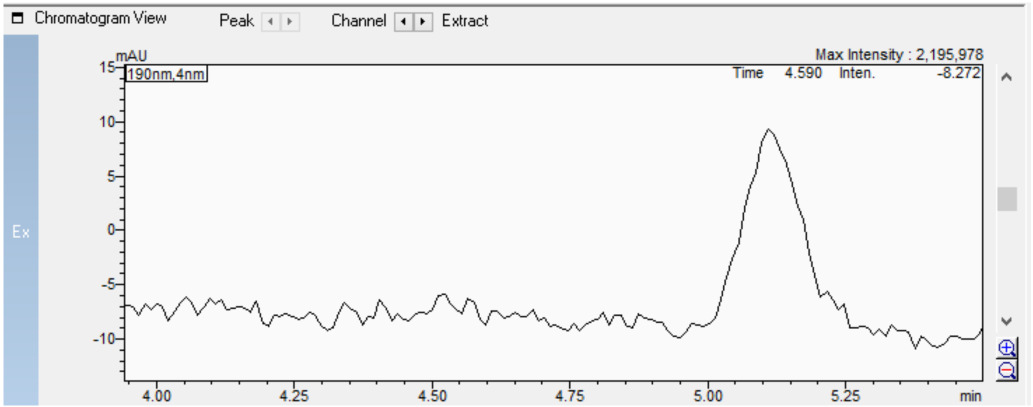

S3.3 cis-N-Benzyl-5-hydroxyspiro[3.3]heptane-2-carboxamide (**5**)

Racemate

Trefoil CEL2 column (3.0 mm ×150 mm × 2.5 µm); mobile phase: 15% MeOH/liquid CO_2, flow: 0.5 mL/min.

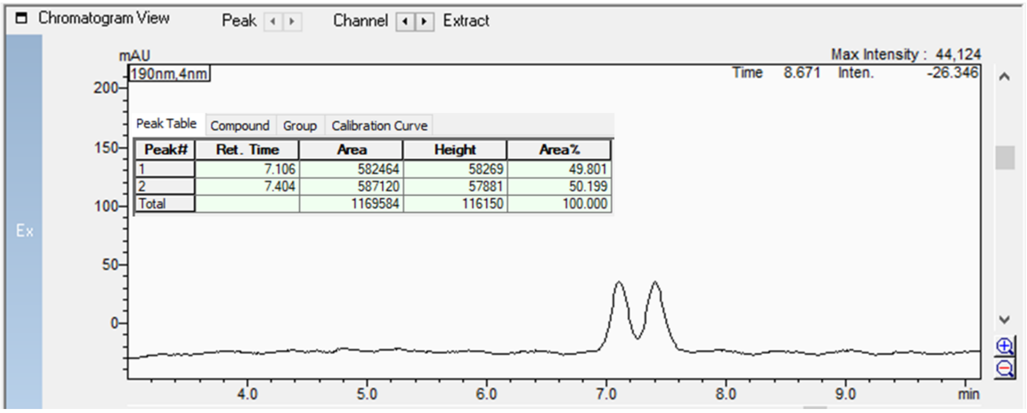

Peak#	Ret. Time	Area	Height	Area%
1	7.106	582464	58269	49.801
2	7.404	587120	57881	50.199
Total		1169584	116150	100.000

(2R,4s,5R)-N-Benzyl-5-hydroxyspiro[3.3]heptane-2-carboxamide

(from R19/F87A/L75F)

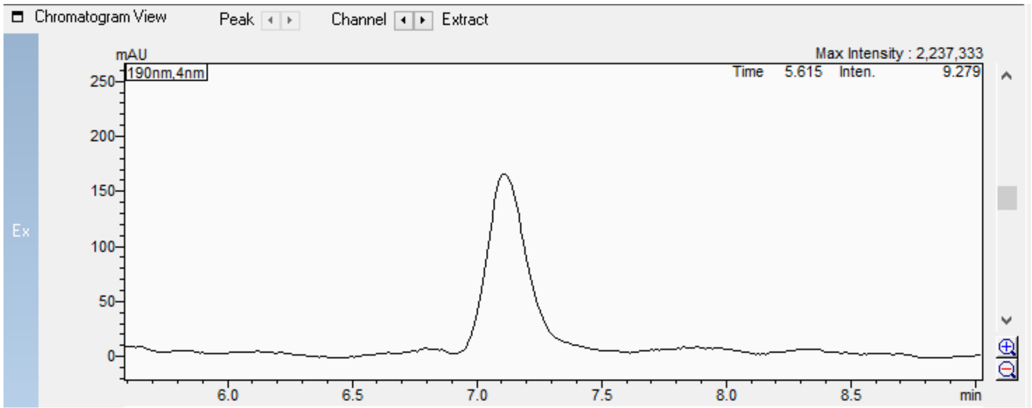

S4 Synthesis procedures

*S4.1 N-Benzylspiro[3.3]heptane-2-carboxamide (**1**)*[1]

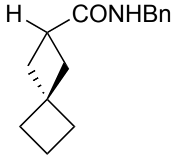

To a solution of spiro[3.3]heptane-2-carboxylic acid (2.80 g, 20.0 mmol) in dichloromethane (400 mL) at room temperature was added, sequentially, benzylamine (2.61 mL, 23.9 mmol), EDC (5.72 g, 29.8 mmol), 1-hydroxy-benzotriazole (4.04 g, 29.9 mmol) then triethylamine (2.08 mL, 14.9 mmol). The reaction mixture was stirred for 14 h then the solution was washed with hydrochloric acid (1.0 M, 2 × 100 mL), saturated aqueous $NaHCO_3$ solution (2 × 100 mL) and brine (100 mL). The solution was then dried ($MgSO_4$), filtered, and the solvent removed *in vacuo* yielding the title compound as a yellow solid (4.50 g, ~quantitative) which was used without further purification. R_f 0.50 (petrol/ethyl acetate, 50:50); m.p. 72–75 °C; IR ν_{max} (thin film)/cm^{-1} 3267s, 3032m, 2947m, 1635s, 1454s, 1261s, 1242m; ^1H NMR (400 MHz, $CDCl_3$) δ_H 7.37 – 7.26 (5H, m), 5.58 (1H, br. s), 4.43 (2H, d, J = 5.5 Hz), 2.85 (1H, quin, J = 8.5 Hz), 2.31 – 2.11 (4H, m), 2.02 (2H, t, J = 7.0 Hz), 1.92 (2H, t, J = 7.5 Hz), 1.87 – 1.76 (2H, m); ^{13}C NMR (101 MHz, $CDCl_3$) δ_C 174.9, 138.6, 128.9, 128.0, 127.6, 43.7, 40.0, 38.1, 35.3, 34.9, 34.4, 16.4; HRMS (APCI+) *m/z* $[M+H]^+$ calcd for $C_{15}H_{20}NO$, 230.1539: found: 230.1534.

S4.2 General procedure for preparative enzymatic hydroxylations

A solution of substrate **1** in the solvent specified was added in aliquots to a solution of the $P450_{BM3}$ variant (in phosphate buffer, pH 8.0), glucose (powder or 1.0 M solution in phosphate buffer), GDH (400 U/mL solution in phosphate buffer), $NADP^+$ monosodium salt (40.0 mM solution in phosphate buffer) and cyclodextrin (0.2 M solution in phosphate buffer) in phosphate buffer with air bubbling. The pH of the reaction mixture was maintained above 7.80 by periodic addition of aqueous KOH solution (3.0 M). Ethyl acetate (volume equivalent to the reaction volume) was added then the biphasic mixture was transferred into 50 mL centrifuge tubes, shaken for 30 s, centrifuged at 10,000 *g* for 3 min, and the organic layer separated. This process was repeated twice more then the combined organic extracts were washed sequentially with saturated aqueous $NaHCO_3$ solution, water, and brine, then dried ($MgSO_4$), filtered, and concentrated *in vacuo* to give the crude product which was then purified by column chromatography.

(1) Opie, C. R.; Noda, H.; Shibasaki, M.; Kumagai, N. *Chem. Eur. J.* **2019**, *25*, 4648–4653.

*S4.3 (2S,4r,5R)-N-Benzyl-5-hydroxyspiro[3.3]heptane-2-carboxamide (**2**)*

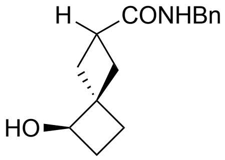

Prepared according to the general procedure, using variant K19/F87I/I263G/A330I (6.0 mL, 233.0 μM), glucose powder (2.0 g), GDH (0.50 mL), $NADP^+$ monosodium salt (0.25 mL), in phosphate buffer (200 mL), and substrate **1** (1.3 mL, 0.4 M in ethanol, 0.52 mmol) stirring for 14 h. Purification by column chromatography (petrol/ethyl acetate, 90:10 → 35:65) yielded the title compound as a colorless oil (48.9 mg, 38%). R_f 0.50 (ethyl acetate); $[\alpha]_D^{25}$ –4.8 (*c* 0.4, dichloromethane); IR ν_{max} (thin film)/cm^{-1} 3290s, 2927s, 1643s, 1531s, 1427m, 1238s; ^1H NMR (400 MHz, $CDCl_3$) δ_H 7.36 – 7.27 (3H, m), 7.26 – 7.23 (2H, m), 5.59 (1H, br. s), 4.44 (2H, d, *J* = 5.5 Hz), 4.01 (1H, t, *J* = 7.5 Hz), 2.85 (1H, quin, *J* = 8.5 Hz), 2.65 (1H, ddd, *J* = 11.0, 8.5, 4.5 Hz), 2.30 (1H, dd, *J* = 11.5, 8.0 Hz), 2.22 – 2.09 (3H, m), 1.73 – 1.54 (3H, m), 1.47 (1H, ddd, *J* = 11.5, 8.5, 8.0 Hz); ^{13}C NMR (101 MHz, $CDCl_3$) δ_C 174.7, 138.5, 128.9, 128.0, 127.7, 73.0, 45.9, 43.8, 35.5, 35.2, 30.2, 28.6, 25.7; HRMS (APCI+) *m/z* $[M+H]^+$ calcd for $C_{15}H_{20}NO_2$, 246.1489: found: 246.1473.

*S4.4 (2R,4r,6R)-N-Benzyl-6-hydroxyspiro[3.3]heptane-2-carboxamide (**3**)*

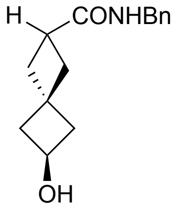

Prepared according to the general procedure, using variant GVQ/I263G/A330F (91.0 mL, 26.8 μM), glucose powder (1.8 g), GDH (2.0 mL, added in portions), $NADP^+$ monosodium salt (1.0 mL, added in portions), cyclodextrin (30 mL) in phosphate buffer (200 mL), and substrate **1** (2.88 mL, 0.4 M in ethanol, 1.15 mmol) stirring for 14 h. Purification by column chromatography (petrol/ethyl acetate, 90:10 → 35:65) afforded the title compound as a colorless solid (101.4 mg, 36%). R_f 0.40 (ethyl acetate); m.p. 70–74 °C; $[\alpha]_D^{25}$ –4.2 (*c* 0.6, dichloromethane); IR ν_{max} (thin film)/cm^{-1} 3250br s, 3059s, 2924s, 1639s, 1427m, 1265s; ^1H NMR (400 MHz, $CDCl_3$) δ_H 7.38 – 7.27 (3H, m), 7.26 – 7.24 (2H, m), 5.57 (1H, br. s), 4.42 (2H, app. d, *J* = 5.5 Hz), 4.21 – 4.14 (1H, m), 2.90 (1H, quin, *J* = 8.5 Hz), 2.53 – 2.44 (1H, m), 2.40 – 2.29 (3H, m), 2.20 – 2.09 (2H, m), 1.93 (1H, dd, *J* = 11.5, 7.5 Hz), 1.88 (1H, dd, *J* = 11.5, 8.0 Hz); ^{13}C NMR (101 MHz, $CDCl_3$) δ_C 174.5, 138.5, 128.9, 128.0, 127.7, 63.2, 46.0, 45.4, 43.8, 37.9, 37.3, 35.5, 31.2; HRMS (APCI+) *m/z* $[M+H]^+$ calcd for $C_{15}H_{20}NO_2$, 246.1489: found: 246.1479.

*S4.5 (2S,4s,6S)-N-Benzyl-6-hydroxyspiro[3.3]heptane-2-carboxamide (ent-**3**)*

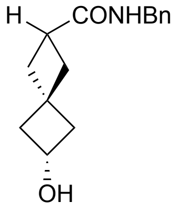

Prepared according to the general procedure, using variant R19 (6.0 mL, 87.0 μM), glucose powder (0.5 g), GDH (0.25 mL), $NADP^+$ monosodium salt (125 μL) in phosphate buffer (200 mL), and substrate **1** (0.75 mL, 0.4 M in ethanol, 0.30 mmol) stirring for 14 h. Purification by column chromatography (petrol/ethyl acetate, 90:10 → 35:65) yielded the title compound as a pale yellow oil (5.8 mg, 8%). Data as above except $[\alpha]_D^{25}$ +4.2 (*c* 0.1, dichloromethane).

*S4.6 (2R,4s,5R)-N-Benzyl-5-hydroxyspiro[3.3]heptane-2-carboxamide (**5**)*

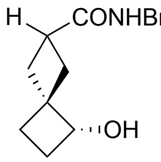

Prepared according to the general procedure, using variant R19/F87A/L75F (14.0 mL, 120.0 μM), glucose powder (1.0 g), GDH (0.50 mL), $NADP^+$ monosodium salt (0.25 mL), in phosphate buffer (200 mL), and substrate **1** (2.75 mL, 0.4 M in ethanol, 1.10 mmol) stirring for 14 h. Purification by column chromatography (petrol/ethyl acetate, 90:10 → 35:65) afforded the title compound as a colorless oil (80.7 mg, 30%). R_f 0.60 (ethyl acetate); $[\alpha]_D^{25}$ –16.5 (*c* 0.6, dichloromethane); IR ν_{max} (thin film)/cm^{-1} 3310s, 2935s, 1639s, 1546m, 1454w, 1269s; ^1H NMR (400 MHz, $CDCl_3$) δ_H 7.35 – 7.28 (3H, m), 7.27 – 7.23 (2H, m), 5.99 (1H, br. s), 4.44 (1H, dd, *J* = 14.5, 5.5 Hz) overlapping 4.40 (1H, dd, *J* = 14.5, 5.5 Hz), 4.05 (1H, t, *J* = 7.5 Hz), 2.85 – 2.74 (2H, m), 2.29 – 2.20 (1H, m), 2.15 – 2.01 (3H, m), 1.70 – 1.58 (2H, m), 1.56 – 1.45 (1H, m); ^{13}C NMR (101 MHz, $CDCl_3$) δ_C 177.4, 138.1, 129.0, 128.0, 127.8, 72.7, 46.5, 43.9, 35.7, 35.1, 30.2, 26.7, 26.4; HRMS (APCI+) *m/z* $[M+H]^+$ calcd for $C_{15}H_{20}NO_2$, 246.1489: found: 246.1479.

*S4.7 N-benzyl-2,6-dihydroxyspiro[3.3]heptane-2-carboxamide (**4**) and N-Benzyl-2-hydroxyspiro[3.3]heptane-2-carboxamide (**6**)*

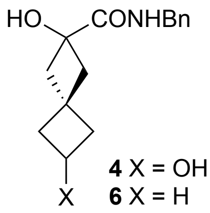

4 X = OH
6 X = H

Prepared according to the general procedure, using variant GVQ/A330W (26.0 mL, 113.0 μM), glucose powder (3.0 g), GDH (3.0 mL), $NADP^+$ monosodium salt (1.5 mL), in phosphate buffer (400 mL), and substrate **1** (3.20 mL, 0.4 M in ethanol, 1.3 mmol) stirring for 14 h. Purification by column chromatography (petrol/ethyl acetate, 90:10 → 35:65) yielded the 2-hydroxylated product **6** as a colorless solid (20.0 mg, 7%). R_f 0.20 (petrol/ethyl acetate, 67:33); m.p. 69 – 71 °C; IR ν_{max} (thin film)/cm^{-1} 2974s, 2889m, 1654m, 1454m, 1381m, 1087s; ^1H NMR (400 MHz, $CDCl_3$) δ_H 7.36 – 7.24 (5H, m), 6.81 (1H, br. s), 4.40 (2H, d, *J* = 6.0 Hz), 2.78 – 2.71 (2H, m), 2.62 (1H, s), 2.16 – 2.06 (6H, m), 1.87 – 1.77 (2H, m); ^{13}C NMR (101 MHz, $CDCl_3$)

δ_C 175.0, 138.3, 128.9, 127.9, 127.7, 72.9, 47.8, 43.6, 36.3, 35.7, 34.7, 16.4; HRMS (APCI+) *m/z* $[M+H]^+$ calcd for $C_{15}H_{19}NaNO_2$, 268.1308: found: 268.1303. The 2,6-dihydroxylated product **4** was also isolated, as a yellow oil (72.5 mg, 22%). R_f 0.30 (ethyl acetate); IR ν_{max} (thin film)/cm^{-1} 3352s, 2954s, 2252w, 1674m, 1519s; ^{1}H NMR (400 MHz, $CDCl_3$) δ_H 7.36 – 7.24 (5H, m), 6.83 (1H, s), 4.44 (2H, d, *J* = 5.5 Hz), 4.21 (1H, quin, *J* = 7.0 Hz), 2.81 – 2.73 (2H, m), 2.65 – 2.59 (1H, m), 2.53 – 2.47 (1H, m), 2.16 – 2.11 (2H, m), 2.07 – 1.98 (2H, m); ^{13}C NMR (101 MHz, $CDCl_3$) δ_C 174.5, 138.3, 128.9, 128.0, 127.7, 73.3, 63.1, 47.7, 47.0, 46.9, 45.6, 43.5, 27.1; HRMS (APCI+) *m/z* $[M+H]^+$ calcd for $C_{15}H_{19}NaNO_3$, 284.1257: found: 284.1265.

S4.8 Gram-scale reaction with GVQ/I263G/A330F

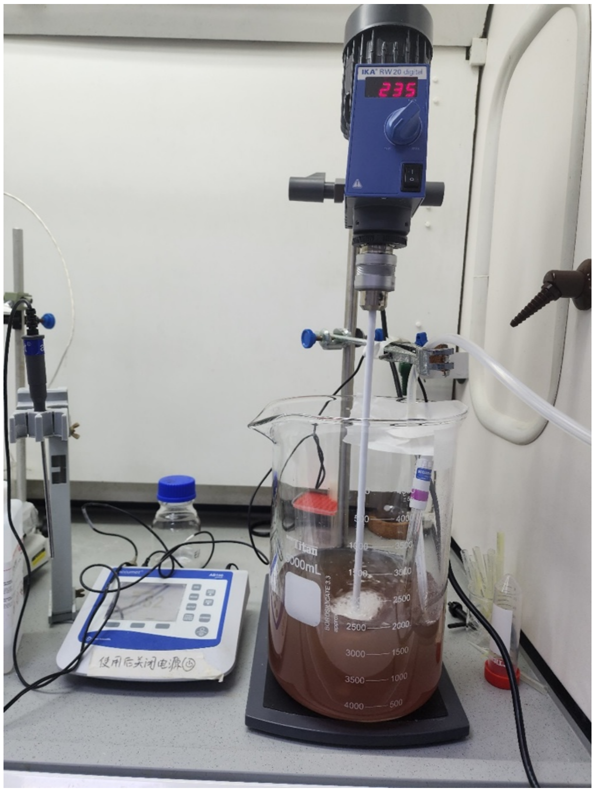

The image shows the reaction set-up with overhead stirrer, pH probe, and air bubbler. The substrate **1** (1.50 g, 6.54 mmol, dissolved in the minimum amount of ethanol) was added to a stirred 20 °C solution containing: variant GVQ/I263G/A330F (113 mL, 161 µM), glucose powder (10 g), GDH (6.0 mL), NADP$^+$ monosodium salt (1.5 mL), and cyclodextrin (108 mL) in phosphate buffer (558 mL). The pH of the reaction mixture was maintained above 7.90 by periodic addition of aqueous KOH solution (3.0 M). After 2.5 h, the mixture was extracted with ethyl acetate (3 × 800 mL) and the combined organic extracts were washed successively with saturated aqueous $NaHCO_3$ solution, water, and brine, then dried ($MgSO_4$), filtered, and concentrated *in vacuo*. The residue was purified by column chromatography (pentane/ethyl acetate, 90:10 → 35:65) to yield (2*R*,4*r*,6*R*)-**3** as a colorless solid (1.04 g, 65%), and (2*S*,4*r*,5*R*)-**2** (347 mg, 22%). Data as above.

S4.9 Gram-scale reaction with R19/F87A/L75F

The reaction set-up depicted above was also used for this procedure. The substrate (1.53 g, 6.87 mmol, dissolved in the minimum amount of ethanol) was added portionwise (~0.4–0.8 mmole every hour) to a stirred 20 °C solution containing: variant R19/L75F/F87A (44 mL, 119 µM), glucose powder (5.0 g), GDH (2.0 mL), NADP$^+$ monosodium salt (1.0 mL),

and cyclodextrin (100 mL) in phosphate buffer (1.5 L). The pH of the reaction mixture was maintained above 7.90 by periodic addition of aqueous KOH solution (3.0 M). During this time, more GDH (2.0 mL) and $NADP^+$ monosodium salt (1.0 mL) were added to the reaction mixture (twice). After 14 h, the mixture was extracted with ethyl acetate (3 × 1.5 L) and the combined organic extracts were washed successively with saturated aqueous $NaHCO_3$ solution, water, and brine, then dried ($MgSO_4$), filtered, and concentrated *in vacuo*. The residue was purified by column chromatography (pentane/ethyl acetate, 90:10 → 35:65) to yield (*2R,4s,5R*)-**5** (721 mg, 43%) and a mixture of (2*S*,4*r*,5*R*)-**2** and (2*R*,4*r*,6*R*)-**3** (299 mg, 18%) in 33:67 ratio, respectively. Data as above.

S4.10 General procedure A (preparation of ketones 7–9)

A 0.01 M solution of the alcohol (**2**, **3**, or **5**) in dichloromethane was stirred at room temperature. Dess–Martin periodinane (DMP) (1.5 equiv.) and $NaHCO_3$ (2.3 equiv.) were added then stirring was continued for 14 h. The reaction mixture was transferred to a separatory funnel and washed sequentially with saturated aqueous $Na_2S_2O_3$ solution and brine, then dried ($MgSO_4$) and filtered. The solvent was removed *in vacuo* to give the crude product (**7**, **8**, or **9**, respectively) which was purified by column chromatography.

*S4.11 General procedure B (preparation of azidoformates **10–12**)*

A 0.25 M solution of the alcohol (**2**, **3**, or **5**) in tetrahydrofuran was stirred at room temperature. Carbonyldiimidazole (2.0 equiv.) was added, the mixture was stirred for 30 min, then azidotrimethylsilane (10–15 equiv.) was added. The mixture was stirred for 14 h then transferred to a separatory funnel and diluted with water. The mixture was extracted thrice with ethyl acetate, filtered through a pad of silica, then dried ($MgSO_4$), and filtered. The solvent was removed *in vacuo* to give the crude product (**10**, **11**, or **12**, respectively) which was purified by column chromatography.

*S4.12 General procedure C (preparation of Mosher's esters **S1–S6**)*

A 0.064 M solution of the alcohol (**2**, **3**, or **5**) in dichloromethane was stirred at room temperature. Pyridine (3.1 equiv.) was added followed by the appropriate enantiomer of α-methoxy-α-trifluoromethylphenylacetyl chloride (MTPA-Cl) (1.9 equiv.). The reaction mixture was stirred for 14 h then transferred to a separatory funnel, shaken with an equal volume of water, then the separated aqueous layer was extracted twice with dichloromethane; the combined organic

portions were dried ($MgSO_4$), filtered, and the solvent removed *in vacuo* to give the crude ester which was purified by column chromatography, taking care not to separate diastereomers (if any).

S4.13 trans-N-Benzyl-5-oxospiro[3.3]heptane-2-carboxamide (7)

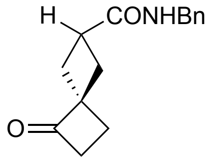

Prepared according to general procedure A from alcohol **2** (18.6 mg, 0.076 mmol). Purification by column chromatography (petrol/ethyl acetate, 95:5 → 90:10) gave the title compound as a colorless solid (16.0 mg, 87%). R_f 0.50 (petrol/ethyl acetate, 50:50); m.p. 109–112 °C; IR ν_{max} (thin film)/cm^{-1} 2924 s, 2850 m, 1770 s, 1643 s, 1420 m; ^{1}H NMR (400 MHz, $CDCl_3$) δ_H 7.38 – 7.28 (3H, m), 7.26 – 7.21 (2H, m), 5.71 (1H, br. s), 4.42 (2H, d, *J* = 5.5 Hz), 3.05 – 2.91 (1H, m) overlapping 2.94 (2H, t, *J* = 8.5 Hz), 2.54 – 2.41 (4H, m), 2.08 – 2.01 (2H, m); ^{13}C NMR (101 MHz, $CDCl_3$) δ_C 214.9, 173.6, 138.2, 128.9, 127.9, 127.7, 60.2, 43.8, 43.1, 34.7, 33.0, 24.1; HRMS (ESI+) *m/z* $[M+H]^+$ calcd for $C_{15}H_{18}NO_2$, 244.1332: found: 244.1327.

*S4.14 N-Benzyl-6-oxospiro[3.3]heptane-2-carboxamide (**8**)*

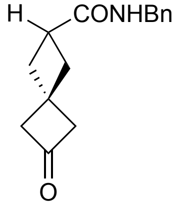

Prepared according to general procedure A from alcohol **3** (15.0 mg, 0.061 mmol). Purification by column chromatography (petrol/ethyl acetate, 95:5 → 90:10) gave the title compound as a colorless solid (13.5 mg, 91%). R_f 0.50 (petrol/ethyl acetate, 50:50); m.p. 94–96 °C; IR ν_{max} (thin film)/cm^{-1} 3200 br s, 1778 s, 1639 s, 1540 m, 1250 m; ^{1}H NMR (400 MHz, $CDCl_3$) δ_H 7.37 – 7.24 (5H, m), 5.72 (1H, br. s), 4.44 (2H, d, *J* = 5.5 Hz), 3.13 – 3.07 (4H, m), 2.99 (1H, quin, *J* = 8.5 Hz), 2.65 – 2.57 (2H, m), 2.41 – 2.35 (2H, m); ^{13}C NMR (101 MHz, $CDCl_3$) δ_C 207.2, 173.9, 138.3, 128.9, 128.0, 127.8, 59.3, 58.8, 43.8, 37.0, 34.9, 29.7; HRMS (ESI+) *m/z* $[M+H]^+$ calcd for $C_{15}H_{18}NO_2$, 244.1332: found: 244.1327.

*S4.15 cis-N-Benzyl-5-oxospiro[3.3]heptane-2-carboxamide (**9**)*

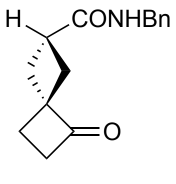

Prepared according to general procedure A from alcohol **5** (14.0 mg, 0.057 mmol). Purification by column chromatography (petrol/ethyl acetate, 95:5 → 90:10) gave the title compound as a colorless solid (6.0 mg, 43%). R_f 0.60 (ethyl acetate); m.p. 107–113 °C; IR ν_{max} (thin film)/cm^{-1} 2927 br s, 1753 s, 1643 m, 1531 m,1377 m, 1249 s; ^{1}H NMR (400 MHz, $CDCl_3$) δ_H 7.38 – 7.25 (5H, m), 6.23 (1H, br. s), 4.46 (2H, d, *J* = 5.5 Hz), 3.08 – 2.95 (1H, m) overlaying 2.98 (2H, t, *J* = 8.5 Hz), 2.71 – 2.64 (2H, m), 2.42 – 2.33 (2H, m), 2.16 (2H,

~t, J = 8.5 Hz); ^{13}C NMR (101 MHz, $CDCl_3$) δ_C 212.7, 173.9, 138.4, 128.8, 128.0, 127.6, 59.5, 43.8, 43.3, 33.9, 33.4, 25.8; HRMS (ESI+) *m/z* $[M+H]^+$ calcd for $C_{15}H_{18}NO_2$, 244.1332: found: 244.1328.

*S4.16 (1R,4r,6S)-6-(Benzylcarbamoyl)spiro[3.3]heptan-1-yl carbonazidate (**10**)*

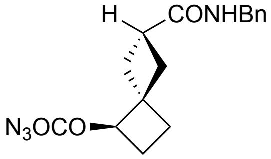

Prepared according to general procedure B from alcohol **2** (79.0 mg, 0.322 mmol). Purification by column chromatography (petrol/ethyl acetate, 90:10) gave the title compound as a colorless solid (86.4 mg, 85%). R_f 0.70 (petrol/ethyl acetate, 50:50); m.p. 70–72 °C; $[\alpha]_D^{25}$ +16.3 (*c* 0.2, chloroform); IR ν_{max} (thin film)/cm^{-1} 3016s, 2179s, 2133s, 1724s, 1662m, 15243m, 1238s, 1219s; ^{1}H NMR (400 MHz, $CDCl_3$) δ_H 7.38 – 7.23 (5H, m), 5.58 (1H, br. s), 4.87 (1H, t, J = 8.0 Hz), 4.48 – 4.37 (2H, m), 2.80 (1H, quin, J = 8.5 Hz), 2.58 (1H, ddd, J = 11.5, 8.5, 3.0 Hz), 2.38 – 2.29 (2H, m), 2.30 – 2.18 (2H, m), 1.98 – 1.85 (2H, m), 1.70 – 1.61 (1H, m); ^{13}C NMR (101 MHz, $CDCl_3$) δ_C 174.3, 157.3, 138.4, 128.9, 128.0, 127.7, 78.1, 45.0, 43.8, 35.2, 35.0, 31.0, 26.8, 24.7; HRMS (ESI+) *m/z* $[M+H]^+$ calcd for $C_{16}H_{19}N_4O_3$, 315.1452: found: 315.1450.

*S4.17 (2R,4r,6R)-6-(Benzylcarbamoyl)spiro[3.3]heptan-2-yl carbonazidate (**11**)*

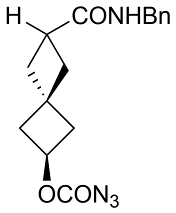

Prepared according to general procedure B from alcohol **3** (36.6 mg, 0.149 mmol). Purification by column chromatography (petrol/ethyl acetate, 90:10) gave the title compound as a colorless solid (25.9 mg, 55%). R_f 0.70 (petrol/ethyl acetate, 50:50); m.p. 70–74 °C; $[\alpha]_D^{25}$ –3.1 (*c* 0.6, chloroform); IR ν_{max} (thin film)/cm^{-1} 3055br, 2978s, 2183s, 2137s, 1724s, 1662s, 1523m, 1265s, 1242s; ^{1}H NMR (400 MHz, $CDCl_3$) δ_H 7.36 – 7.23 (5H, m), 5.61 (1H, br. s), 4.92 (1H, quin, J = 7.0 Hz), 4.42 (2H, d, J = 5.5 Hz), 2.89 (1H, quin, J = 8.5 Hz), 2.60 – 2.53 (1H, m), 2.50 – 2.43 (1H, m), 2.42 – 2.35 (2H, m), 2.25 – 2.10 (4H, m); ^{13}C NMR (101 MHz, $CDCl_3$) δ_C 174.4, 157.2, 138.7, 129.2, 128.3, 128.0, 69.4, 44.1, 42.9, 42.0, 37.8, 37.6, 35.5, 32.9; HRMS (ESI+) *m/z* $[M+H]^+$ calcd for $C_{16}H_{19}N_4O_3$, 315.1452: found: 315.1450.

*S4.18 (1R,4s,6R)-6-(Benzylcarbamoyl)spiro[3.3]heptan-1-yl carbonazidate (**12**)*

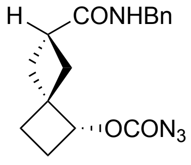

Prepared according to general procedure B from alcohol **5** (40.0 mg, 0.163 mmol). Purification by column chromatography (petrol/ethyl acetate, 90:10) gave the title compound as a colorless solid (7.8 mg, 16%). R_f 0.70 (petrol/ethyl acetate, 50:50); m.p. 58–62 °C; $[\alpha]_D^{25}$ +9.8 (*c* 0.3, chloroform); IR ν_{max} (thin film)/cm^{-1} 3016s, 2939s, 2183m, 2133m, 1724m, 1531m, 1234s, 1219s; ^{1}H NMR (400 MHz, $CDCl_3$) δ_H 7.36 – 7.24

(5H, m), 5.70 (1H, br. s), 4.78 (1H, t, J = 7.5 Hz), 4.43 (2H, d, J = 5.5 Hz), 2.94 (1H, quin, J = 9.0 Hz), 2.64 – 2.57 (1H, m), 2.48 – 2.41 (1H, m), 2.30 – 2.22 (1H, m), 2.14 – 2.05 (2H, m), 2.00 – 1.88 (2H, m), 1.75 – 1.68 (1H, m); ^{13}C NMR (101 MHz, $CDCl_3$) δ_C 174.0, 157.2, 138.5, 128.9, 128.0, 127.7, 76.0, 44.2, 43.7, 34.5, 34.3, 31.8, 27.3, 25.0; HRMS (ESI+) *m/z* $[M+H]^+$ calcd for $C_{16}H_{19}N_4O_3$, 315.1452: found: 315.1436.

*S4.19 Thermolysis of azidoformate **10***

A solution of azidoformate **10** (32.0 mg, 0.102 mmol) in 1,1,2,2-tetrachloroethane (1 mL) was heated in a pressure tube at 140 °C for 2 h. After cooling the reaction mixture to RT, the solvent was removed *in vacuo* and the residue was purified by column chromatography (dichloromethane/methanol, 99:1 → 97.5:2.5) to afford three products: ketone **7** (4.0 mg, 16%), carbamate **13** (4.0 mg, 14%, pale yellow paste), and carbamate **14** (4.1 mg, 14%, pale yellow paste).

Data for **7**: as above, §S4.13.

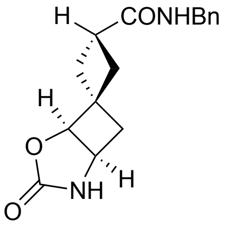

Data for (1*R*,3'*S*,5*S*,6*r*)-*N*-benzyl-3-oxo-4-oxa-2-azaspiro(bicyclo[3.2.0]heptane-6,1'-cyclobutane)-3'-carboxamide (**13**): R_f 0.25 (methanol/dichloromethane, 1:99); $[\alpha]_D^{25}$ –27.6 (*c* 0.3, dichloromethane); IR ν_{max} (thin film)/cm^{-1} 3502br s, 2978s, 2376m, 2314m, 1639s, 1512s, 1427m, 1219m; ^{1}H NMR (400 MHz, $CDCl_3$) δ_H 7.35 – 7.22 (5H, m), 5.64 (1H, br. s), 5.28 (1H, br. s), 4.89 (1H, d, J = 7.0 Hz), 4.42 (2H, d, J = 5.5 Hz), 4.18 (1H, dddd, J = 7.0, 5.5, 2.5, 1.5 Hz), 2.86 (1H, quin, J = 8.5 Hz), 2.70 (1H, ddd, J = 11.5, 8.5, 4.5 Hz), 2.42 (1H, dd, J = 11.5, 8.5 Hz), 2.31 – 2.22 (2H, m), 2.20 (1H, dd, J = 11.5, 8.5 Hz), 2.11 (1H, dd, J = 13.5, 2.5 Hz); ^{13}C NMR (101 MHz, $CDCl_3$) δ_C 173.8, 160.6, 138.3, 128.9, 127.8, 127.6, 81.8, 49.6, 43.9, 43.7, 40.3, 35.7, 35.2, 31.5; HRMS (APCI+) *m/z* $[M+H]^+$ calcd for $C_{16}H_{19}N_2O_3$, 287.1390: found: 287.1378.

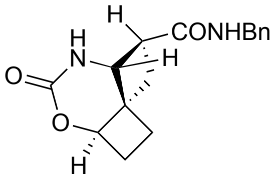

Data for (1*S*,4*R*,8*R*,9*S*)-*N*-benzyl-6-oxo-5-oxa-7-azatricyclo[6.2.0.0^{1,4}]decane-9-carboxamide (**14**): R_f 0.39 (methanol/dichloromethane, 1:99); $[\alpha]_D^{25}$ +3.0 (*c* 0.3, dichloromethane); IR ν_{max} (thin film)/cm^{-1} 3055br s, 2985s, 2939s, 1708s, 1531m, 1265s, 1219m, 1165m; ^{1}H NMR (400 MHz, $CDCl_3$) δ_H 7.35 – 2.23 (5H, m), 6.38 (1H, br. s), 6.00 (1H, br. s), 4.68 (1H, t, J = 8.0 Hz), 4.45 – 4.34 (2H, m), 3.94 (1H, dd, J = 7.0, 4.5 Hz), 2.80 (1H, dt, J = 9.5, 7.0 Hz), 2.31 (1H, dddd, J = 12.0, 9.0, 7.5, 3.0 Hz), 2.15 – 2.01 (3H, m), 1.89 (1H, tdd, J = 11.5, 3.0, 1.5 Hz), 1.71 (1H, ddd, J = 11.5, 10.5, 9.5 Hz); ^{13}C NMR (101 MHz, $CDCl_3$) δ_C 171.6, 153.7, 138.2, 128.9, 128.0, 127.7, 78.4, 56.0, 45.4, 43.9, 43.8, 27.7, 26.4, 22.5; HRMS (APCI+) *m/z* $[M+H]^+$ calcd for $C_{16}H_{19}N_2O_3$, 287.1390: found: 287.1376.

S4.20 Thermolysis of azidoformate ***11***

A solution of azidoformate **11** (7.5 mg, 0.024 mmol) in 1,1,2,2-tetrachloroethane (1 mL) was heated in a pressure tube at 140 °C for 3 h. After cooling the reaction mixture to RT, the solvent was removed *in vacuo* and the residue was purified by column chromatography (dichloromethane/methanol, 99:1 → 97.5:2.5) to afford three products: ketone **8** (1.0 mg, 17%), carbamate **15** (3.0 mg, 44%, yellow paste), and carbamate **16** (2.0 mg, 29%, pale yellow paste).

Data for **8**: as above, §S4.14.

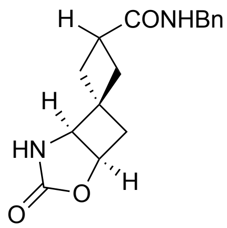

Data for (1*R*,3'*S*,5*S*,6*r*)-*N*-benzyl-3-oxo-2-oxa-4-azaspiro(bicyclo[3.2.0]heptane-6,1'-cyclobutane)-3'-carboxamide (**15**): R_f 0.33 (ethyl acetate); $[\alpha]_D^{25}$ +51.5 (*c* 0.1, chloroform); IR ν_{max} (thin film)/cm^{-1} 3286br s, 2927s, 1739s, 1647m, 1546m, 1454m, 1323m, 1295m; ^{1}H NMR (400 MHz, $CDCl_3$) δ_H 7.38 – 7.23 (5H, m), 5.68 (2H, br. s), 4.93 (1H, td, *J* = 6.5, 3.5 Hz), 4.42 (2H, d, *J* = 5.5 Hz), 4.22 (1H, dq, *J* = 7.0, 1.5 Hz), 2.83 (1H, tt, *J* = 8.5, 4.5 Hz), 2.51 – 2.43 (2H, m), 2.35 – 2.20 (3H, m), 2.11 (1H, dd, *J* = 12.5, 8.5 Hz); ^{13}C NMR (101 MHz, $CDCl_3$) δ_C 175.2, 160.4, 138.2, 128.9, 127.9, 127.8, 72.7, 61.4, 43.8, 43.3, 41.6, 36.1, 35.4, 31.6; HRMS (APCI+) *m/z* $[M+H]^+$ calcd for $C_{16}H_{19}N_2O_3$, 287.1390: found: 287.1380.

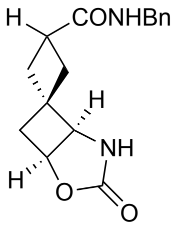

Data for (1*S*,3'*S*,5*R*,6*s*)-*N*-Benzyl-3-oxo-2-oxa-4-azaspiro(bicyclo[3.2.0]heptane-6,1'-cyclobutane)-3'-carboxamide (**16**): R_f 0.19 (ethyl acetate); $[\alpha]_D^{25}$ –47.3 (*c* 0.4, chloroform); IR ν_{max} (thin film)/cm^{-1} 3286br s, 2974s, 2927s, 1710s, 1647m, 1543m, 1377m, 1215m; ^{1}H NMR (400 MHz, $CDCl_3$) δ_H 7.36 – 7.22 (5H, m), 6.29 (1H, br. s), 5.67 (1H, br. s), 4.97 (1H, td, *J* = 6.5, 4.0 Hz), 4.42 (2H, d, *J* = 5.5 Hz), 4.16 (1H, dquin, *J* = 1.5 Hz), 2.79 (1H, quin, *J* = 8.5 Hz), 2.49 (1H, ddd, *J* = 14.5, 6.5, 2.5 Hz), 2.42 – 2.31 (3H, m), 2.25 – 2.15 (2H, m); ^{13}C NMR (101 MHz, $CDCl_3$) δ_C 173.6, 161.0, 138.2, 128.9, 128.0, 127.8, 72.6, 61.3, 43.8, 41.5 (two peaks), 35.8, 34.6, 31.6; HRMS (APCI+) *m/z* $[M+H]^+$ calcd for $C_{16}H_{19}N_2O_3$, 287.1390: found: 287.1382.

S4.21 Thermolysis of azidoformate ***12***

A solution of azidoformate **12** (94.0 mg, 0.299 mmol) in 1,1,2,2-tetrachloroethane (3 mL) was heated in a pressure tube at 140 °C for 2 h. After cooling the reaction mixture to RT, the solvent was removed *in vacuo* and the residue was purified by column chromatography (dichloromethane/methanol, 99:1 → 97.5:2.5) to afford two products: ketone **9** (25.7 mg, 35%) and carbamate **17** (8.8 mg, 10%, pale yellow paste).

Data for **9**: as above, §S4.15.

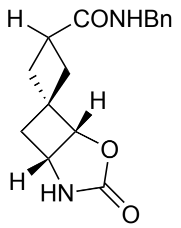

Data for (1*R*,3'*R*,5*S*,6*s*)-*N*-benzyl-3-oxo-4-oxa-2-azaspiro[bicyclo[3.2.0]heptane-6,1'-cyclobutane]-3'-carboxamide (**17**): R_f 0.25 (methanol/dichlorometh-ane, 4:96); $[\alpha]_D^{25}$ –87.4 (*c* 0.3, dichloromethane); IR ν_{max} (thin film)/cm^{-1} 3099 br s, 2950m, 2355m, 1750m, 1497s, 1232s; ^{1}H NMR (400 MHz, $CDCl_3$) δ_H 7.36 – 7.21 (5H, m), 5.89 (1H, br. s), 5.84 (1H, br. s), 4.82 (1H, d, *J* = 6.5 Hz), 4.41 (2H, d, *J* = 5.5 Hz), 4.17 (1H, br. t, *J* = 6.0 Hz), 2.93 (1H, quin, *J* = 8.5 Hz), 2.70 (1H, dd, *J* = 13.0, 8.0 Hz), 2.49 (1H, dd, *J* = 13.0, 8.0 Hz), 2.33 (1H, dd, *J* = 13.5, 5.5 Hz), 2.23 – 2.11 (3H, m); ^{13}C NMR (101 MHz, $CDCl_3$) δ_C 173.8, 160.8, 138.4, 128.9, 128.0, 127.7, 81.0, 49.5, 43.8, 43.5, 41.5, 35.6, 34.2, 32.3; HRMS (APCI+) *m/z* $[M+H]^+$ calcd for $C_{16}H_{19}N_2O_3$, 287.1390: found: 237.1380.

*S4.22 (1R,4r,6S)-6-(Benzylcarbamoyl)spiro[3.3]heptan-1-yl (S)-3,3,3-trifluoro-2-methoxy-2-phenylpropanoate (**S1**)*

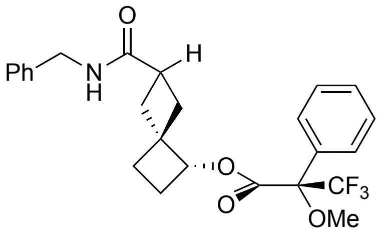

Prepared according to general procedure C from alcohol **2** (17.0 mg, 0.069 mmol) and (*R*)-(−)-MTPA-Cl. Purification by column chromatography (petrol/ethyl acetate, 80:20) gave the title compound as a colorless solid (22.3 mg, 70%). R_f 0.70 (petrol/ethyl acetate, 50:50); $[\alpha]_D^{25}$ –40.2 (*c* 0.1, dichloromethane); IR ν_{max} (thin film)/cm^{-1} 3020 br s, 2947s, 1747m, 1651m, 1531m, 1219s, 1125m; ^{1}H NMR (400 MHz, $CDCl_3$) δ_H 7.58 – 7.54 (2H, m), 7.39 – 7.22 (8H, m), 5.37 (1H, br. s), 5.10 (1H, t, *J* = 7.5 Hz), 4.44 – 4.33 (2H, m), 3.57 (3H, q, *J* = 1.0 Hz), 2.48 (1H, quin, *J* = 8.5 Hz), 2.43 – 2.36 (1H, m), 2.35 – 2.21 (3H, m), 2.16 (1H, dd, *J* = 11.5, 8.0 Hz), 2.00 – 1.82 (2H, m), 1.71 – 1.62 (1H, m); ^{13}C NMR (101 MHz, $CDCl_3$) δ_C 174.2, 166.3, 138.4, 132.6, 129.8, 128.9, 128.6, 128.0, 127.8, 127.4, 123.6 (q, *J* = 289 Hz), 76.0, 55.6, 45.2, 43.8, 35.0, 34.9, 31.3, 26.7, 25.1 (**C**CF_3 not resolved); HRMS (ESI+) *m/z* $[M+H]^+$ calcd for $C_{25}H_{27}F_3NO_4$, 462.1887: found: 462.1886.

*S4.23 (1R,4r,6S)-6-(Benzylcarbamoyl)spiro[3.3]heptan-1-yl (R)-3,3,3-trifluoro-2-methoxy-2-phenylpropanoate (**S2**)*

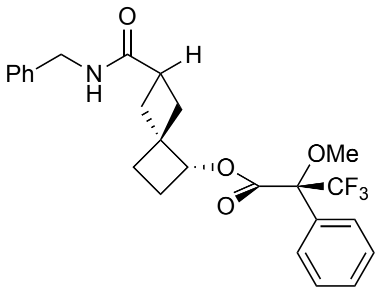

Prepared according to general procedure C from alcohol **2** (25.0 mg, 0.102 mmol) and (*S*)-(+)- MTPA-Cl. Purification by column chromatography (petrol/ethyl acetate, 80:20) gave the title compound as a colorless solid (16.5 mg, 35%). R_f 0.70 (petrol/ethyl acetate, 50:50); $[\alpha]_D^{25}$ +14.7 (*c* 0.3, dichloromethane); IR ν_{max} (thin film)/cm^{-1} 3310 br s, 2974s, 2935s, 2889s, 1747m, 1651m, 1454m, 1381m, 1168m; ^{1}H NMR (400 MHz, $CDCl_3$) δ_H 7.58 – 7.52 (2H, m), 7.44 – 7.28 (6H, m), 7.27 – 7.23 (2H, m), 5.49 (1H, br. s), 5.06 –

4.99 (1H, m), 4.41 (2H, d, J = 5.5 Hz), 3.54 (3H, q, J = 1.0 Hz), 2.65 (1H, quin, J = 8.5 Hz), 2.50 (1H, ddd, J = 11.5, 8.5, 2.5 Hz), 2.36 – 2.30 (2H, m), 2.29 – 2.19 (2H, m), 1.94 – 1.83 (2H, m), 1.73 – 1.63 (1H, m); ^{13}C NMR (101 MHz, $CDCl_3$) δ_C 174.3, 166.4, 138.4, 132.1, 129.8, 128.9, 128.7, 128.0, 127.7, 127.6, 123.5 (q, J = 289 Hz), 84.8 (q, J = 28 Hz), 76.7, 55.5, 44.9, 43.8, 35.0 (two peaks), 31.4, 27.0, 25.1; HRMS (ESI+) *m/z* $[M+H]^+$ calcd for $C_{25}H_{27}F_3NO_4$, 462.1887: found: 462.1884.

*S4.24 (2R,4s,6R)-6-(Benzylcarbamoyl)spiro[3.3]heptan-2-yl (S)-3,3-trifluoro-2-methoxy-2-phenylpropanoate (**S3**)*

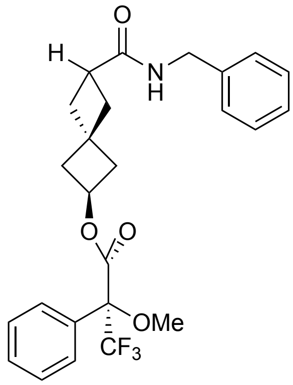

Prepared according to general procedure C from alcohol **3** (2.0 mg, 0.01 mmol) and (*R*)-(−)-MTPA-Cl. Purification by column chromatography (petrol/ethyl acetate, 80:20) gave the title compound as a yellow paste (2.4 mg, 52%). R_f 0.24 (petrol/ethyl acetate, 66:34); $[\alpha]_D^{25}$ –35.2 (*c* 0.3, dichloromethane); IR ν_{max} (thin film)/cm^{-1} 3294 br s, 2978s, 2943s, 1744s, 1647m, 1543m, 1265m, 1169s; ^{1}H NMR (400 MHz, $CDCl_3$) δ_H 7.52 – 7.47 (2H, m), 7.43 – 7.38 (3H, m), 7.36 – 7.28 (3H, m), 7.26 – 7.23 (2H, m), 5.57 (1H, br. s), 5.11 (1H, quin, J = 7.0 Hz), 4.42 (2H, d, J = 5.5 Hz), 3.54 (3H, q, J = 1.0 Hz), 2.89 (1H, quin, J = 8.5 Hz), 2.65 – 2.57 (1H, m), 2.53 – 2.45 (1H, m), 2.43 – 2.33 (2H, m), 2.28 – 2.05 (4H, m); ^{13}C NMR (101 MHz, $CDCl_3$) δ_C 174.1, 166.1, 138.4, 132.3, 129.8, 128.9, 128.6, 128.0, 127.7, 127.4, 123.4 (q, J = 289 Hz), 84.5 (q, J = 28 Hz), 67.6, 55.6, 43.8, 42.5, 41.8, 37.5, 37.2, 35.2, 33.2; HRMS (ESI+) *m/z* $[M+H]^+$ calcd for $C_{25}H_{27}F_3NO_4$, 462.1887: found: 462.1884.

*S4.25 (2R,4s,6R)-6-(Benzylcarbamoyl)spiro[3.3]heptan-2-yl (R)-3,3,3-trifluoro-2-methoxy-2-phenylpropanoate (**S4**)*

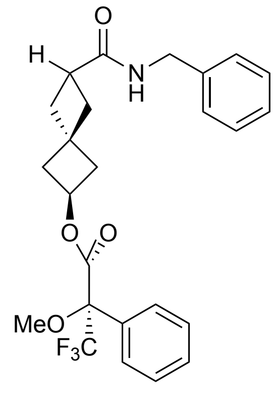

Prepared according to general procedure C from alcohol **3** (4.0 mg, 0.016 mmol) and (*S*)-(+)-MTPA-Cl. Purification by column chromatography (petrol/ethyl acetate, 80:20) gave the title compound as a pale yellow paste (6.4 mg, 87%). R_f 0.24 (petrol/ethyl acetate, 66:34); $[\alpha]_D^{25}$ +44.0 (*c* 0.02, dichloromethane); IR ν_{max} (thin film)/cm^{-1} 3340 br s, 2978s, 2943s, , 1743m, 1701s, 1662s, 1269s, 1122s; ^{1}H NMR (400 MHz, $CDCl_3$) δ_H 7.53 – 7.47 (2H, m), 7.46 – 7.38 (3H, m), 7.36 – 7.27 (3H, m), 7.27 – 7.23 (2H, m), 5.56 (1H, br. s), 5.12 (1H, quin, 7.0 Hz), 4.42 (2H, d, J = 5.5 Hz), 3.53 (3H, q, J = 1.0 Hz), 2.89 (1H, quin, J = 8.5 Hz), 2.63 – 2.56 (1H, m), 2.53 – 2.45 (1H, m), 2.42 – 2.34 (2H, m), 2.27 – 2.20 (1H, m), 2.17 – 2.08 (3H, m); ^{13}C NMR (101 MHz, $CDCl_3$) δ_C 174.3, 166.1, 138.3,

132.3, 129.8, 128.9, 128.6, 128.0, 127.8, 127.4, 67.6, 55.6, 43.8, 42.6, 41.6, 37.5, 37.2, 35.2, 33.2 (CF_3 and **C**CF_3 not resolved); HRMS (ESI+) *m/z* $[M+H]^+$ calcd for $C_{25}H_{27}F_3NO_4$, 462.1887: found: 462.1888.

*S4.26 (1R,4s,6R)-6-(Benzylcarbamoyl)spiro[3.3]heptan-1-yl (S)-3,3,3-trifluoro-2-methoxy-2-phenylpropanoate (**S5**)*

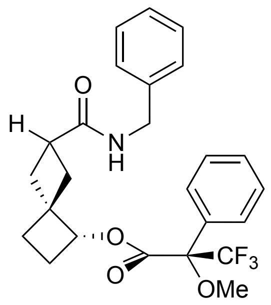

Prepared according to general procedure C from alcohol **5** (10.0 mg, 0.041 mmol) and (*R*)-(−)-MTPA-Cl. Purification by column chromatography (petrol/ethyl acetate, 80:20) gave the title compound as a colorless solid (4.1 mg, 22%). R_f 0.70 (petrol/ethyl acetate, 50:50); $[\alpha]_D^{25}$ −5.4 (*c* 0.4, dichloromethane); IR ν_{max} (thin film)/cm^{-1} 3016br s, 2927s, 2854s, 1760m, 1560m, 1219m; ^{1}H NMR (400 MHz, $CDCl_3$) δ_H 7.60 – 7.53 (2H, m), 7.44 – 7.36 (3H, m), 7.36 – 7.22 (5H, m), 5.62 (1H, br. s), 4.99 (1H, t, *J* = 8.5 Hz), 4.42 (2H, d, *J* = 5.5 Hz), 3.55 (3H, part-resolved q, *J* = 1.0 Hz), 2.93 (1H, quin, *J* = 9.0 Hz), 2.50 – 2.38 (2H, m), 2.36 – 2.28 (1H, m), 2.14 – 2.05 (1H, m), 2.05 – 1.91 (3H, m), 1.81 – 1.75 (1H, m); ^{13}C NMR (101 MHz, $CDCl_3$) δ_C 173.8, 166.3, 138.5, 132.4, 129.8, 128.9, 128.6, 127.9, 127.7, 127.6, 123.5 (q, *J* = 289 Hz), 84.7 (q, *J* = 28 Hz), 74.5, 55.6, 44.3, 43.7, 34.5, 34.3, 32.1, 27.7, 25.1; HRMS (ESI+) *m/z* $[M+H]^+$ calcd for $C_{25}H_{27}F_3NO_4$, 462.1887: found: 462.1887.

*S4.27 (1R,4s,6R)-6-(Benzylcarbamoyl)spiro[3.3]heptan-1-yl (R)-3,3,3-trifluoro-2-methoxy-2-phenylpropanoate (**S6**)*

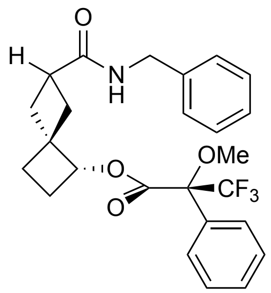

Prepared according to general procedure C from alcohol **5** (10.0 mg, 0.041 mmol) and (*S*)-(+)-MTPA-Cl). Purification by column chromatography (petrol/ethyl acetate, 80:20) gave the title compound as a colorless solid (10.0 mg, 53%). R_f 0.70 (petrol/ethyl acetate, 50:50); $[\alpha]_D^{25}$ +15.0 (*c* 0.2, dichloromethane); IR ν_{max} (thin film)/cm^{-1} 3012s, 2974s, 2936s, 1747m, 1539m, 1523m, 1454m, 1219m; ^{1}H NMR (400 MHz, $CDCl_3$) δ_H 7.57 – 7.52 (2H, m), 7.44 – 7.38 (3H, m), 7.34 – 7.27 (3H, m), 7.26 – 7.21 (2H, m), 5.62 (1H, br. s), 4.96 (1H, t, *J* = 7.5 Hz), 4.40 (2H, d, *J* = 5.5 Hz), 3.56 (3H, s), 2.94 (1H, quin, *J* = 9.0 Hz), 2.53 (1H, dd, *J* = 11.5, 9.0 Hz), 2.47 (1H, dd, *J* = 11.5, 9.0 Hz), 2.36 – 2.28 (1H, m), 2.17 – 1.96 (3H, m), 1.92 – 1.82 (1H, m), 1.81 – 1.72 (1H, m); ^{13}C NMR (101 MHz, $CDCl_3$) δ_C 174.2, 166.6, 138.8, 132.5, 130.1, 129.2, 129.0, 128.2, 128.0, 123.8 (q, *J* = 289 Hz), 85.1 (q, *J* = 28 Hz), 75.2, 56.0, 44.3, 44.0, 34.8, 34.7, 32.6, 28.1, 25.7; HRMS (ESI+) *m/z* $[M+H]^+$ calcd for $C_{25}H_{27}F_3NO_4$, 462.1887: found: 462.1884.

S5 Mosher ester analysis

S5.1 Mosher ester analysis for (2S,4r,5R)-N-benzyl-5-hydroxyspiro[3.3]heptane-2-carboxamide **2**

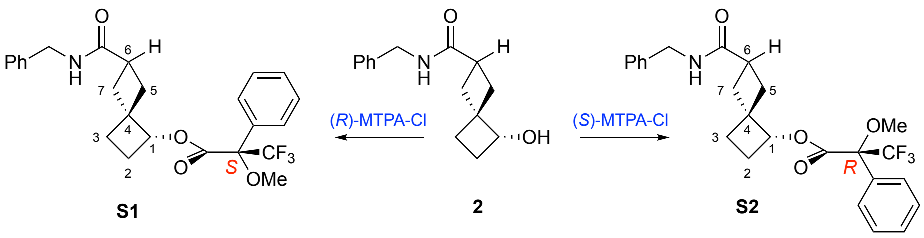

Table S5.1.1. $\Delta\delta^{SR}$ data for the (*S*)- and (*R*)- Mosher's esters, **2**-(*S*)-**S1** and **2**-(*R*)-**S2**. The alcohol **2** was isolated from a reaction with variant K19/F87I/I263G/A330I. δ (*S*)-**S1** and δ (*S*)-**S2** are the proton chemical shifts. $\Delta\delta^{SR}$ (Hz) = 400 × [δ (*S*)-**S1** – δ (*R*)-**S2**]. Predicted $\Delta\delta^{SR}$ is the expected direction of the shift of δ (*S*)-**S1** relative to δ (*R*)-**S2** for each identified proton. The results support the (2*S*,5*R*) configuration for alcohol **2**.

Proton[a]	**δ (*S*)-S1** (ppm)	**δ (*R*)-S2** (ppm)	$\Delta\delta^{SR}$ (Hz)	**Predicted $\Delta\delta^{SR}$** (2*S*,5*R*)-**2**	**Predicted $\Delta\delta^{SR}$** (2*R*,5*S*)-**2**
2_α	2.26	2.28	–8		
2_β	1.93	1.87	+24		
2_{ave}	2.10	2.08	+8	+	–
$5_{trans}/7_{trans}$	2.24	2.33	–36		
$5_{trans}/7_{trans}$	2.15	2.21	–24		
$5/7_{ave}$	2.20	2.27	–28	–	+
6	2.48	2.65	–68	–	+

[a] Designations $2_\alpha/2_\beta$ refer to the 'down' and 'up' protons, respectively; $5_{trans}/7_{trans}$ refer to protons *trans* to CONHBn.

S5.2 Mosher ester analysis for (2R,4s,5R)-N-benzyl-5-hydroxyspiro[3.3]heptane-2-carboxamide **5**

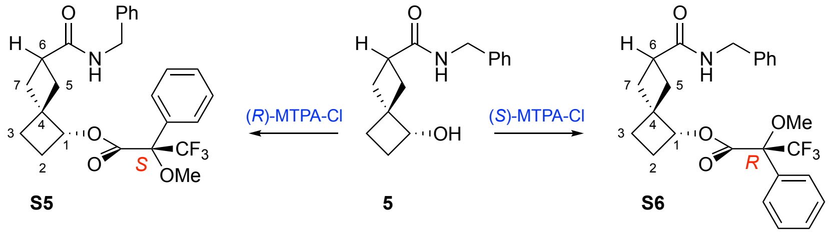

Table S5.2.1. $\Delta\delta^{SR}$ data for the (*S*)- and (*R*)- Mosher's esters, **5**-(*S*)-**S5** and **5**-(*R*)-**S6**. The alcohol **5** was isolated from a reaction with variant R19/F87A/L75F. δ (*S*)-**S5** or (*R*)-**S6** are the proton chemical shifts. $\Delta\delta^{SR}$ = 400 × [δ (*S*)-**S5** – δ (*R*)-**S6**]. Predicted $\Delta\delta^{SR}$ is the expected direction of the shift of the δ (*S*)-**S5** relative to δ (*R*)-**S6** for each identified proton. The results support the (2*R*,5*R*) configuration for alcohol **5** which accords with the crystal structure determination (§ **S6.2**).

Proton[a]	**δ (*S*)-S5** (ppm)	**δ (*R*)-S6** (ppm)	$\Delta\delta^{SR}$ (Hz)	**Predicted $\Delta\delta^{SR}$** (2*R*,5*R*)-**5**	**Predicted $\Delta\delta^{SR}$** (2*S*,5*S*)-**5**
2_α	2.32	2.32	0		
2_β	1.97	1.87	+40		
2_{ave}	2.15	2.10	+20	+	–
$5_{cis}/7_{cis}$	2.47	2.53	–24		
$5_{cis}/7_{cis}$	2.42	2.46	–16		
$5/7_{ave}$	2.45	2.50	–20	–	+
6	2.93	2.94	–4	(–)	(+)

[a] Designations $2_\alpha/2_\beta$ refer to the 'down' and 'up' protons, respectively; $5_{cis}/7_{cis}$ refer to protons *cis* to CONHBn.

S6 Crystallographic details

Data were acquired by Hangzhou Yanqu Information Technology Co., Ltd (China) using a Rigaku XtaLAB P200 diffractometer. A multilayer mirror was employed as the monochromator to focus the beam, and a collimator with a diameter of 0.3 mm was used. The instrument operated at a voltage of 40 kV and a current of 30 mA.

*S6.1 Crystallographic details for azidoformate **11***

Single crystals were grown by vapor diffusion of petroleum ether into a chloroform solution of azidoformate **11** (vial in vial method). Full refinement details are given in the CIF available from the Cambridge Crystallographic Data Centre (CCDC 2424351) via www. ccdc.cam.ac.uk/data_request/cif.

CCDC code	2424351	
Empirical formula	$C_{16}H_{18}N_4O_3$	
Formula weight	314.34	
Temperature	113.15 K	
Wavelength	1.54184 Å	
Crystal system / Spacegroup	Monoclinic	$P2_1$
Unit cell dimensions	a = 12.6700(2) Å	α = 90°
	b = 9.28740(10) Å	β = 98.2930(10)°
	c = 13.8244(2) Å	γ = 90°
Volume	1609.73(4) $Å^3$	
Z	4	
Density (calculated)	1.297 Mg/m^3	
Crystal size	0.25 × 0.22 × 0.20 mm^3	
Independent reflections	5636 [R(int) = 0.0361]	
Completeness to theta = 67.684°	99.5 %	
Absorption correction	Multi-scan	
Refinement method	Full-matrix least-squares on F^2	
Data / restraints / parameters	5636 / 1 / 415	
Goodness-of-fit on F^2	1.067	
Final R indices [I>2sigma(I)]	R1 = 0.0450, wR2 = 0.1249	
R indices (all data)	R1 = 0.0465, wR2 = 0.1271	
Absolute structure (Flack x) parameter	0.12(12)	

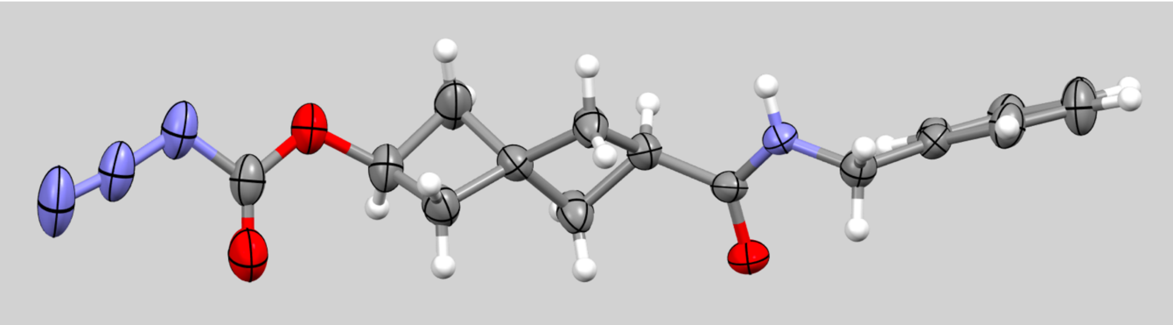

(thermal ellipsoids drawn at the 50% probability level)

S6.2 Crystallographic details for azidoformate ***12***

Single crystals were grown by vapor diffusion of petroleum ether into a chloroform solution of azidoformate **12** (vial in vial method). Full refinement details are given in the CIF available from the Cambridge Crystallographic Data Centre (CCDC 2424349) via www. ccdc.cam.ac.uk/data_request/cif.

CCDC code	2424349	
Empirical formula	$C_{16}H_{18}N_4O_3$	
Formula weight	314.34	
Temperature	113.15 K	
Wavelength	1.54184 Å	
Crystal system / Spacegroup	Triclinic	P1
Unit cell dimensions	a = 9.44560(10) Å	α = 91.9470(10)°
	b = 9.62360(10) Å	β = 101.0690(10)°
	c = 18.1093(2) Å	γ = 96.4430(10)°
Volume	1602.72(3) Å^3	
Z	4	
Density (calculated)	1.303 Mg/m^3	
Crystal size	0.25 × 0.22 × 0.20 mm^3	
Independent reflections	12940 [R(int) = 0.0336]	
Completeness to theta = 67.684°	98.4 %	
Absorption correction	Multi-scan	
Refinement method	Full-matrix least-squares on F^2	
Data / restraints / parameters	12940 / 6 / 842	
Goodness-of-fit on F^2	1.056	
Final R indices [I>2sigma(I)]	R1 = 0.0310, wR2 = 0.0849	
R indices (all data)	R1 = 0.0315, wR2 = 0.0854	
Absolute structure (Flack x) parameter	0.06(6)	

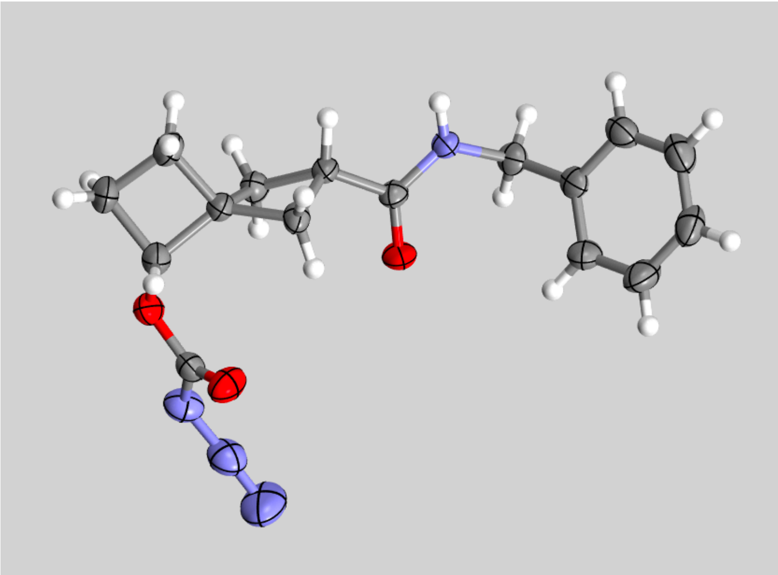

(thermal ellipsoids drawn at the 50% probability level)

S7 Molecular docking analysis and mutation design

*S7.1 Docking of substrate **1** into the MD-simulated structure of variant K19/F87V/I263G to design mutation to improve selectivity for trans alcohol **2**.*

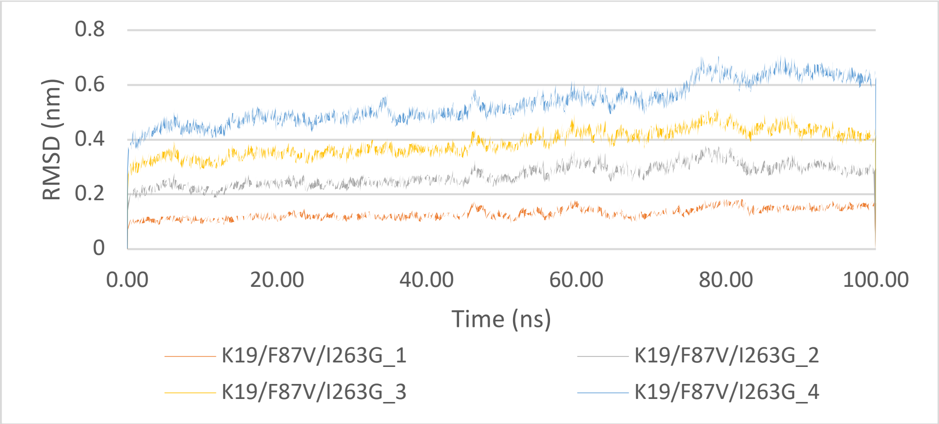

a

Replica	Cluster (population)	Replica	Cluster (population)
R1	C1 (7266)	R3	C1 (9112)
	C2 (1329)		C2 (624)
	C3 (833)		C3 (214)
R2	C1 (4892)	R4	C1 (6177)
	C2 (2149)		C2 (2214)
	C3 (1147)		C3 (666)

b

Figure S7.1.1. MD-simulation outputs for K19/F87V/I263G: **a.** The RMSD plot for Cα atoms of four replicas of K19/F87V/I263G during 100 ns MD simulation runs; **b.** Size of clusters from a total of 10,000 snapshot structures in each replica simulation, with a cluster size cut-off of 5%.

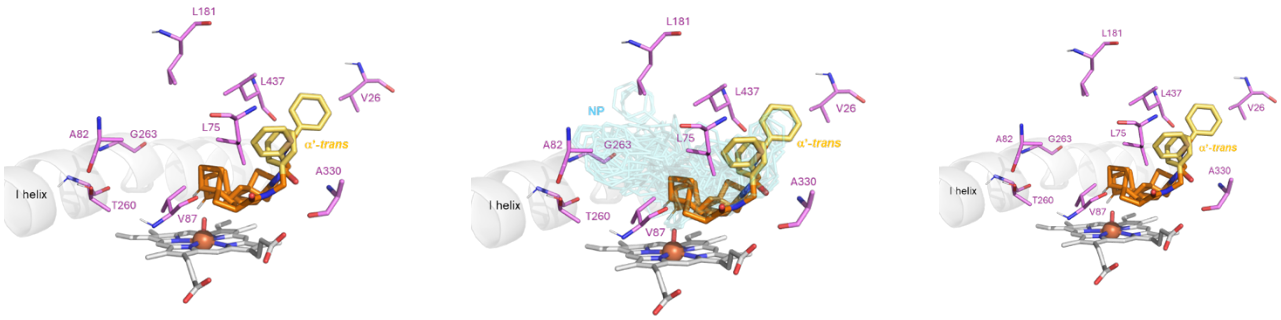

Figure S7.1.2. Analysis of results from the docking of *N*-benzylspiro[3.3]heptane-2-carboxamide **1**, with the K19/F87V/I263G variant. **a.** The simulated poses of the *trans* alcohol **2** (orange) and their positions in the active site. **b.** The *trans* alcohol **2** (orange) poses overlaid with the non-productive (NP) poses (cyan); the NP poses one the left-hand side could be suppressed with enlarged A82 and L75 mutations without affecting the desired *trans* poses; V87I could release more spaces for the formation of *trans* alcohol **2** (orange) poses; A330F mutation might promote van der waals interactions between the phenyl groups on the protecting group and the phenylalanine. **c.** The *trans* alcohol **2** (orange) poses overlaid with the β' alcohol (cyan) poses; the β' alcohol (cyan) poses can be suppressed with the NP poses.

S7.2 *Docking of substrate **1** into the MD-simulated structure of variant K19/F87V/I263G/A330F to design mutation to improve selectivity for trans alcohol **2**.*

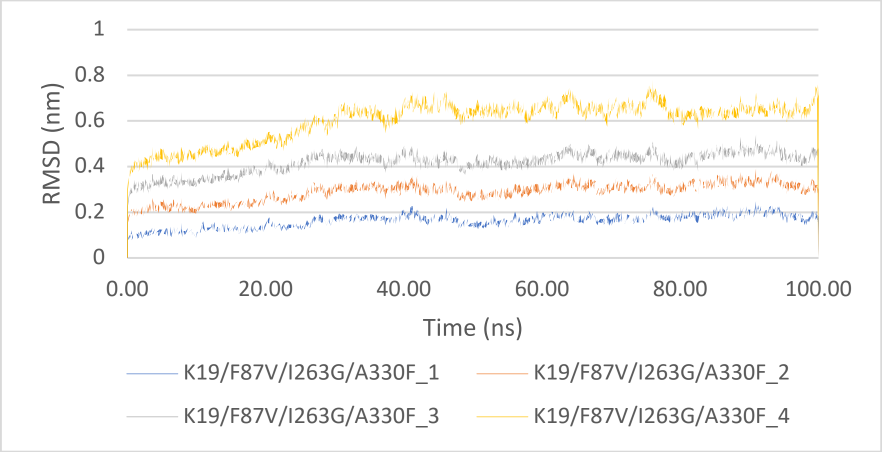

a

Replica	Cluster (population)	Replica	Cluster (population)
R1	C1 (4170)	R3	C1 (8069)
	C2 (2824)		C2 (926)
	C3 (1580)		C3 (520)
R2	C1 (8644)	R4	C1 (2851)
	C2 (915)		C2 (2702)
	C3 (194)		C3 (2048)

b

Figure S7.2.1. MD-simulation outputs for K19/F87V/I263G/A330F: **a.** The RMSD plot for Cα atoms of four replicas of K19/F87V/I263G/A330F during 100 ns MD simulation runs; **b.** Size of clusters from a total of 10,000 snapshot structures in each replica simulation, with a cluster size cut-off of 5%.

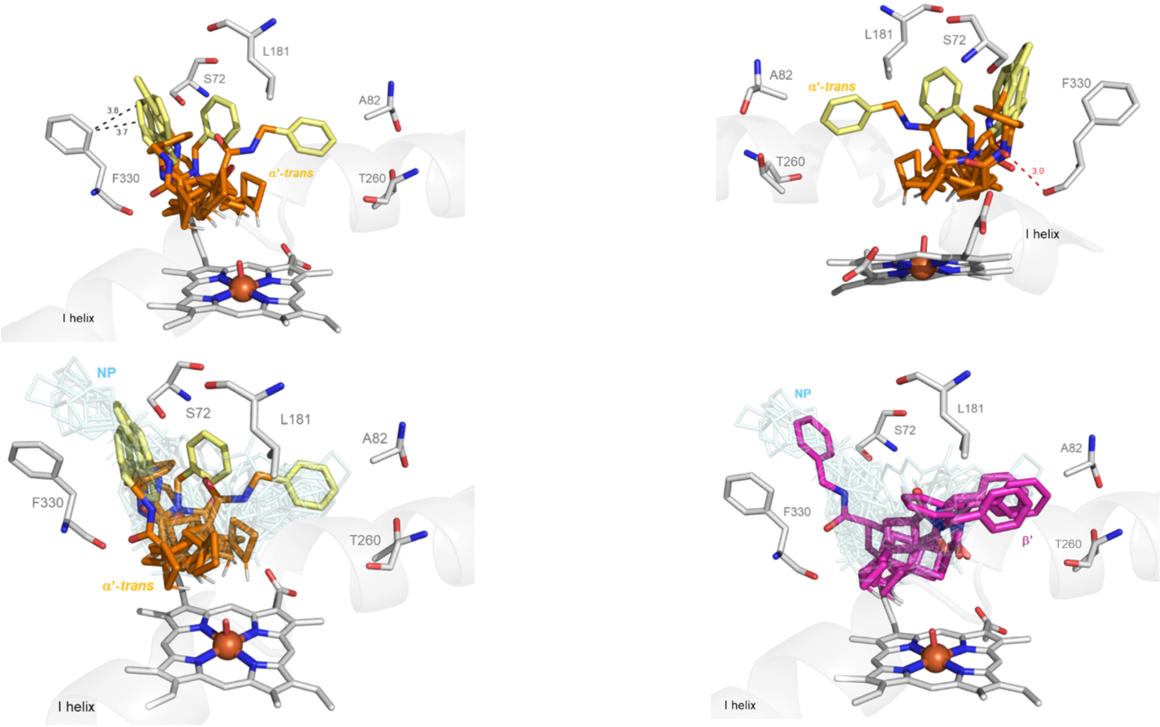

Figure S7.2.2. Analysis of results from the docking of *N*-benzylspiro[3.3]heptane-2-carboxamide **1**, with the K19/F87V/I263G/A330F variant. **a.** The simulated poses of the *trans* alcohol **2** (orange) and their positions in the active site; van der waals interactions of the phenyl group on phenylalanine in A330F residue with the Bn-protecting group as predicted in **S7.1**,

promoting more *trans* alcohol **2** poses compared to its parent variant K19/F87V/I263G. **b.** potential stabilization from the A330F. **c.** The *trans* alcohol **2** poses overlaid with the non-productive (NP) poses (cyan); the NP poses are generally overlapped with the *trans* alcohol **2** poses; mutation on the left-hand side could interfere with the *trans* poses while enlarged L181 and A82 residues might improve *trans* alcohol **2** poses. **d.** The *trans* alcohol **2** poses overlaid with the β' alcohol (purple) poses; the β' alcohol poses overlap with the NP poses; suppressing the NP poses could also decrease the poses of β' alcohol to improve regioselectivity.

*S7.3 Docking of substrate **1** into the MD-simulated structure of variant K19/F87V/I263G/A330I to design mutation to improve selectivity for trans alcohol **2**.*

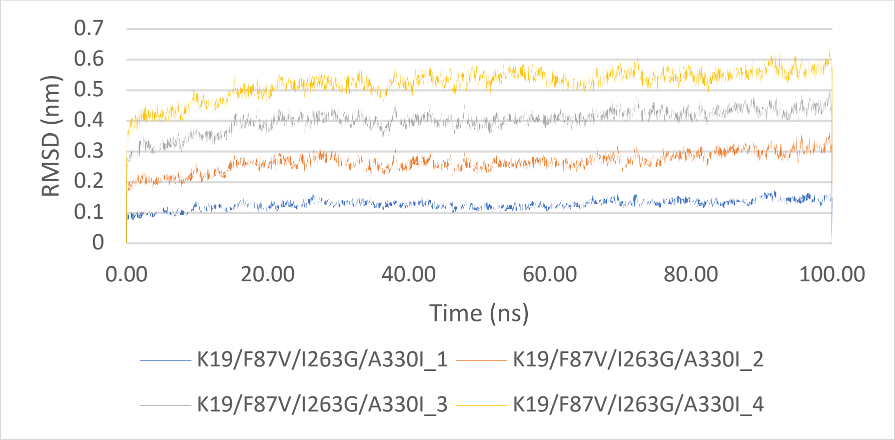

a

Replica	Cluster (population)	Replica	Cluster (population)
R1	C1 (7920)	R3	C1 (6250)
	C2 (1524)		C2 (1916)
	C3 (281)		C3 (1037)
R2	C1 (7187)	R4	C1 (8562)
	C2 (1608)		C2 (540)
	C3 (362)		C3 (464)

b

Figure S7.3.1. MD-simulation outputs for K19/F87V/I263G/A330I: **a.** The RMSD plot for Cα atoms of four replicas of K19/F87V/I263G/A330I during 100 ns MD simulation runs; **b.** Size of clusters from a total of 10,000 snapshot structures in each replica simulation, with a cluster size cut-off of 5%.

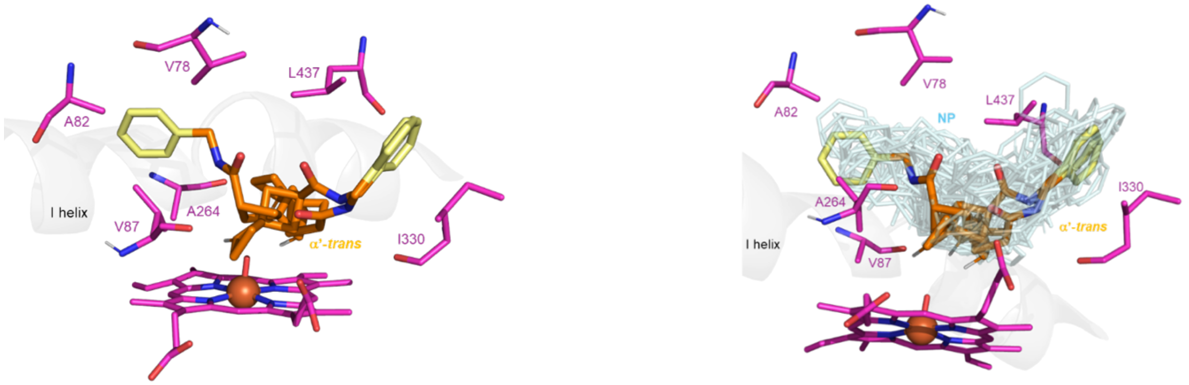

Figure S7.3.2. Analysis of results from the docking of *N*-benzylspiro[3.3]heptane-2-carboxamide **1**, with the K19/F87V/I263G/A330I variant. **a.** The simulated poses of the *trans* alcohol **2** (orange) and their positions in the active site; compared to its parent variant K19/F87V/I263G, poses in the A82, V78 and V87 pocket was promoted; mutant V87I could release more space for stabilizing *trans* alcohol **2** in this pocket. **b.** The *trans* alcohol **2** poses overlaid with the non-productive (NP) poses (cyan); the NP poses are generally overlapped with the *trans* alcohol **2** poses; enlarged L181 and A82 residues could destabilize the NP poses but might interfere with *trans* alcohol **2** poses.

*S7.4 Docking of substrate **1** into the MD-simulated structure of variant K19/F87V/I263G/A330F/A82W to design mutation to improve selectivity for trans alcohol **2**.*

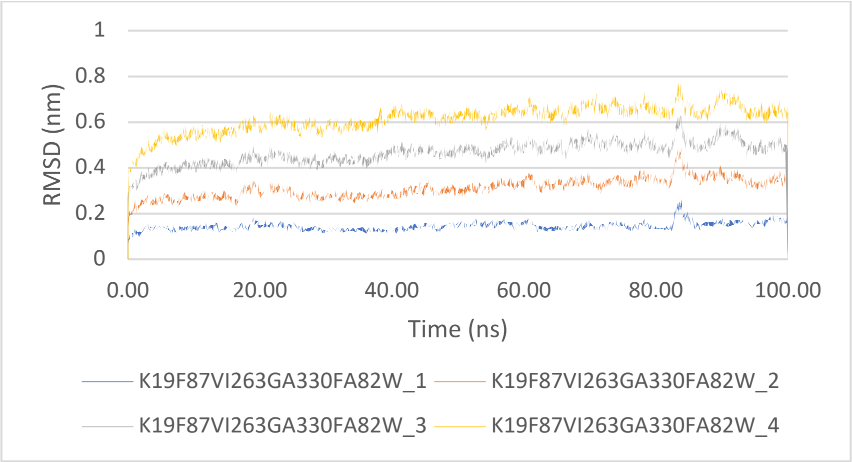

a

Replica	Cluster (population)	Replica	Cluster (population)
R1	C1 (8009)	R3	C1 (5087)
	C2 (1013)		C2 (1654)
	C3 (302)		C3 (1098)
R2	C1 (5870)	R4	C1 (9038)
	C2 (2171)		C2 (504)
	C3 (1273)		C3 (293)

b

Figure S7.4.1. MD-simulation outputs for K19/F87V/I263G/A330F/A83W: **a.** The RMSD plot for Cα atoms of four replicas of K19/F87V/I263G/ A330F/A83W during 100 ns MD simulation runs; **b.** Size of clusters from a total of 10,000 snapshot structures in each replica simulation, with a cluster size cut-off of 5%.

*S7.5 Docking of substrate **1** into the MD-simulated structure of variant GVQ/I263G to design mutation to improve selectivity for β' alcohol **3**.*

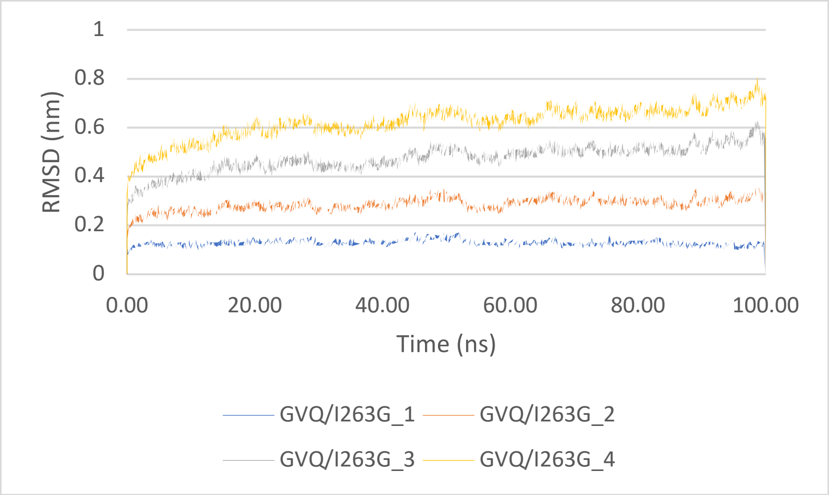

a

Replica	Cluster (population)	Replica	Cluster (population)
R1	C1 (9473)	R3	C1 (5837)
	C2 (308)		C2 (1847)
	C3 (146)		C3 (988)
R2	C1 (6314)	R4	C1 (6136)
	C2 (1677)		C2 (1708)
	C3 (1183)		C3 (1465)

b

Figure S7.5.1. MD-simulation outputs for GVQ/I263G: **a.** The RMSD plot for Cα atoms of four replicas of GVQ/I263G during 100 ns MD simulation runs; **b.** Size of clusters from a total of 10,000 snapshot structures in each replica simulation, with a cluster size cut-off of 5%.

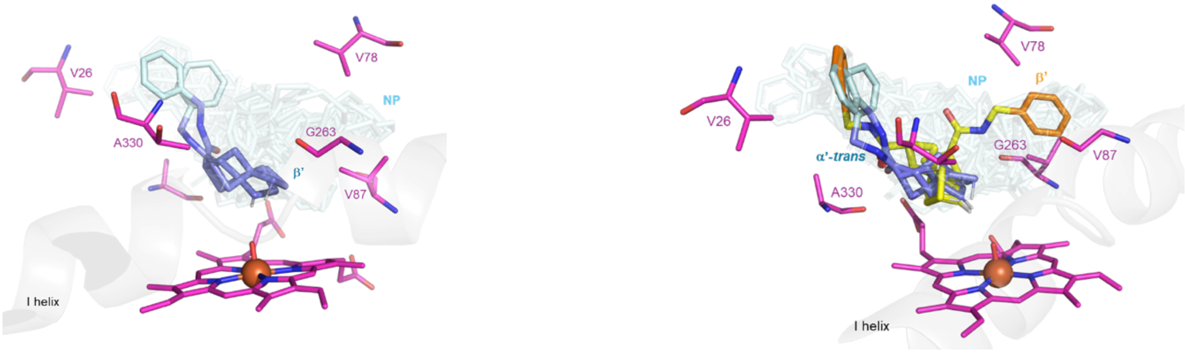

Figure S7.5.2. Analysis of results from the docking of *N*-benzylspiro[3.3]heptane-2-carboxamide **1**, with the GVQ/I263G variant. **a.** The simulated poses of the β' alcohol **3** (blue) and their positions in the active site; The β' alcohol **3** poses overlaid with the non-productive (NP) poses (cyan); A330F mutation might promote van der waals interactions between the phenyl groups on the protecting group and the phenylalanine. **b.** β' alcohol **3** poses overlaid with the non-productive (NP) poses (cyan) and the *trans* alcohol **2** poses; the NP poses are generally overlapped with the *trans* alcohol **2** poses, suppressing the NP poses might improve the regioselectivity towards β' alcohol **3** by destabilizing *trans* alcohol **2** poses.

*S7.6 Docking of substrate **1** into the MD-simulated structure of variant R19/F87A to design mutation to improve selectivity for cis alcohol **5**.*

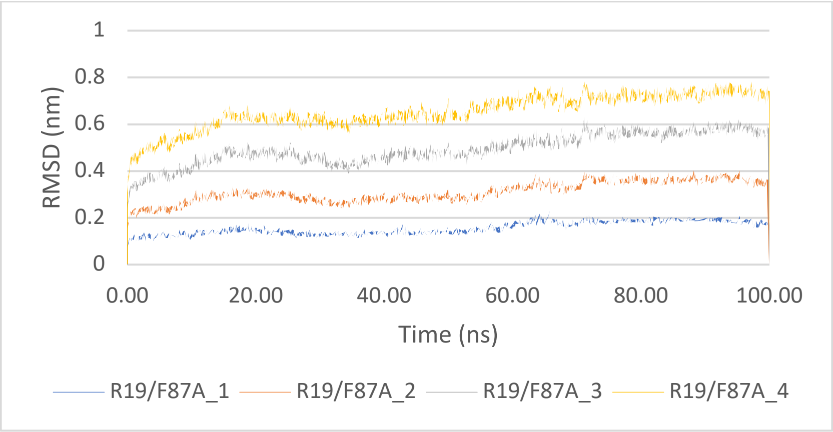

a

Replica	Cluster (population)	Replica	Cluster (population)
R1	C1 (5162)	R3	C1 (3792)
	C2 (3050)		C2 (1837)
	C3 (758)		C3 (1581)
R2	C1 (5918)	R4	C1 (5823)
	C2 (2822)		C2 (2179)
	C3 (709)		C3 (760)

b

Figure S7.6.1. MD-simulation outputs for R19/F87A: **a.** The RMSD plot for Cα atoms of four replicas of R19/F87A during 100 ns MD simulation runs; **b.** Size of clusters from a total of 10,000 snapshot structures in each replica simulation, with a cluster size cut-off of 5%.

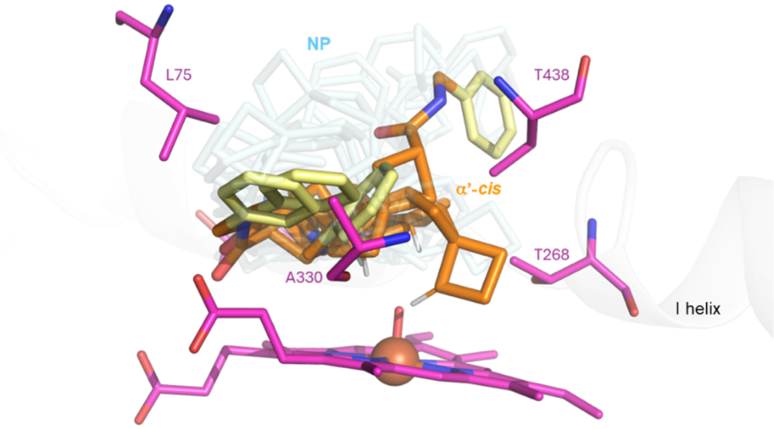

Figure S7.6.2. Analysis of results from the docking of *N*-benzylspiro[3.3]heptane-2-carboxamide **1**, with the R19/F87A variant. The simulated poses of the *cis* alcohol **5** (orange) and their positions in the active site and poses overlaid with the non-productive (NP) poses (cyan). The desired poses sit in a pocket formed from L75, T438, T268 and A330. The NP poses sit in the same pocket, therefore destabilizing the NP poses could also disfavor the productive poses. A small pocket near L75 possesses only NP poses, enlarging the L75 residue could to some extend suppress the NP poses.

^{1}H NMR (400 MHz, CDCl$_3$) (1)

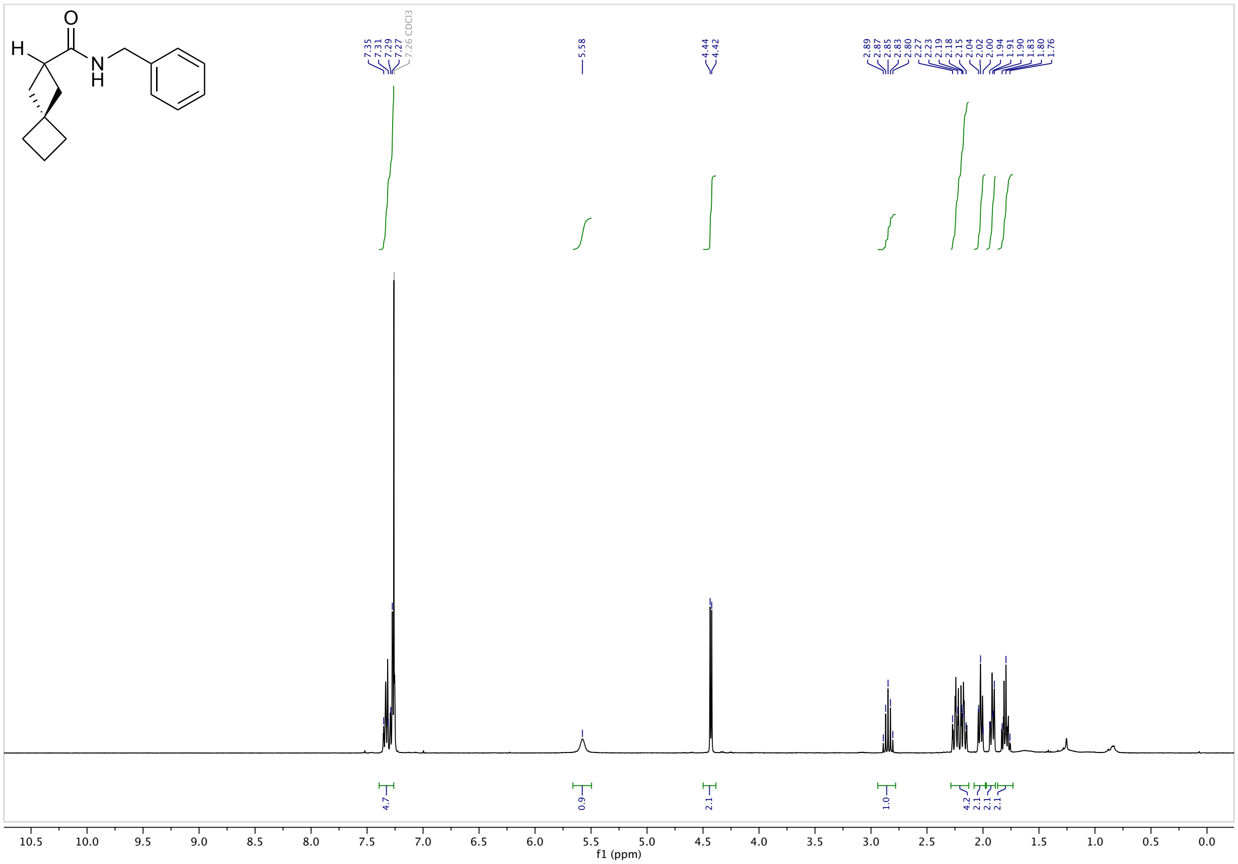

^{13}C NMR (101 MHz, CDCl$_3$) (1)

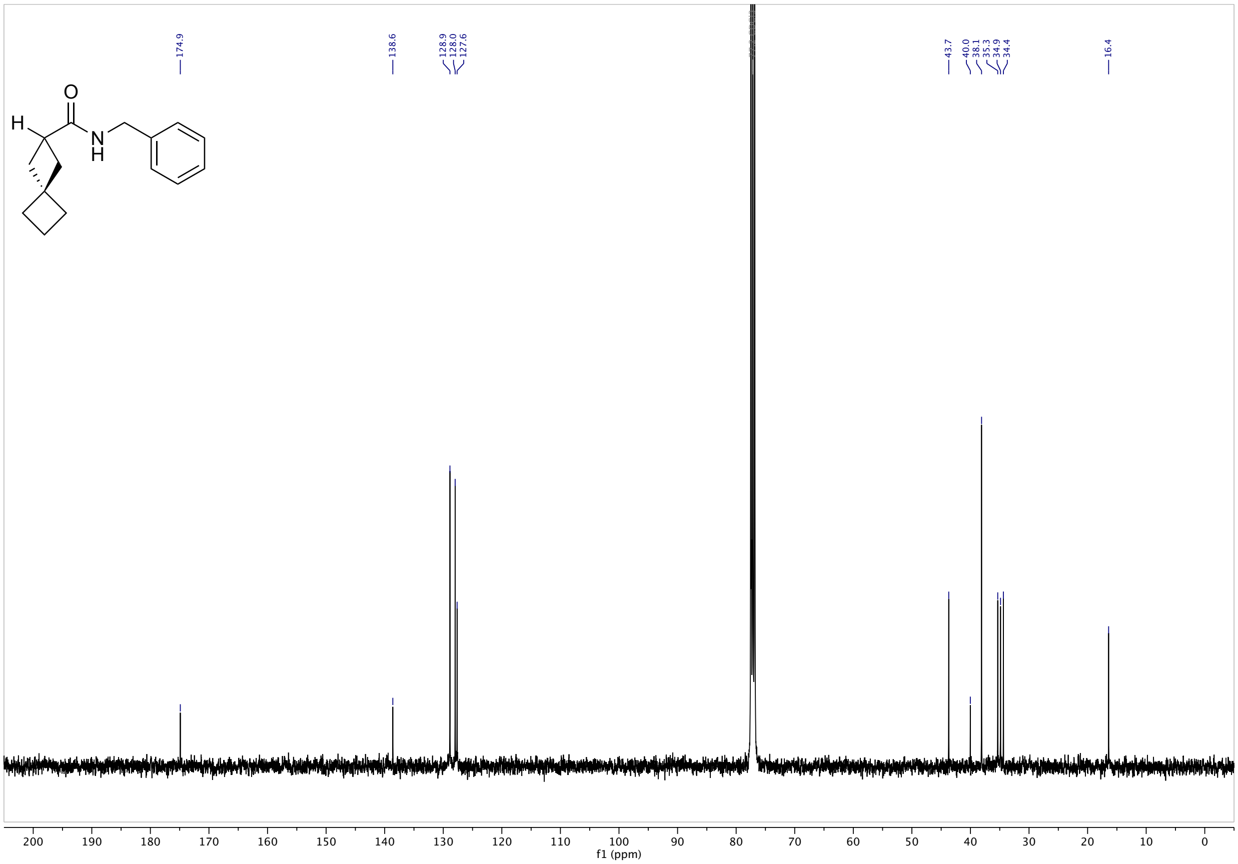

^{1}H NMR (400 MHz, $CDCl_3$) (2)

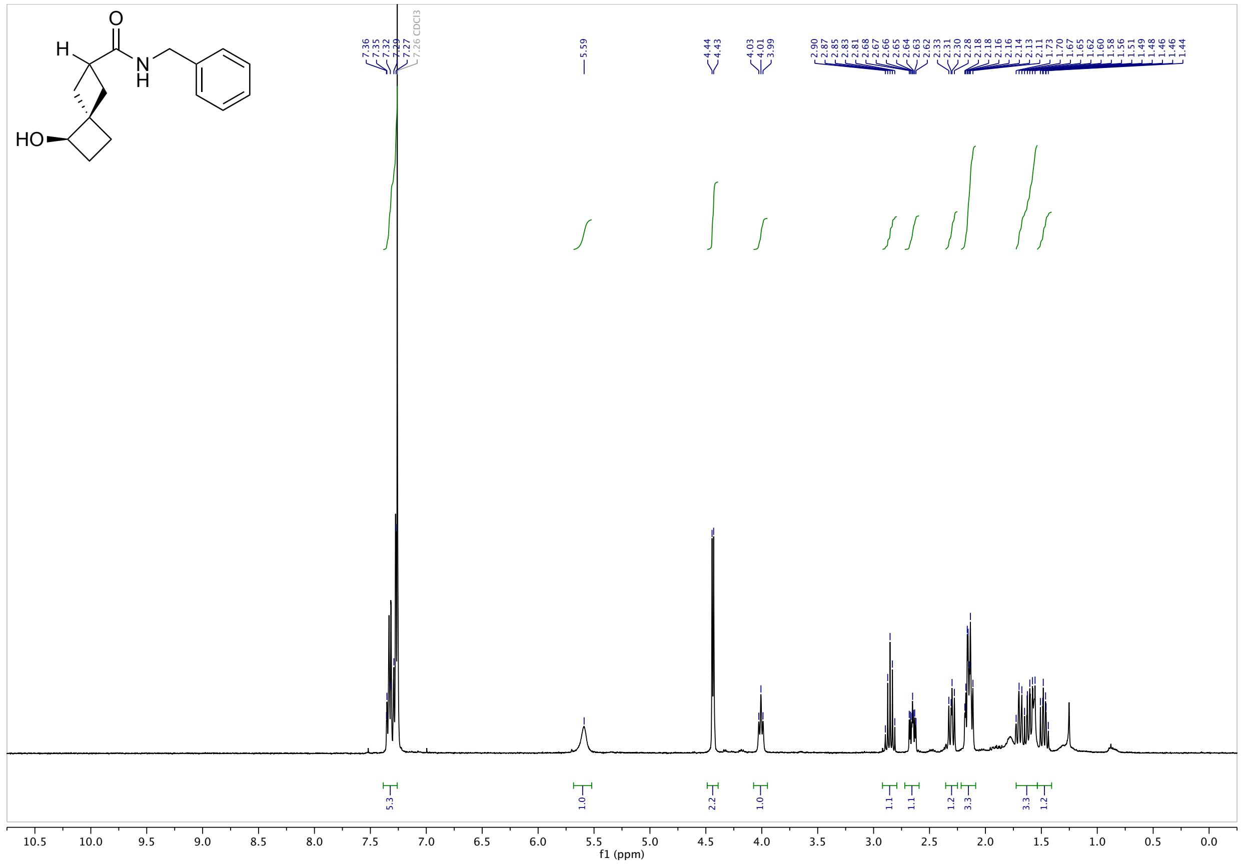

^{13}C NMR (101 MHz, $CDCl_3$) (2)

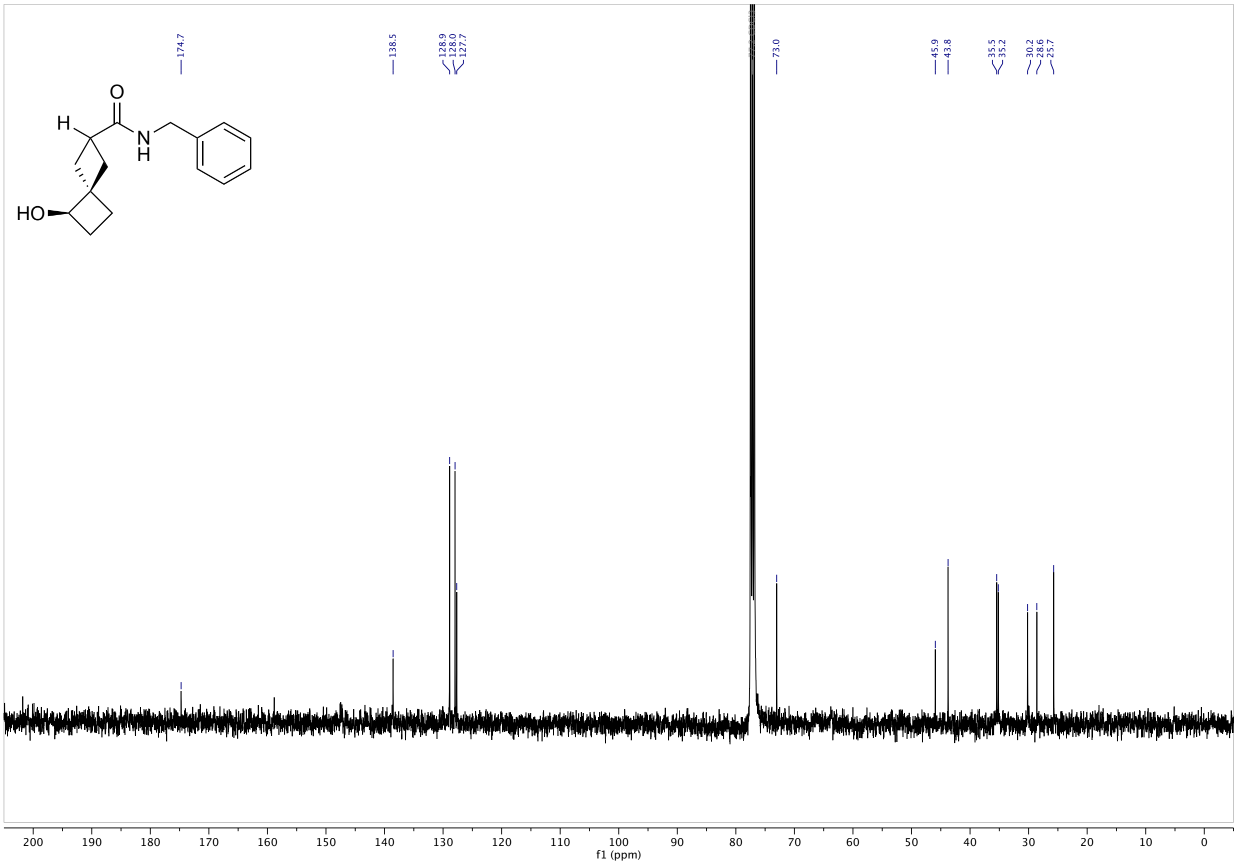

^{1}H NMR (400 MHz, CDCl$_3$) (3)

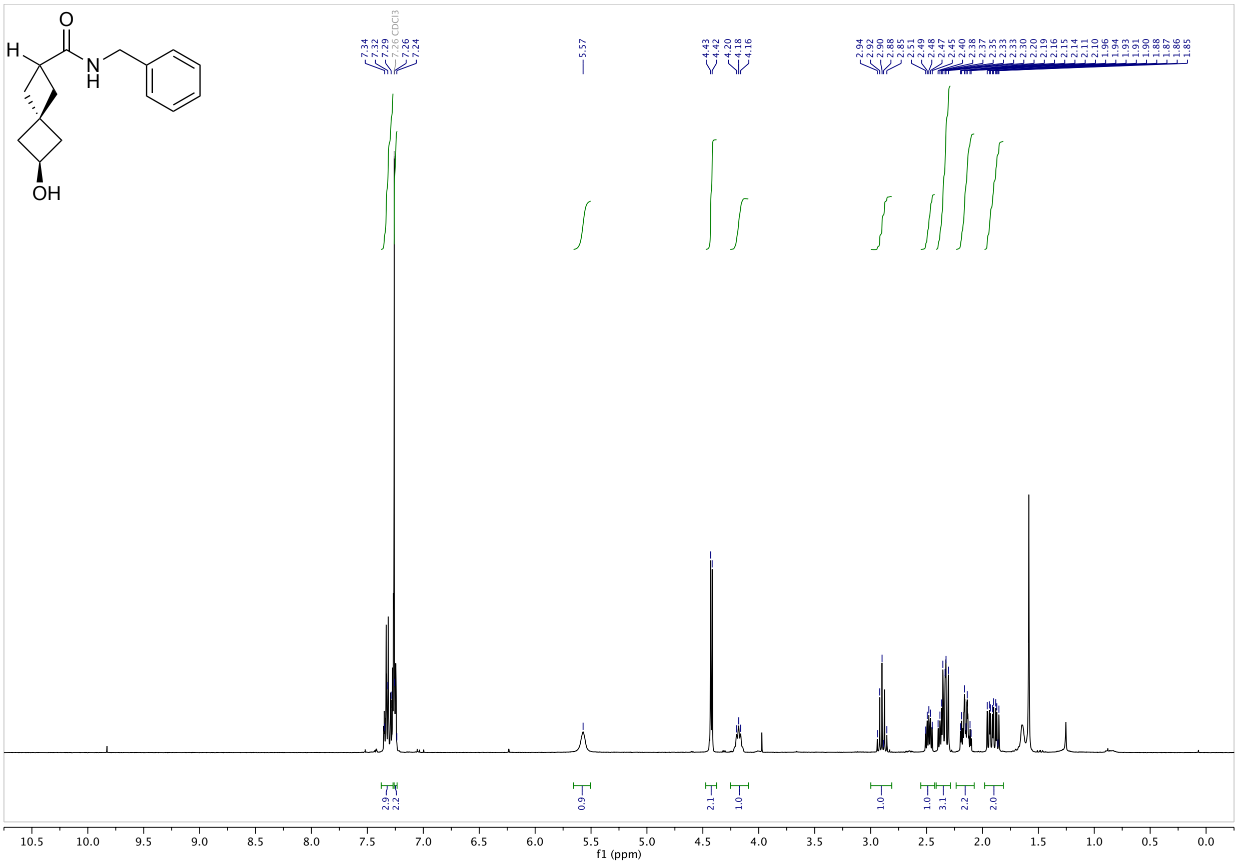

^{13}C NMR (101 MHz, CDCl$_3$) (3)

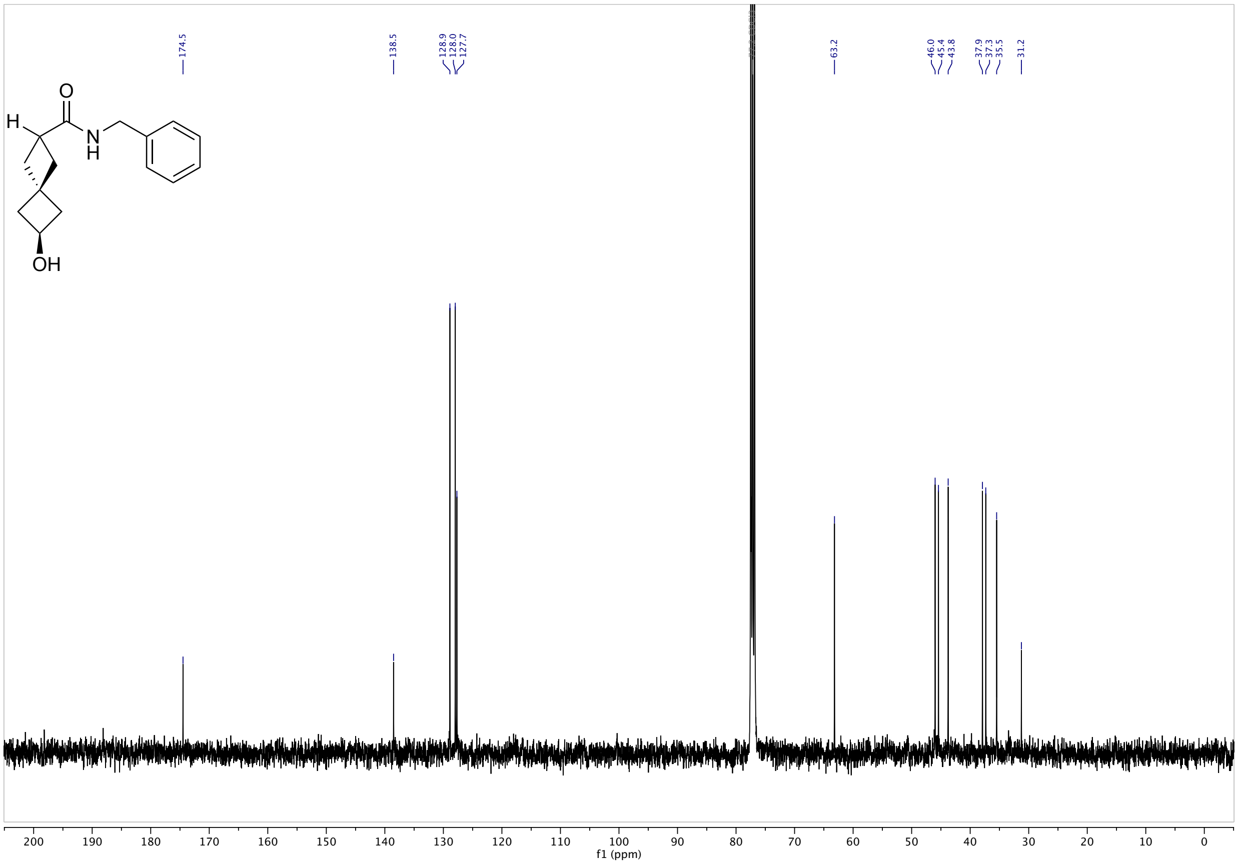

^{1}H NMR (400 MHz, CDCl$_3$) (4)

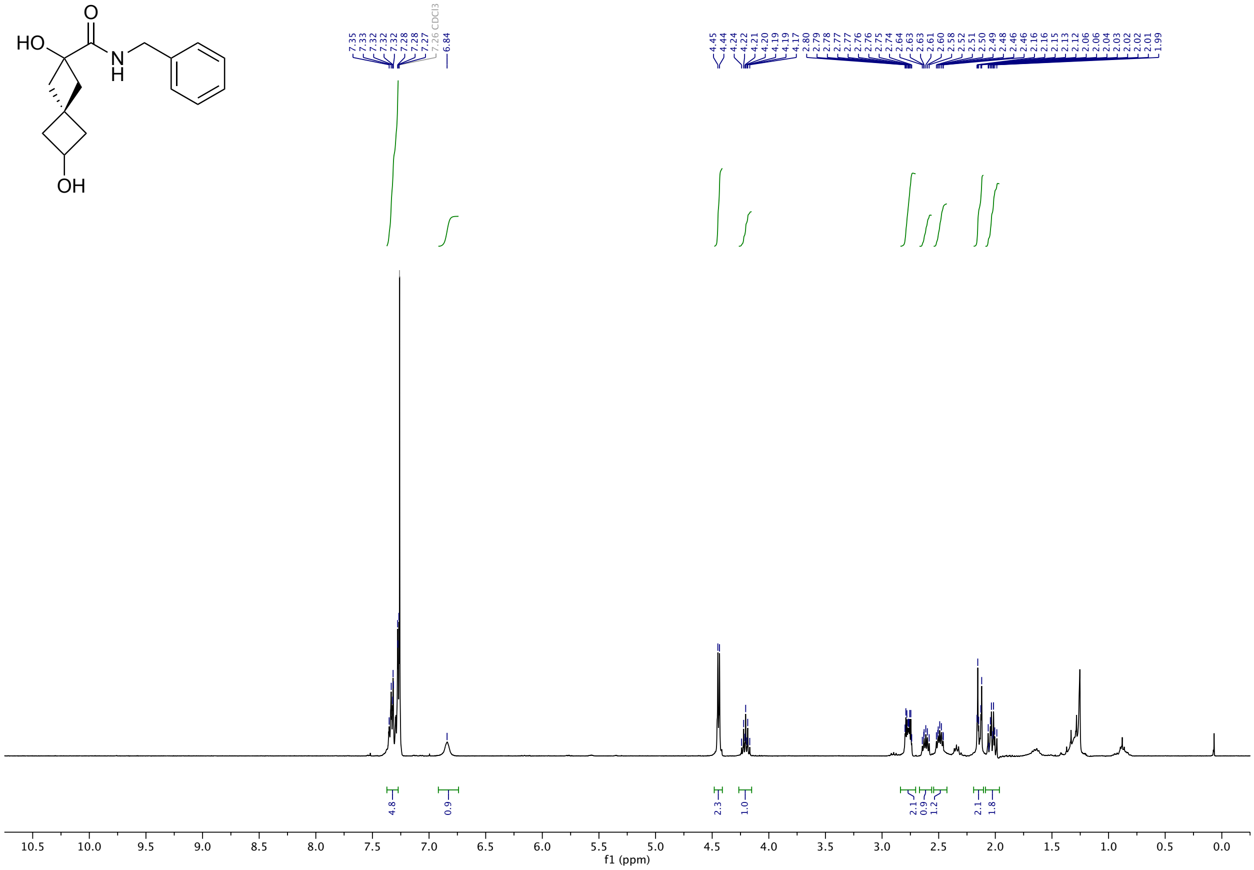

^{13}C NMR (101 MHz, CDCl$_3$) (4)

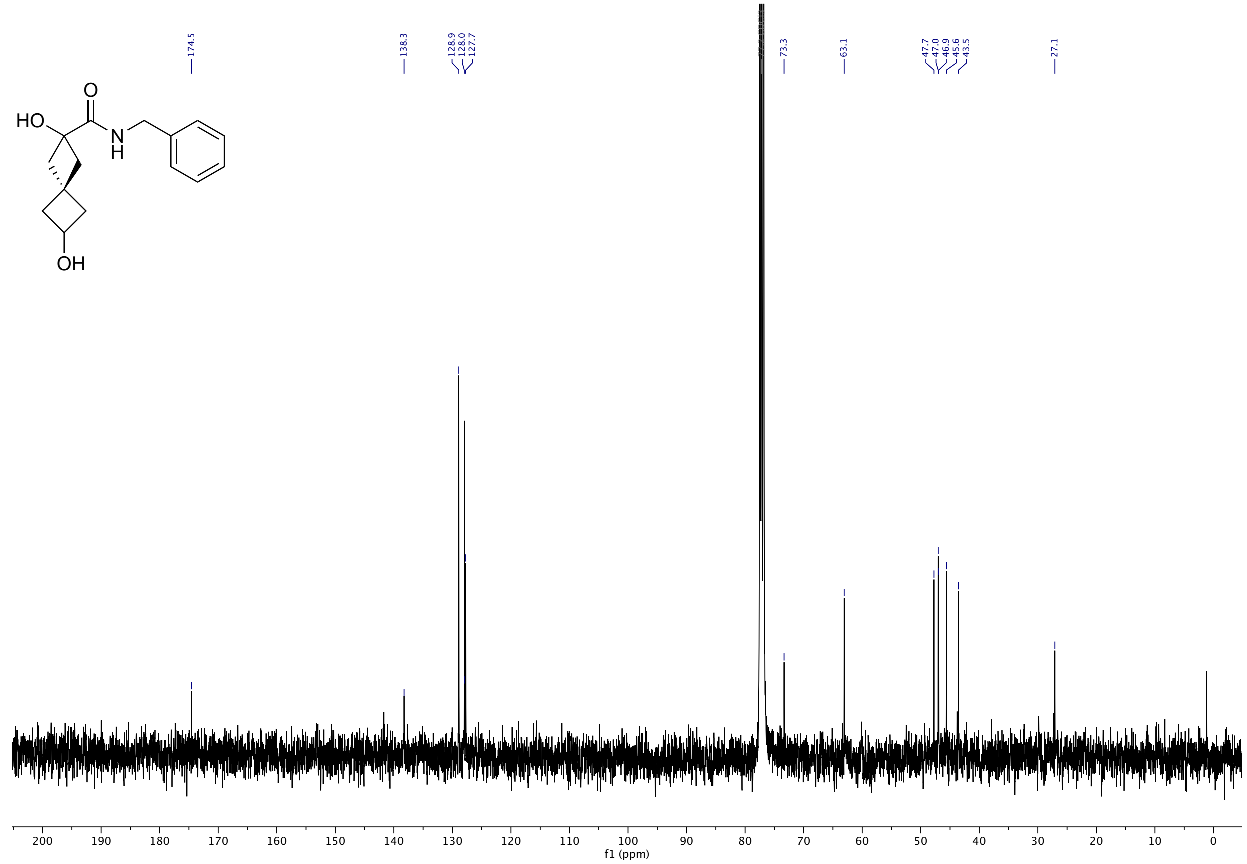

^{1}H NMR (400 MHz, $CDCl_3$) (5)

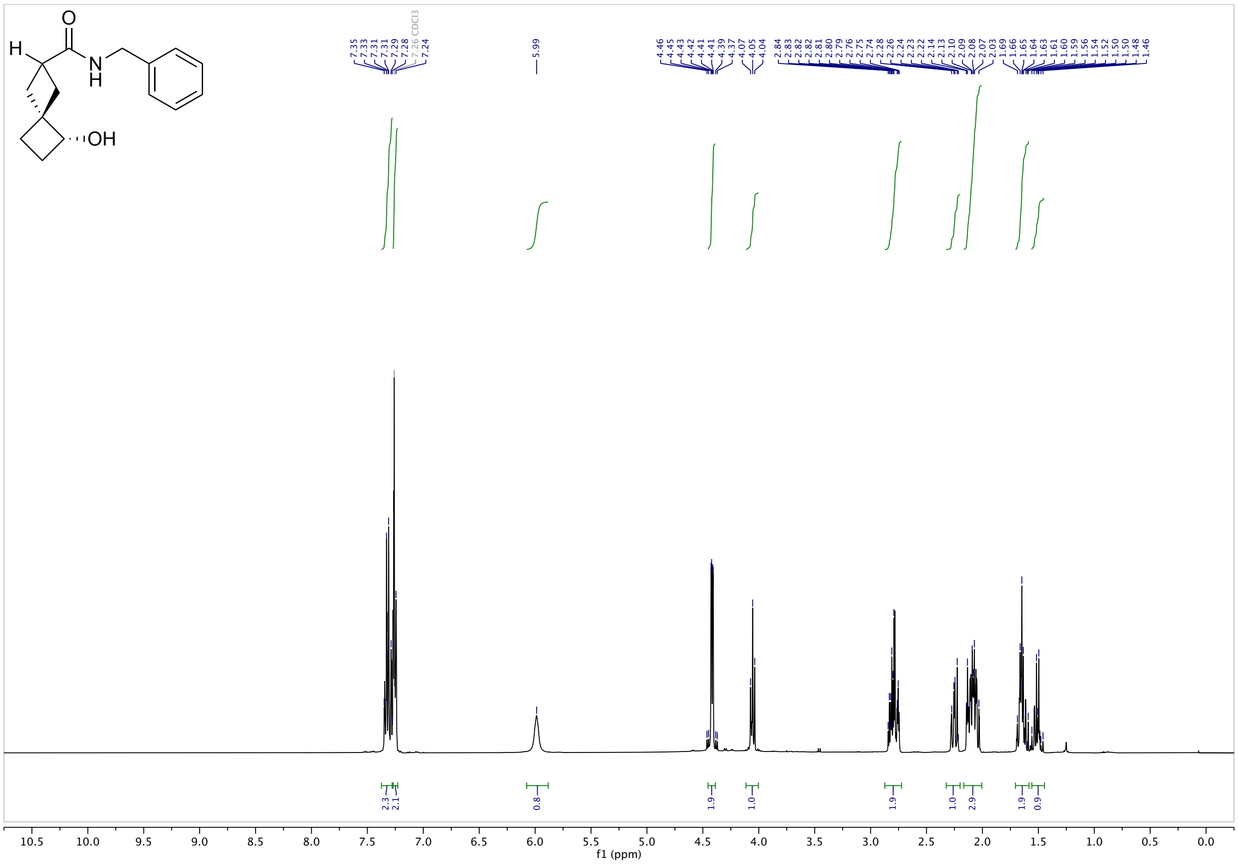

^{13}C NMR (101 MHz, $CDCl_3$) (5)

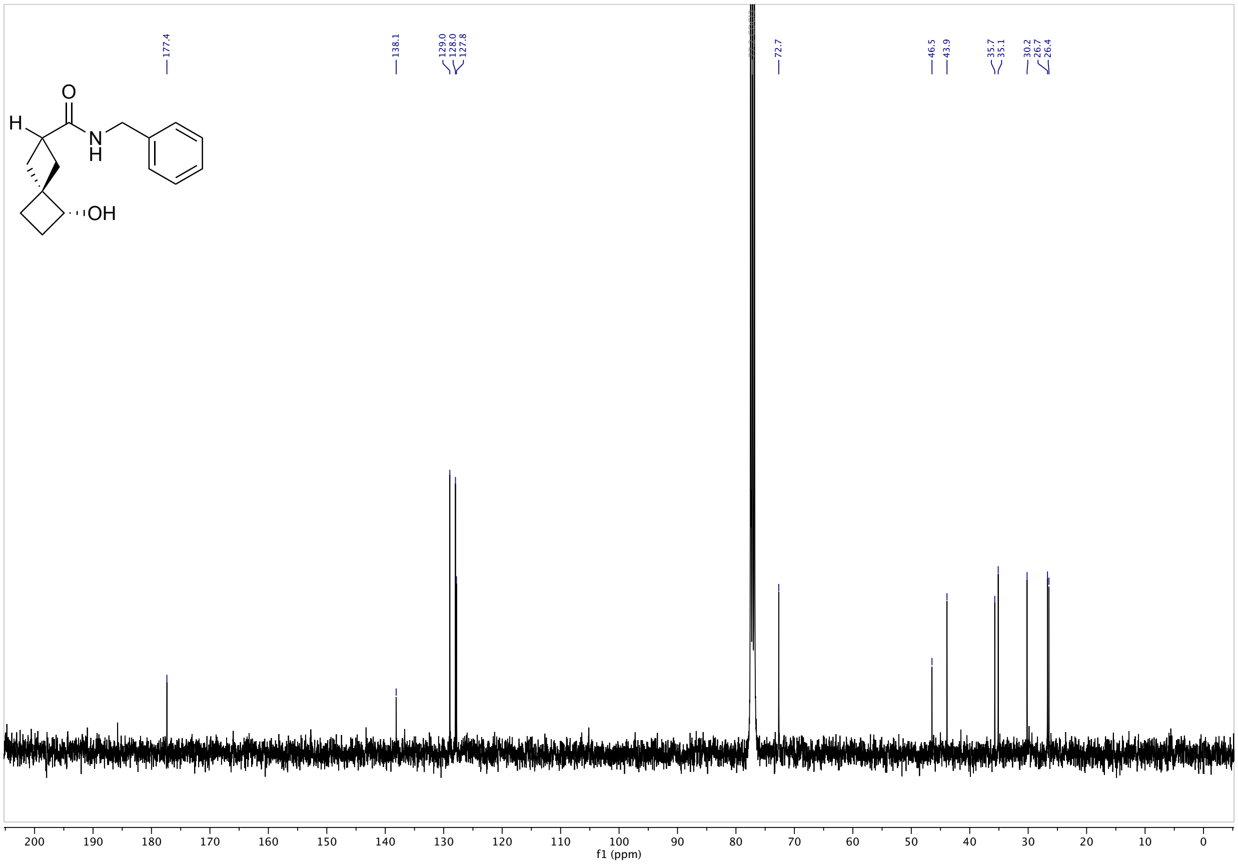

^{1}H NMR (400 MHz, $CDCl_3$) (6)

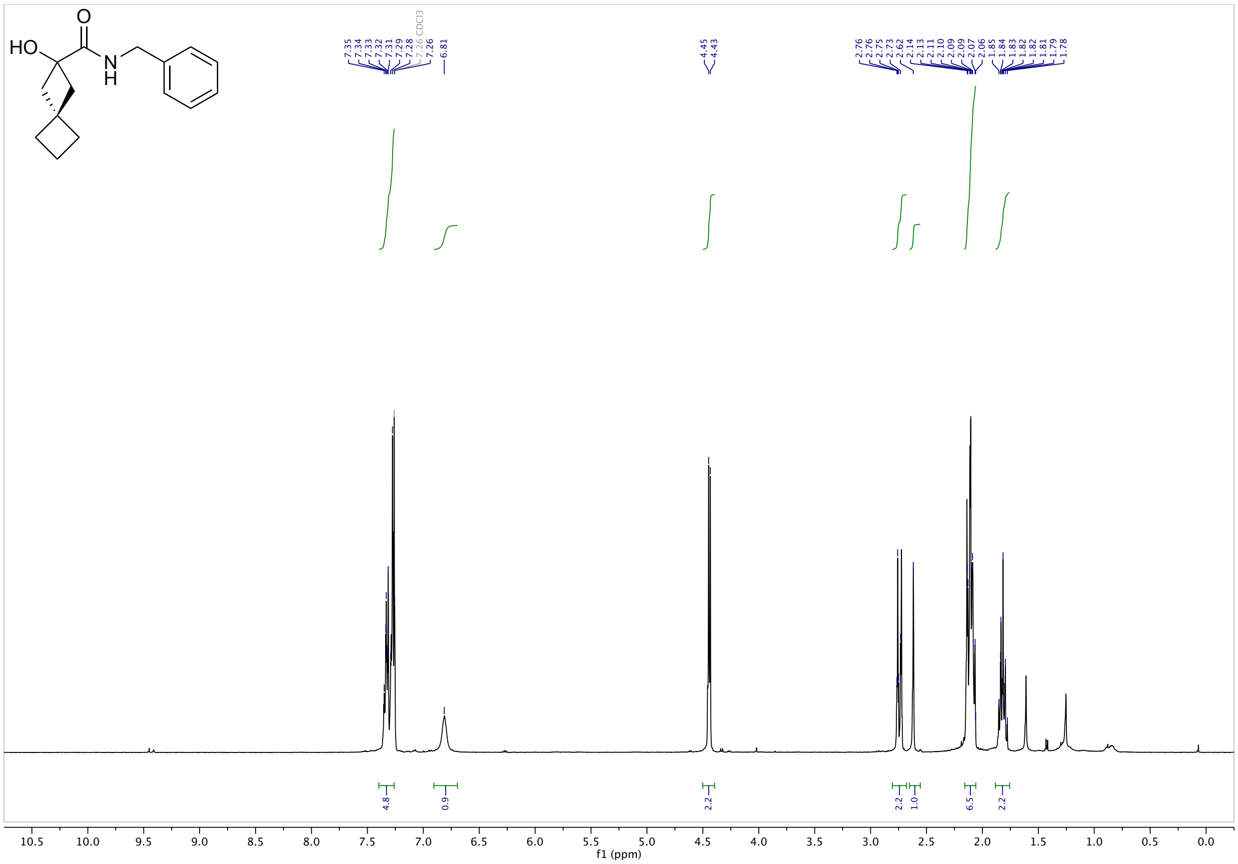

^{13}C NMR (101 MHz, $CDCl_3$) (6)

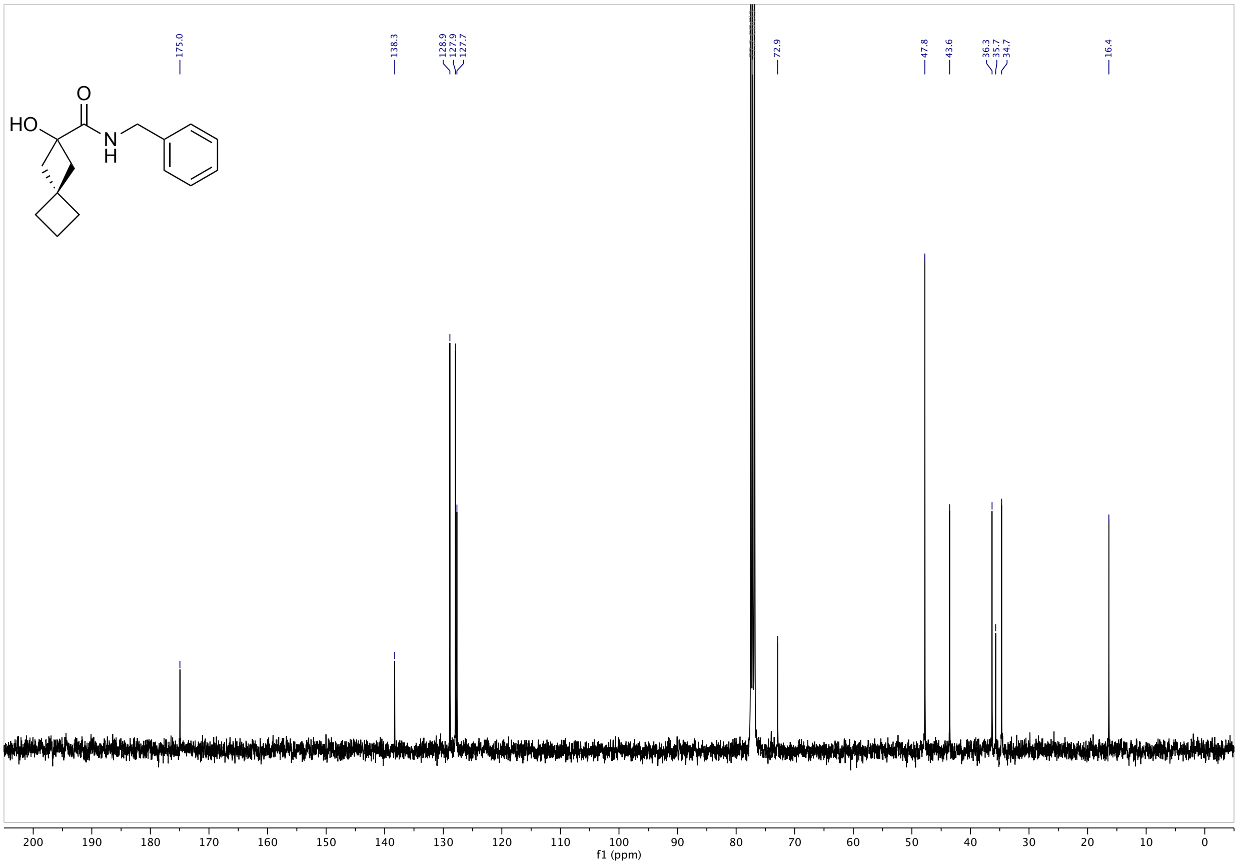

^{1}H NMR (400 MHz, $CDCl_3$) (7)

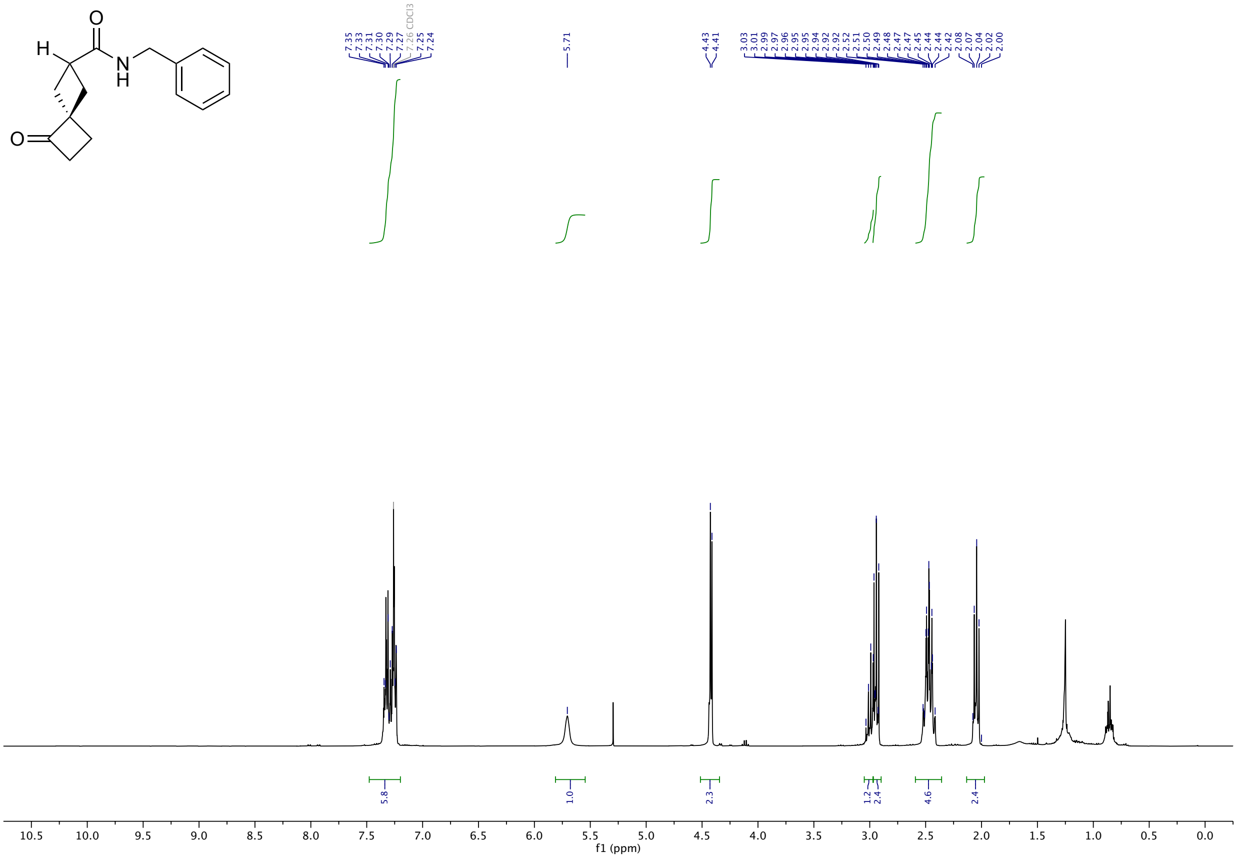

^{13}C NMR (101 MHz, $CDCl_3$) (7)

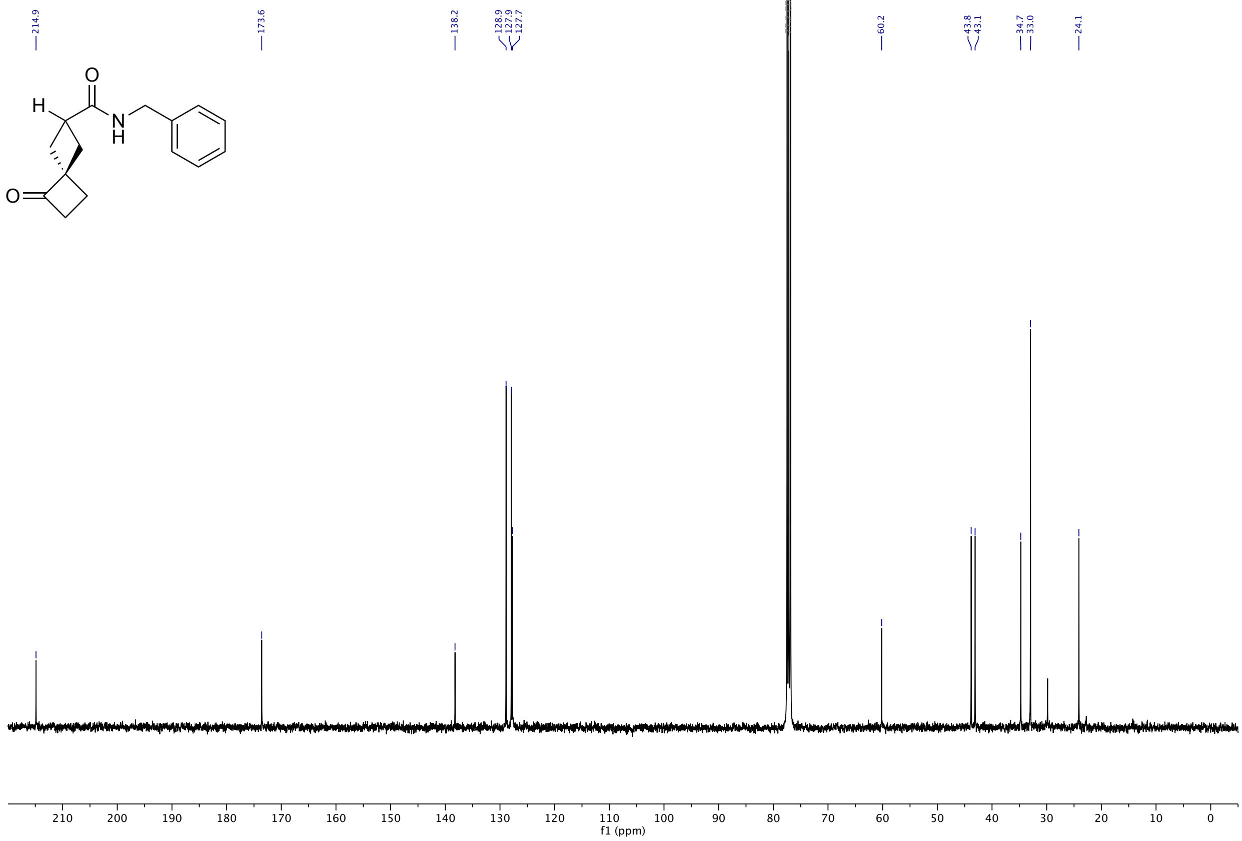

^{1}H NMR (400 MHz, $CDCl_3$) (8)

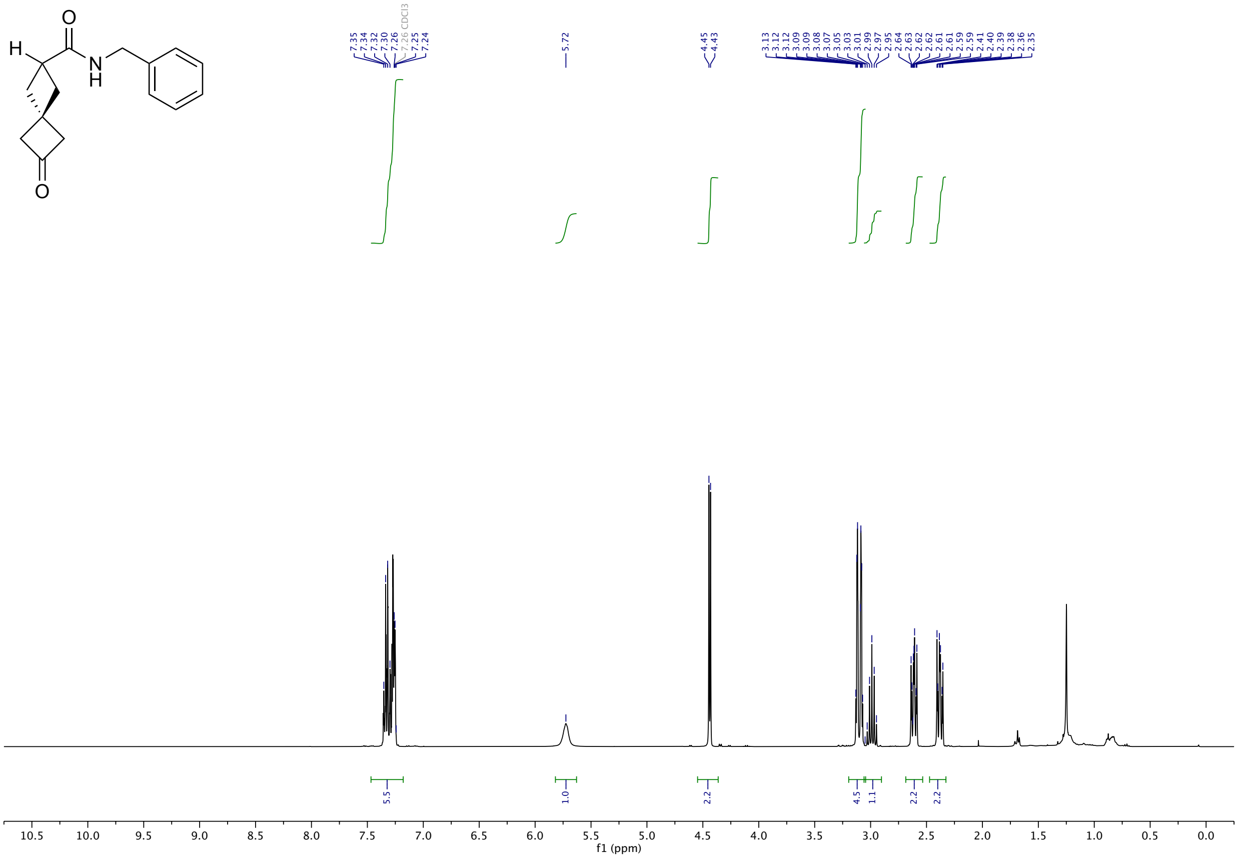

^{13}C NMR (101 MHz, $CDCl_3$) (8)

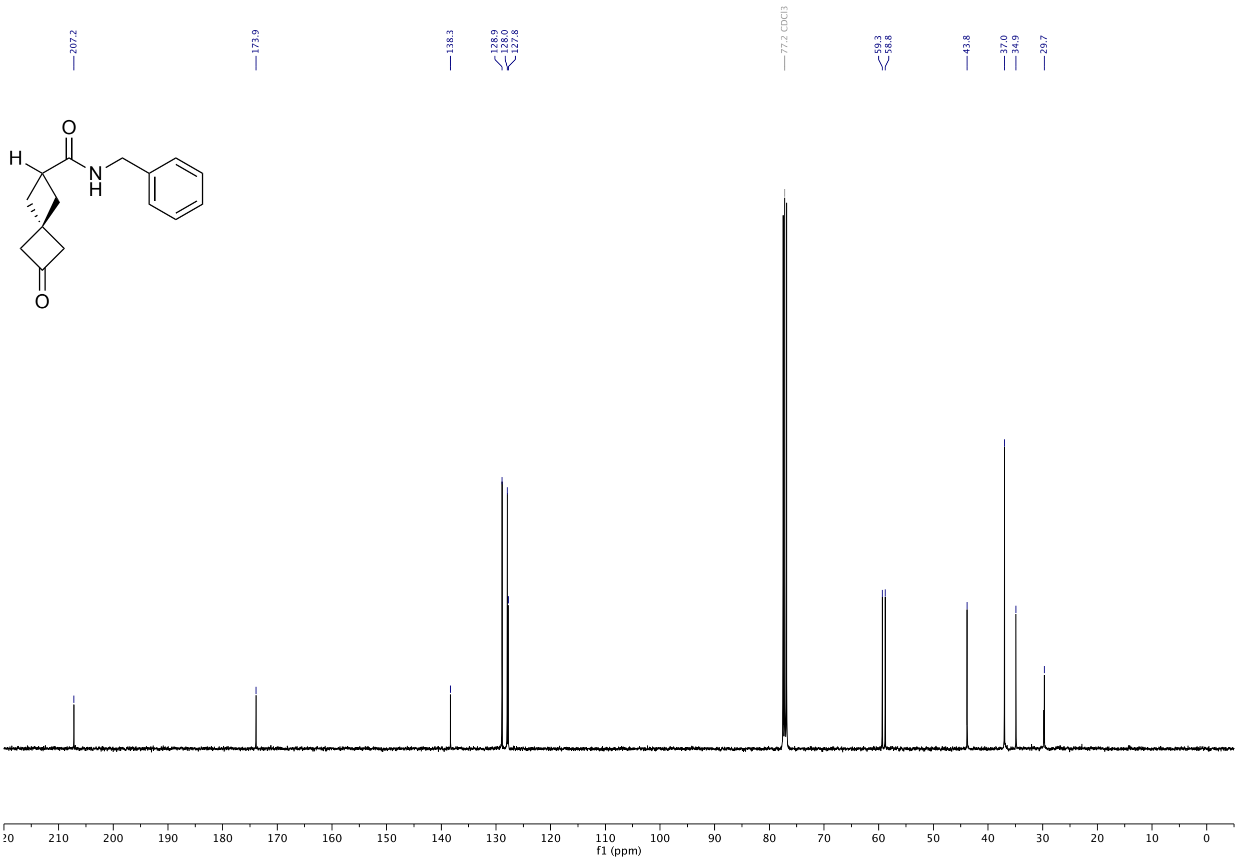

^{1}H NMR (400 MHz, $CDCl_3$) (9)

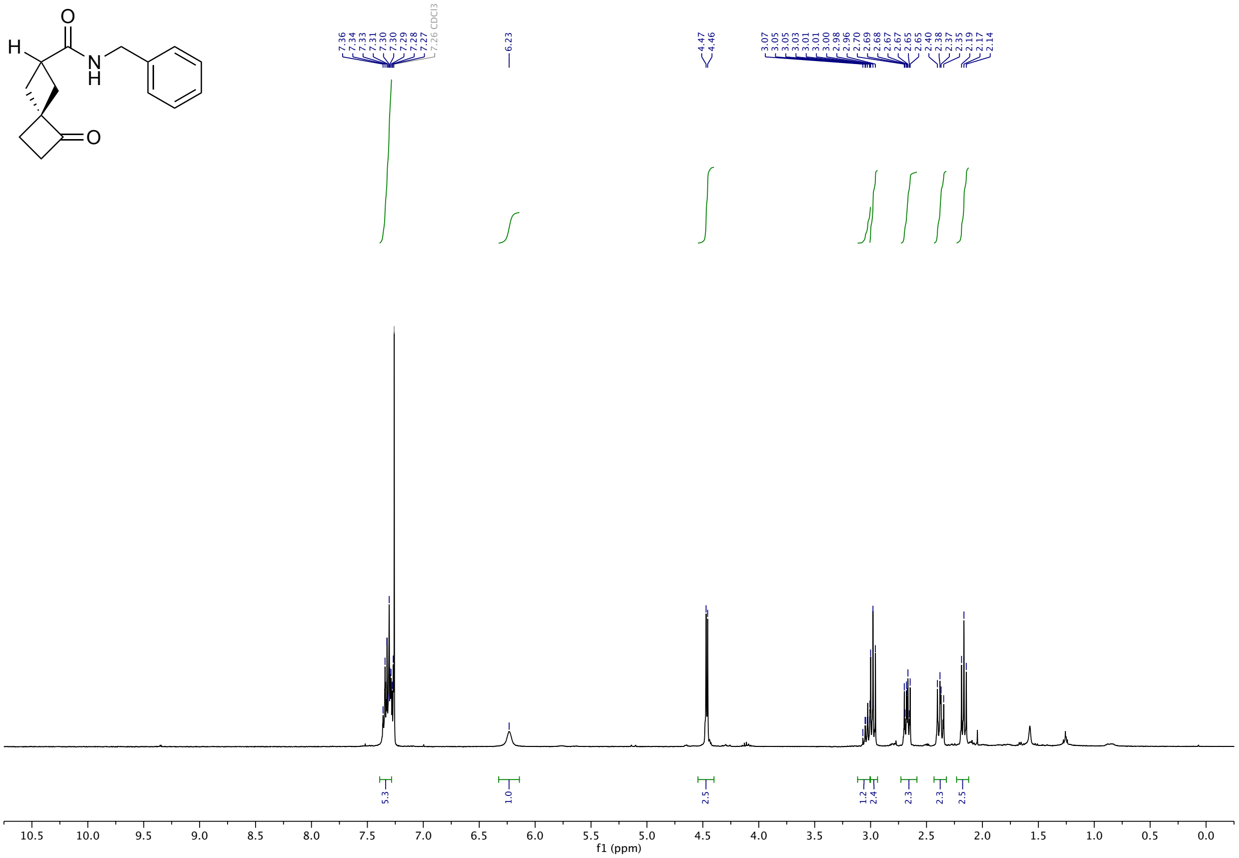

^{13}C NMR (101 MHz, $CDCl_3$) (9)

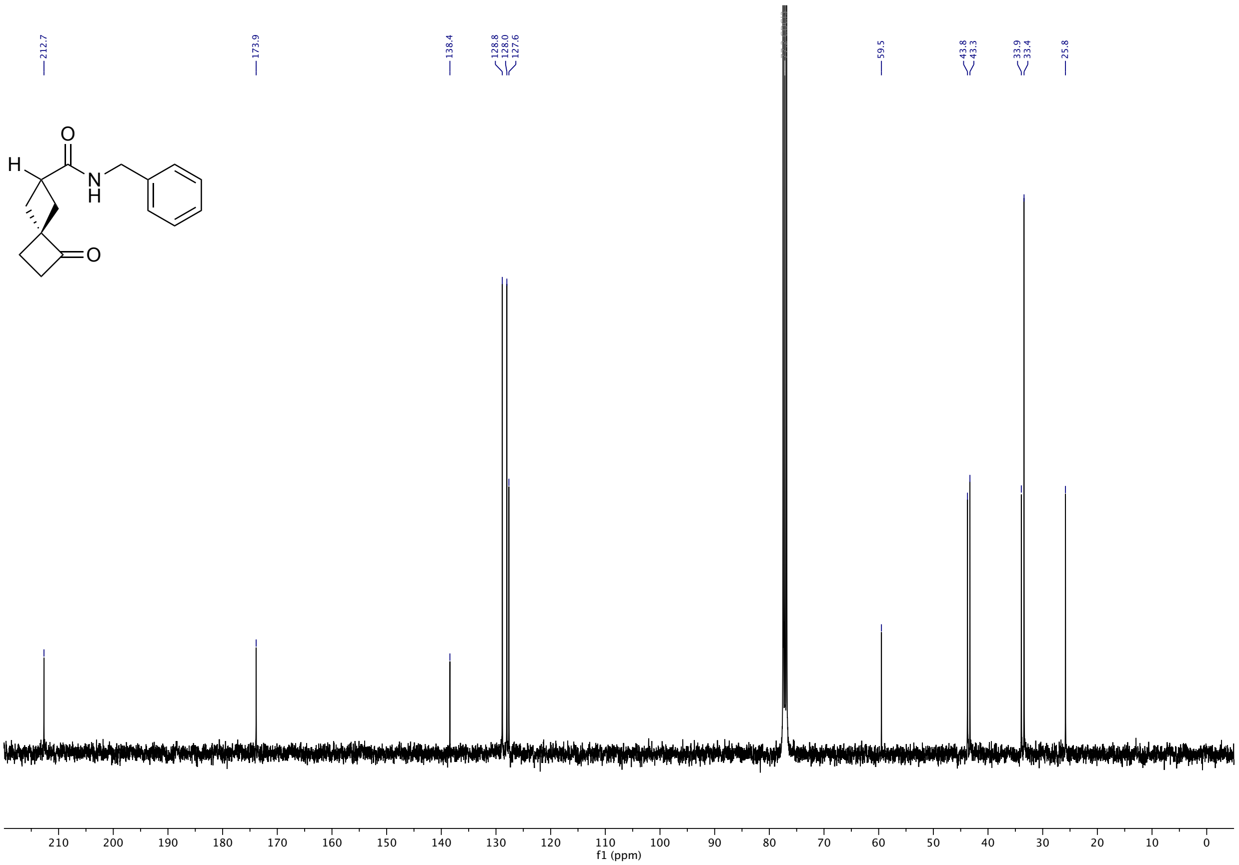

^{1}H NMR (400 MHz, CDCl$_3$) (10)

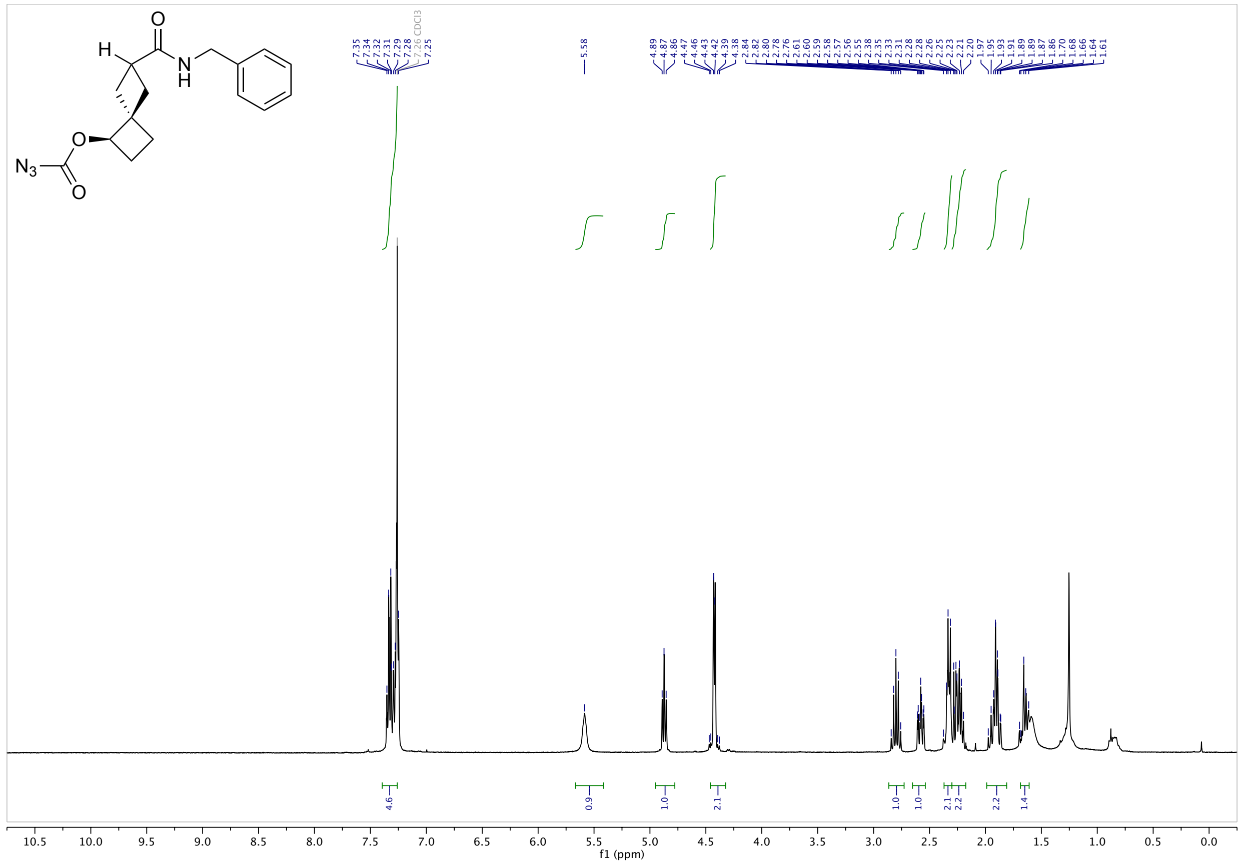

^{13}C NMR (101 MHz, CDCl$_3$) (10)

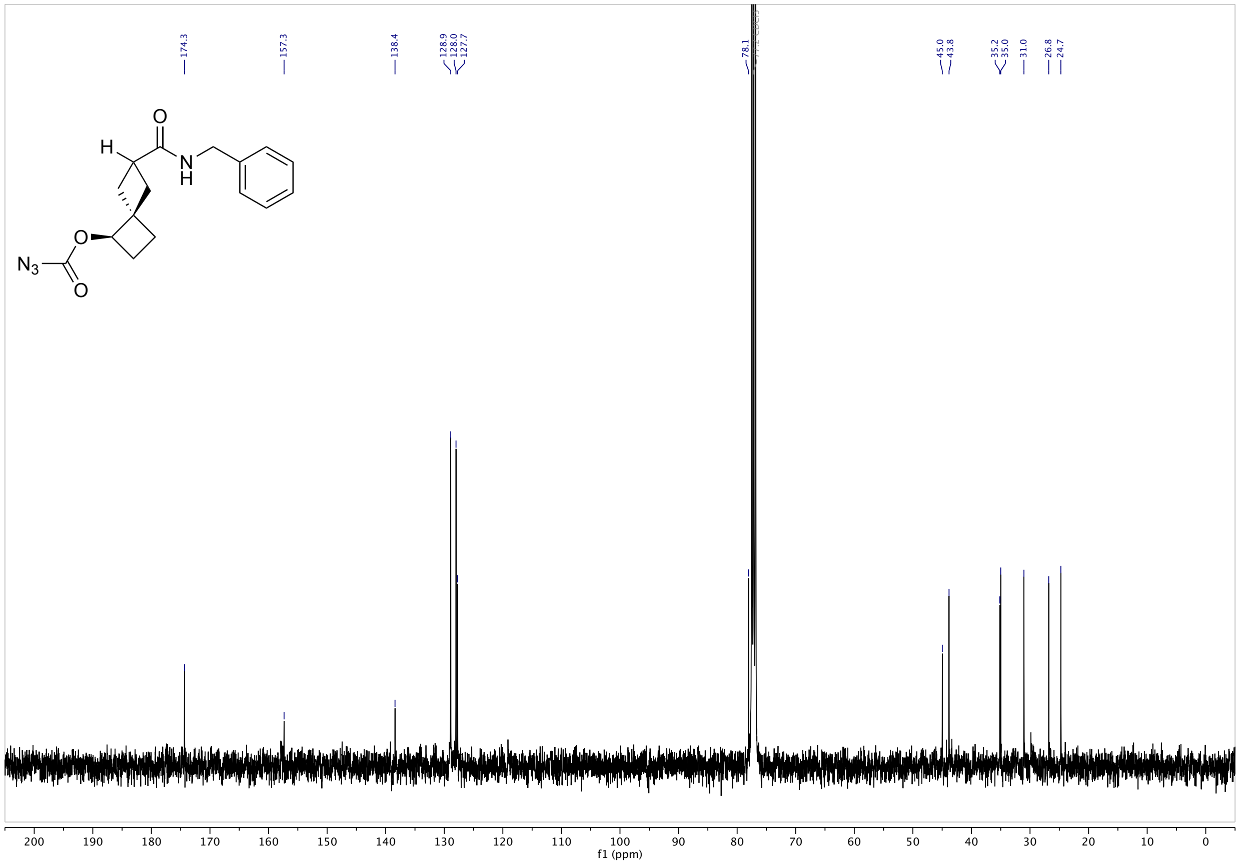

^{1}H NMR (400 MHz, $CDCl_3$) (11)

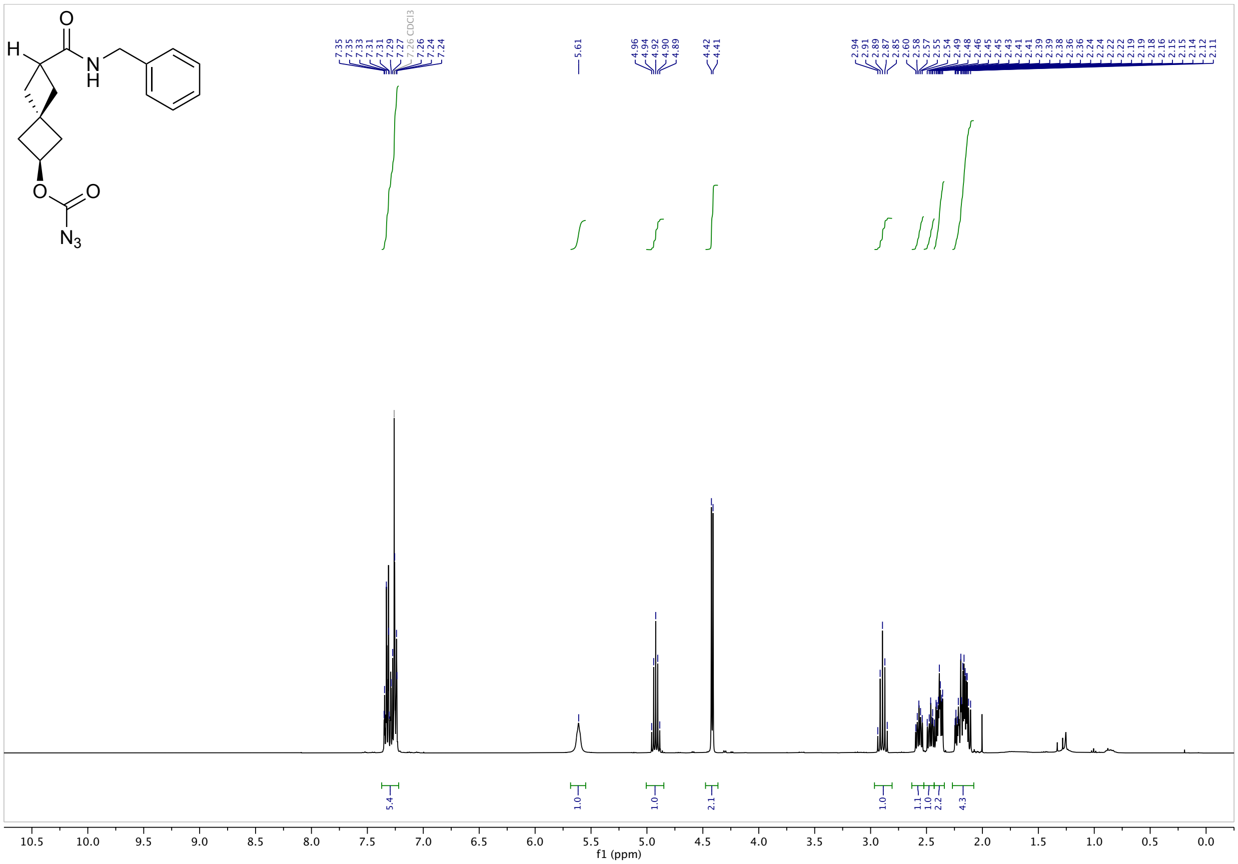

^{13}C NMR (101 MHz, $CDCl_3$) (11)

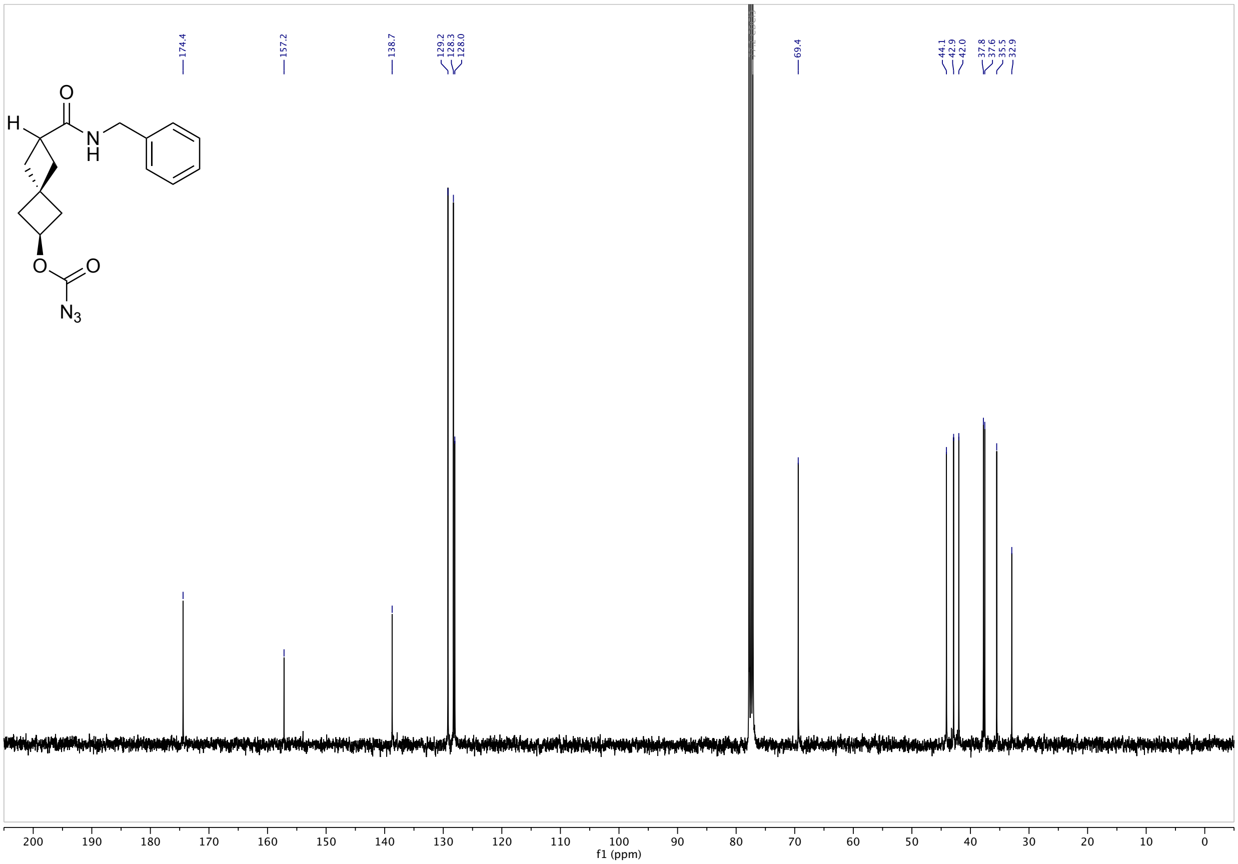

^{1}H NMR (400 MHz, $CDCl_3$) (12)

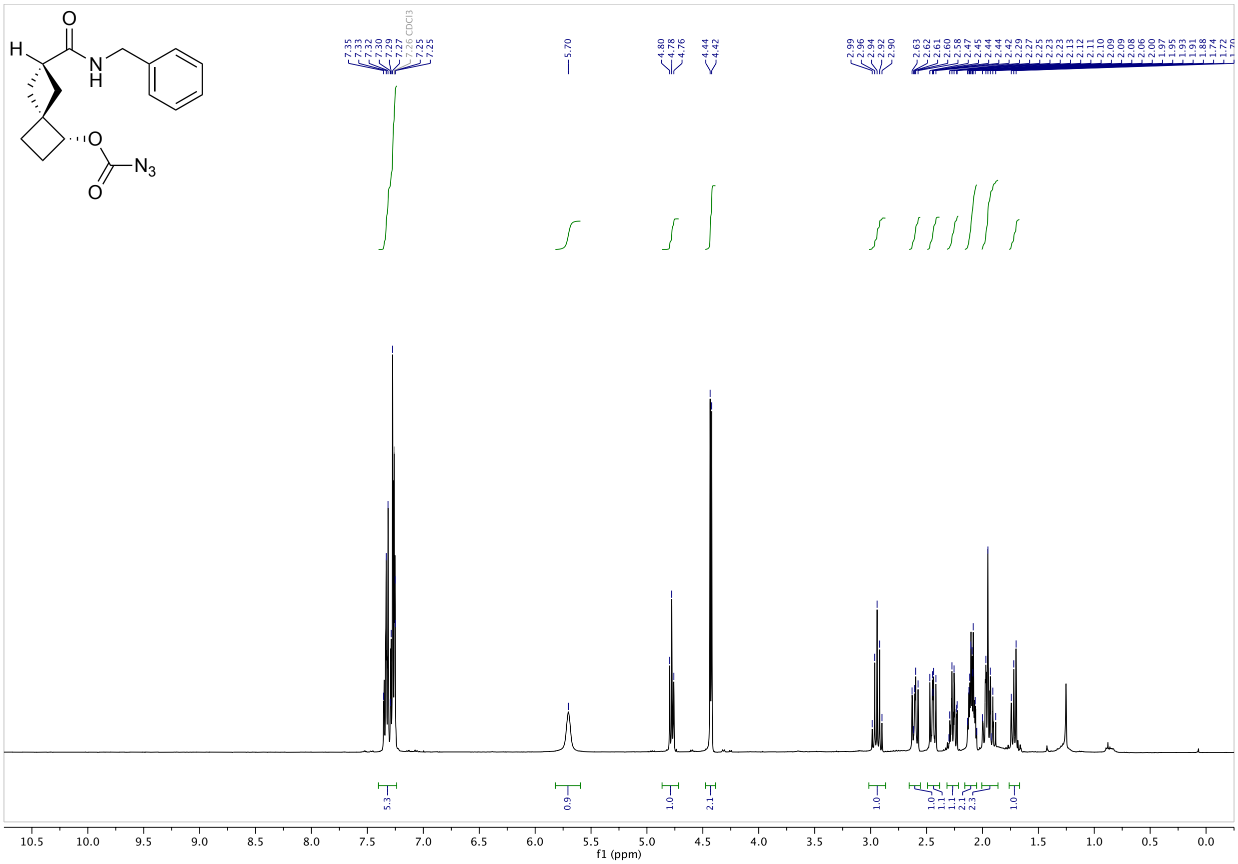

^{13}C NMR (101 MHz, $CDCl_3$) (12)

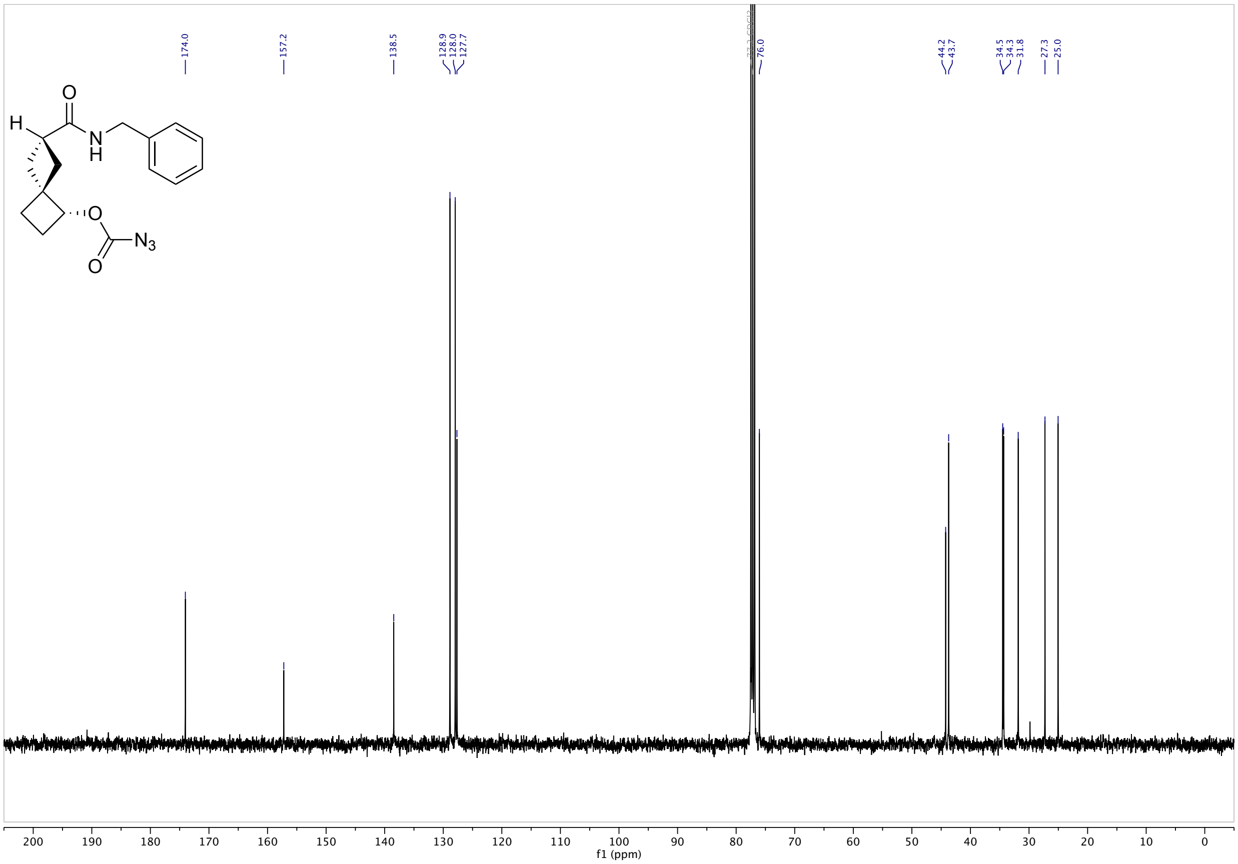

^{1}H NMR (400 MHz, $CDCl_3$) (13)

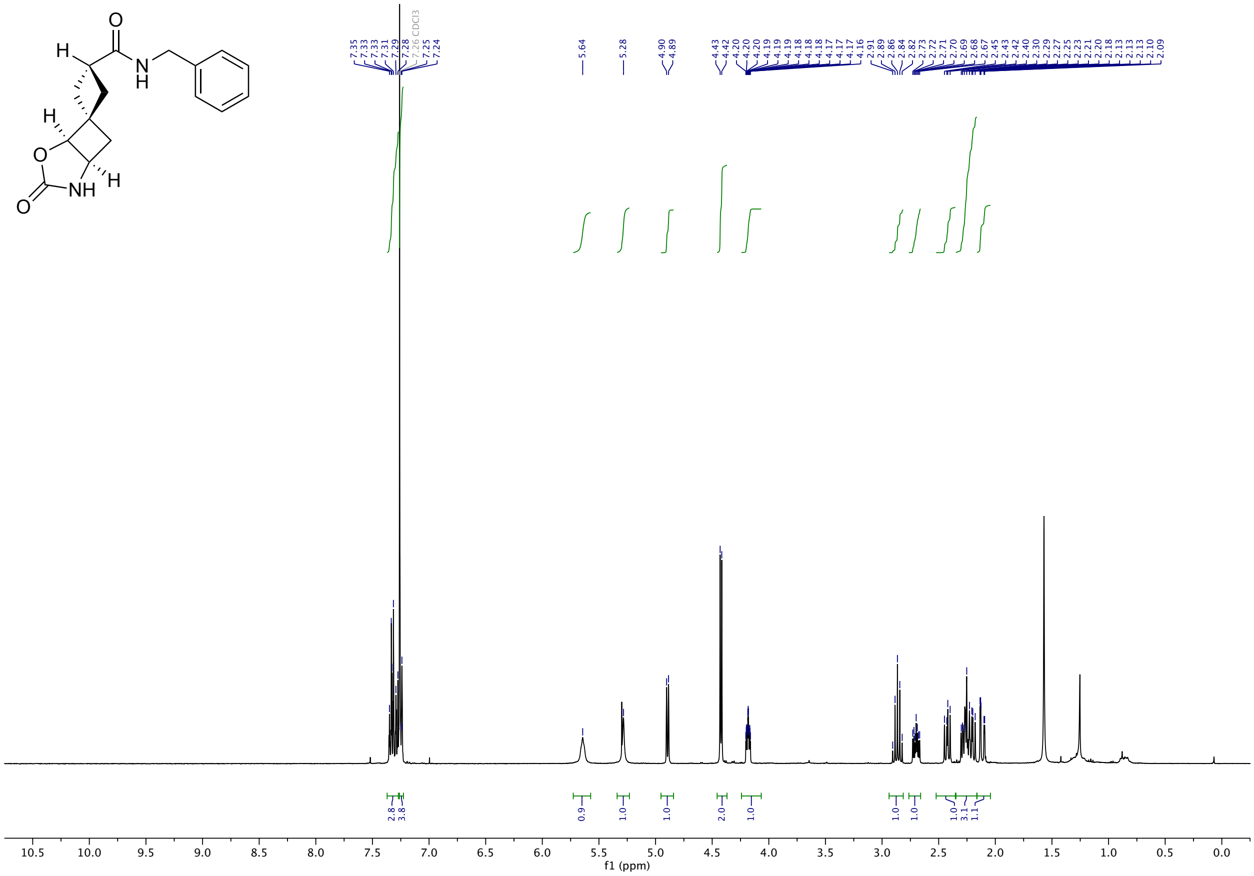

^{13}C NMR (101 MHz, $CDCl_3$) (13)

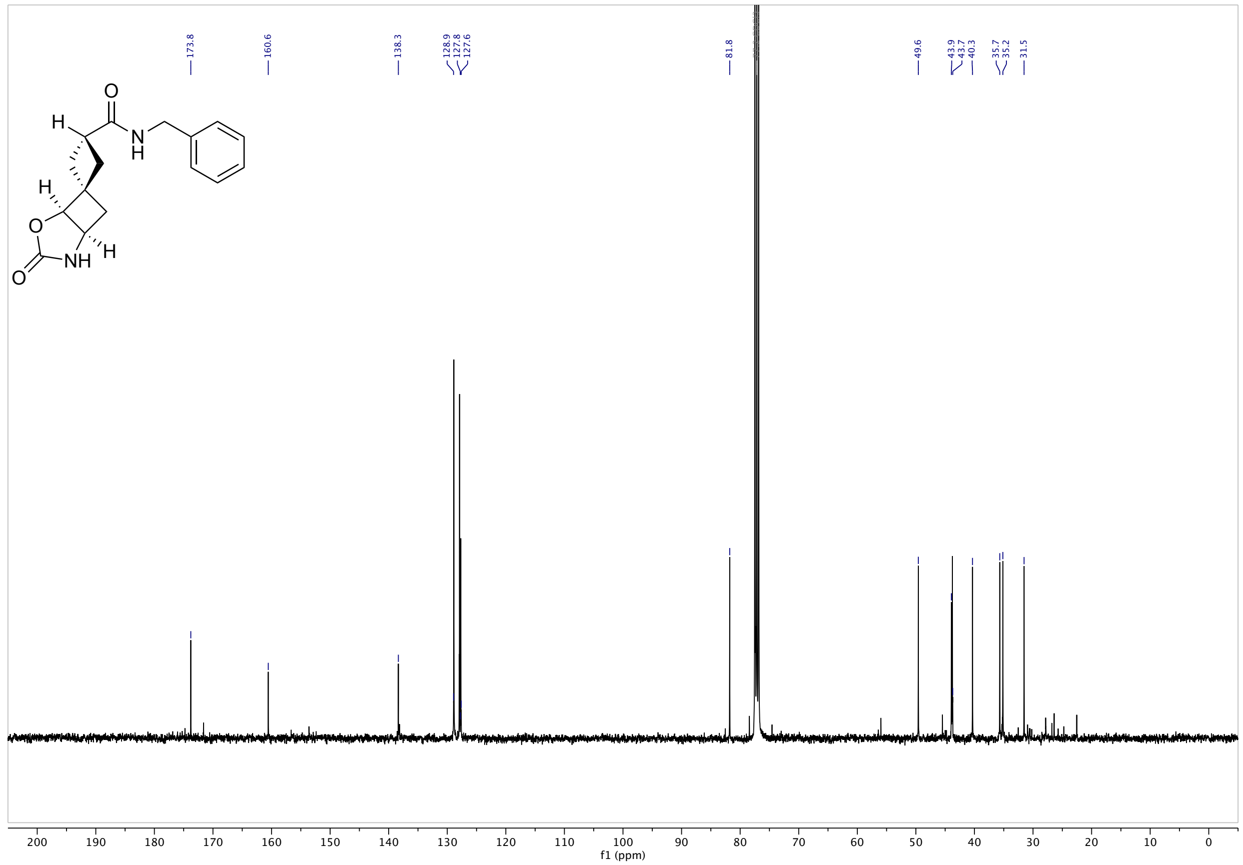

^{1}H NMR (400 MHz, CDCl$_3$) (14)

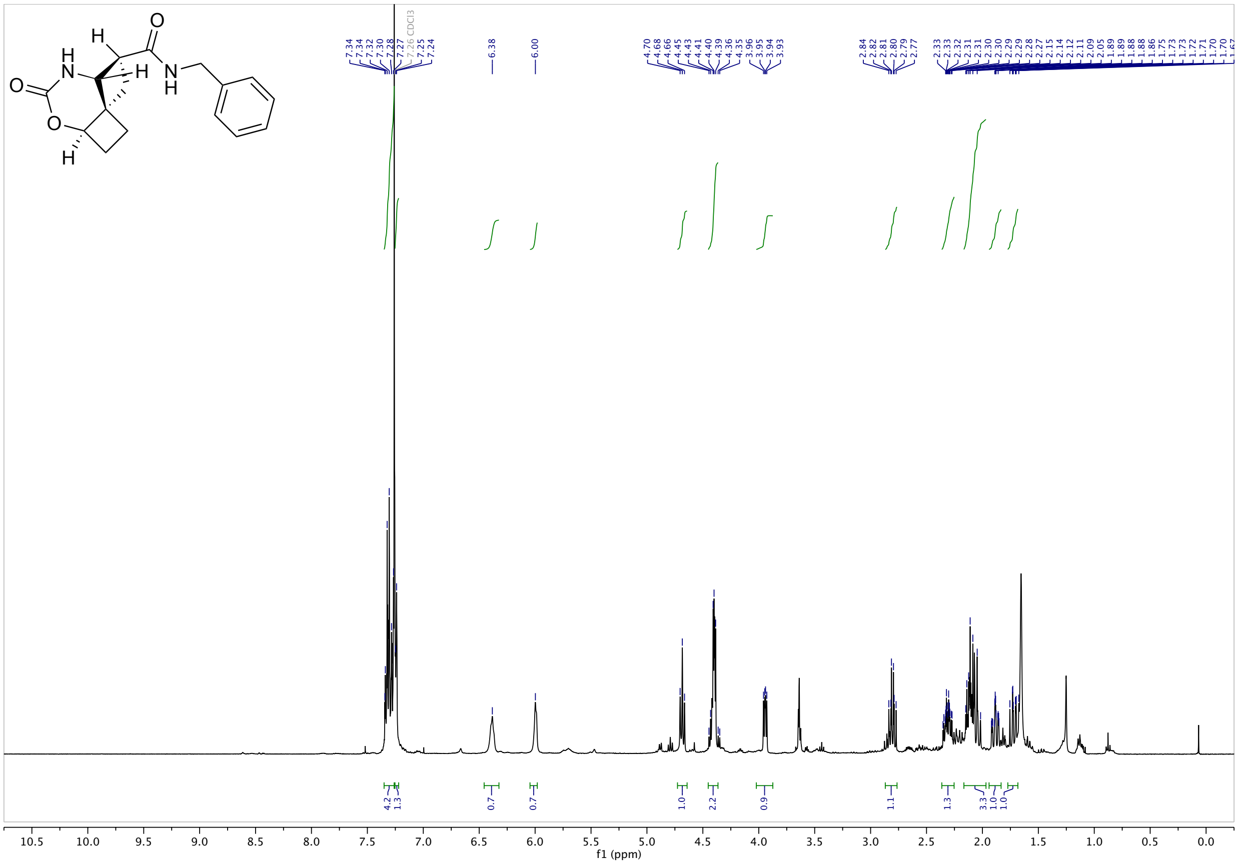

^{13}C NMR (101 MHz, CDCl$_3$) (14)

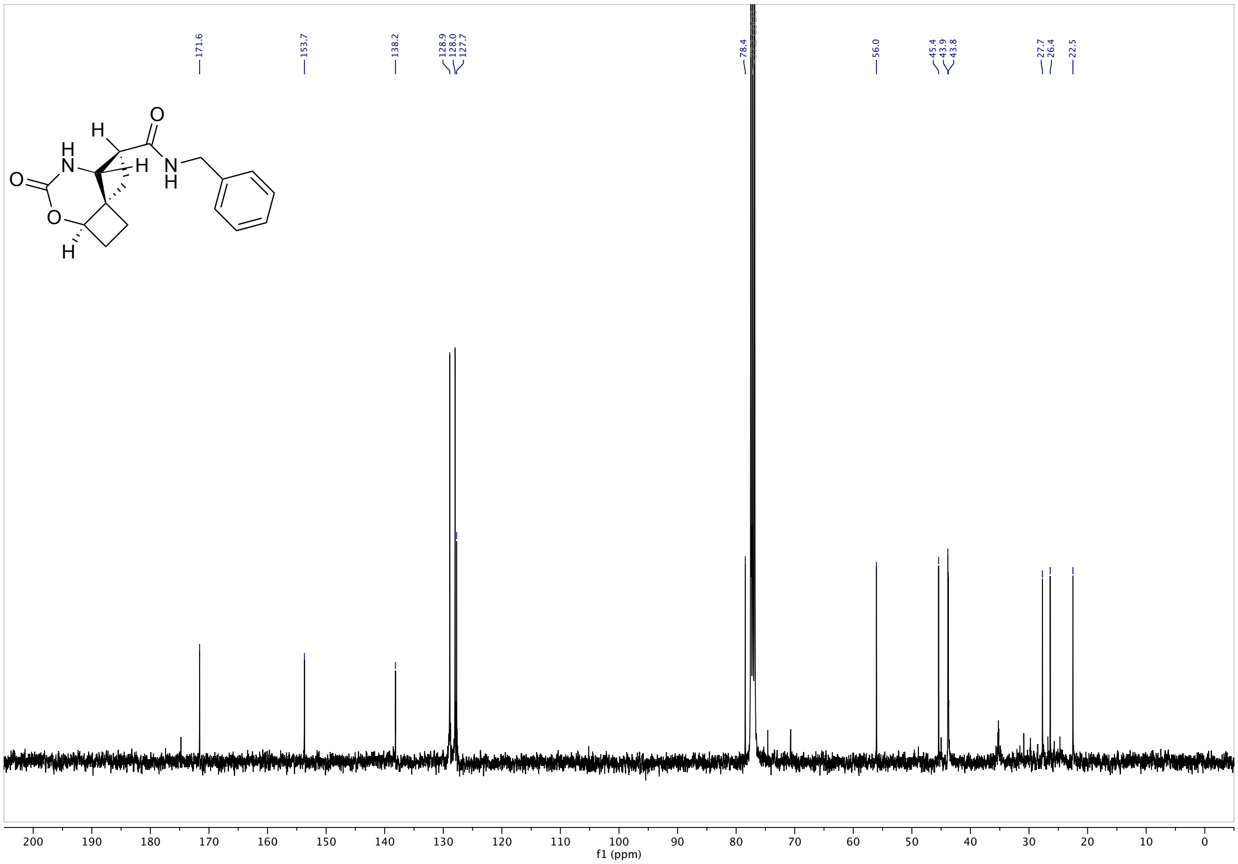

^{1}H NMR (400 MHz, $CDCl_3$) (15)

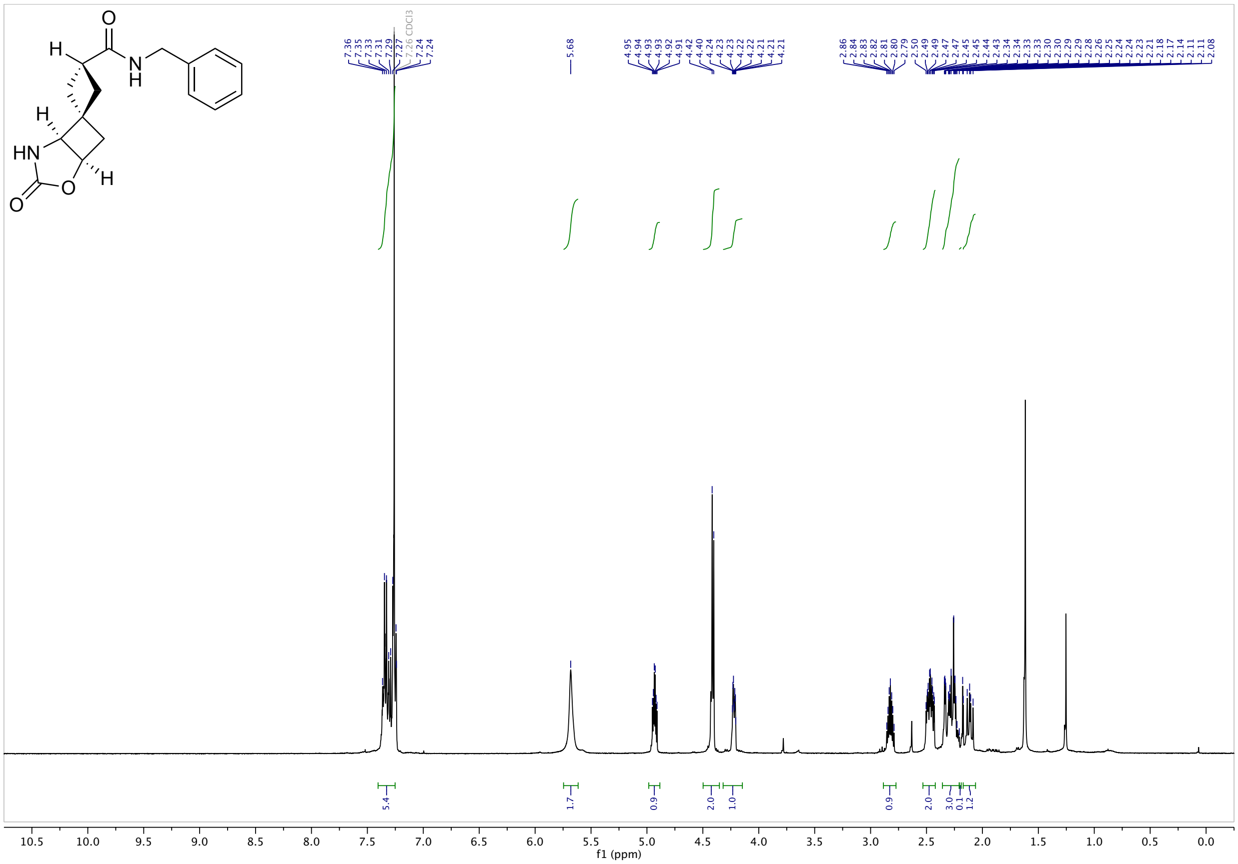

^{13}C NMR (101 MHz, $CDCl_3$) (15)

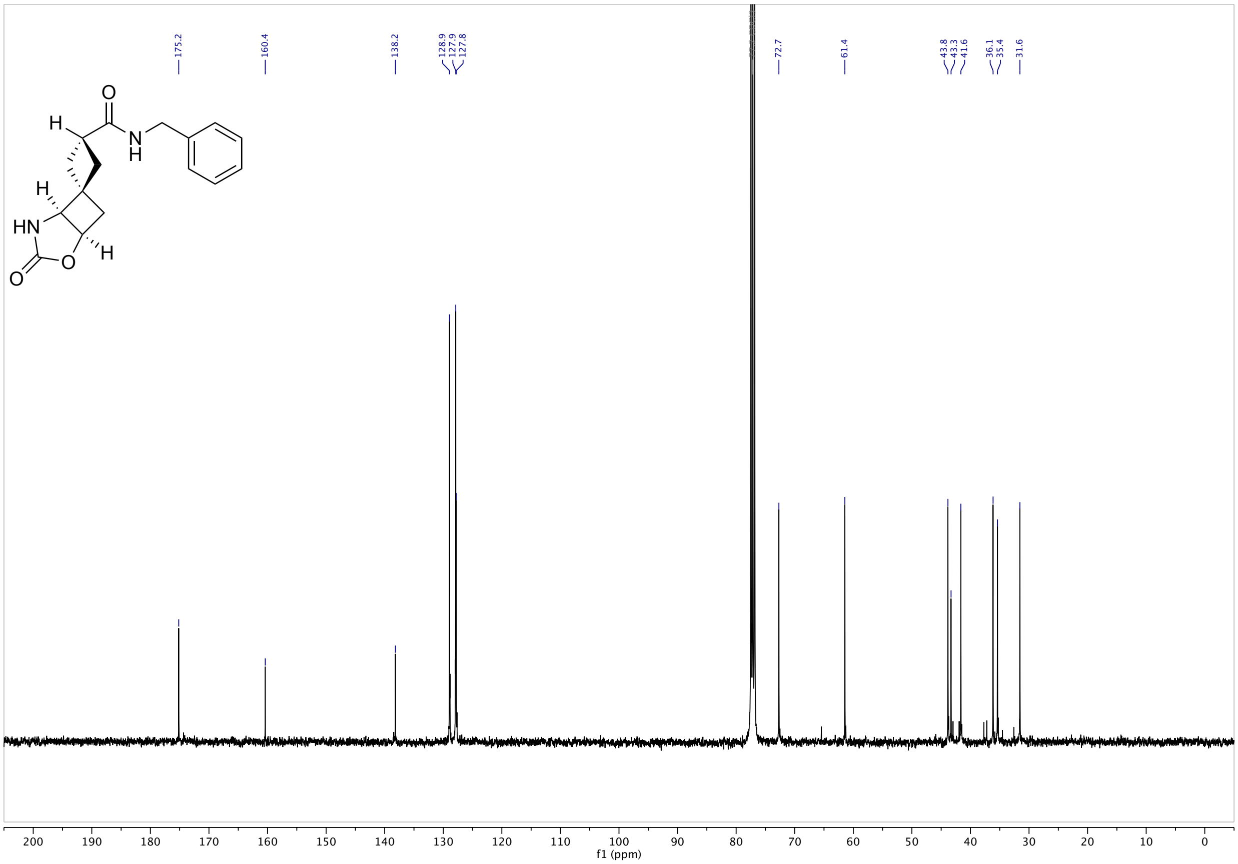

^{1}H NMR (400 MHz, $CDCl_3$) (16)

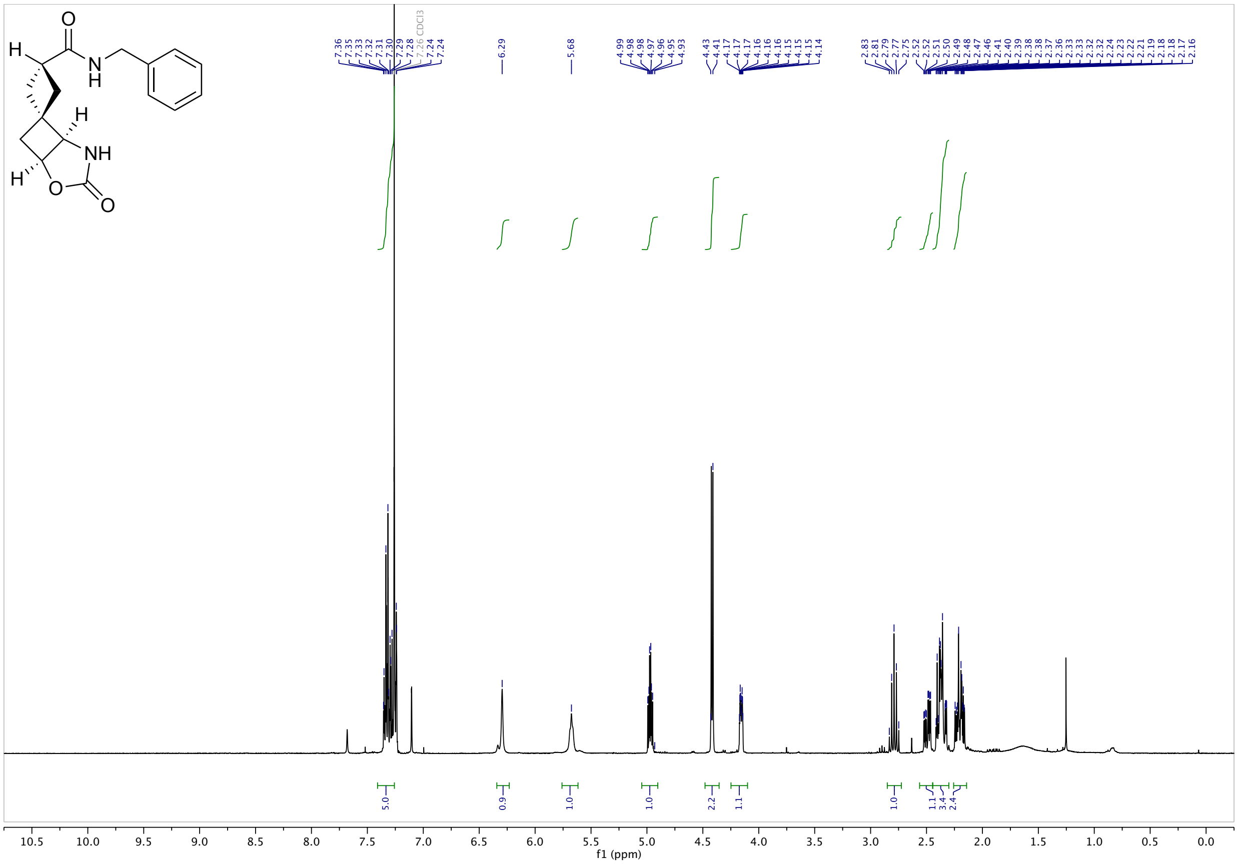

^{13}C NMR (101 MHz, $CDCl_3$) (16)

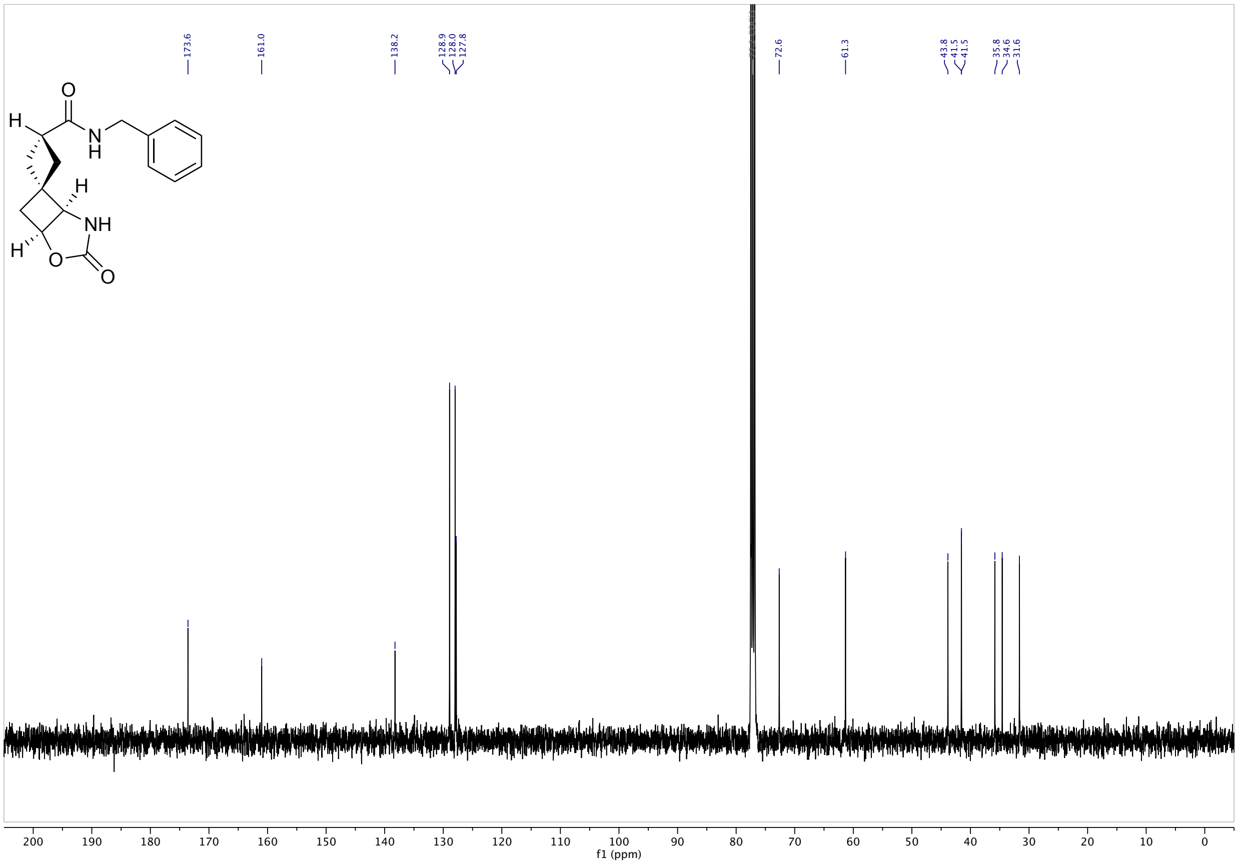

^{1}H NMR (400 MHz, CDCl$_3$) (17)

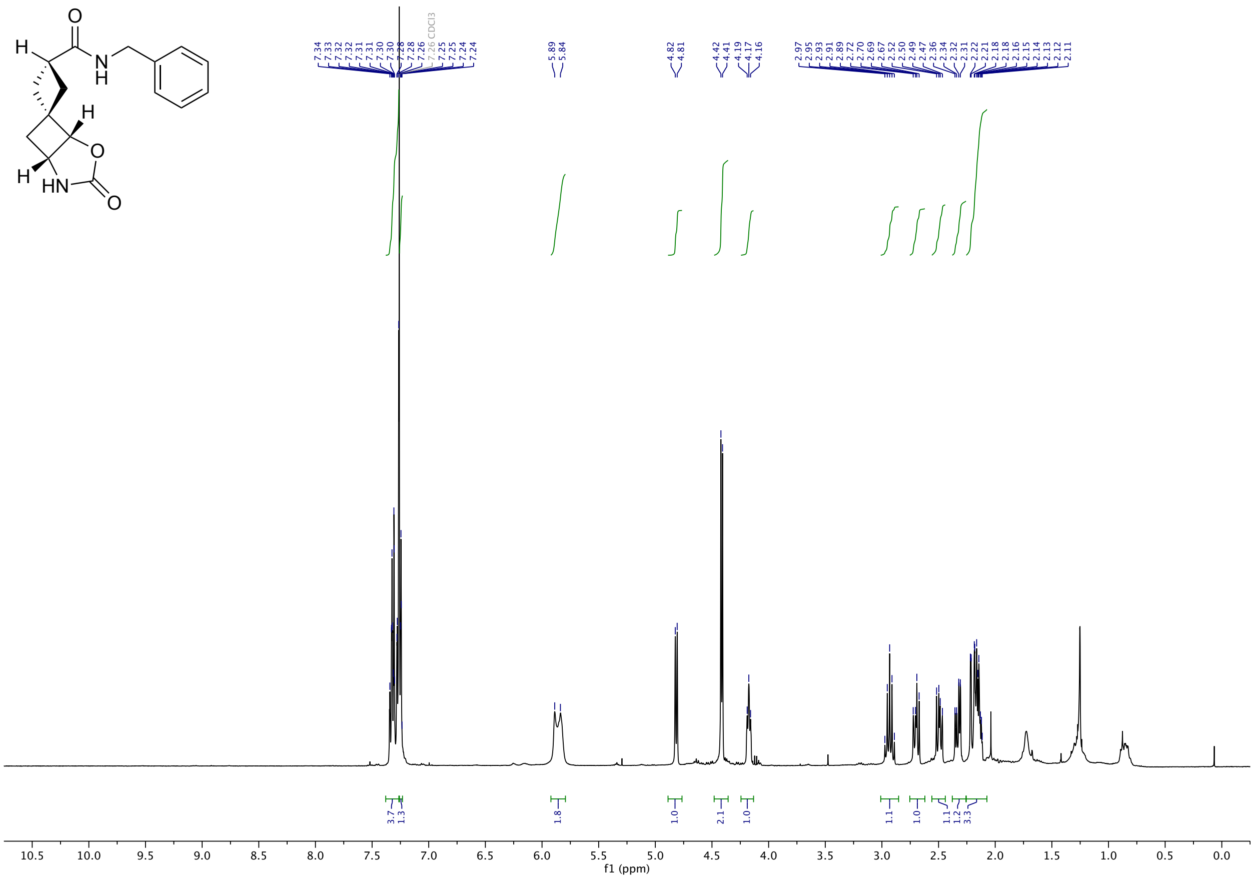

^{13}C NMR (101 MHz, CDCl$_3$) (17)

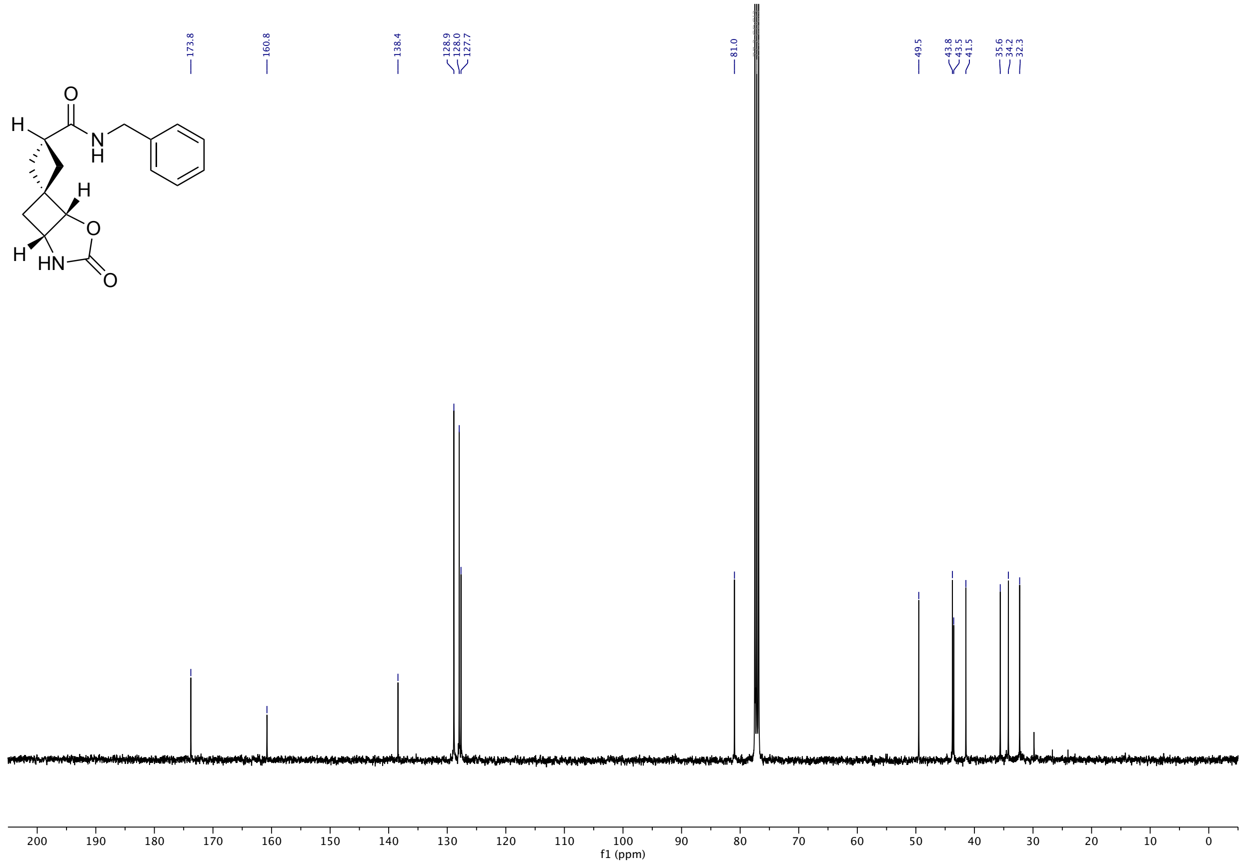

^{1}H NMR (400 MHz, $CDCl_3$) (S1)

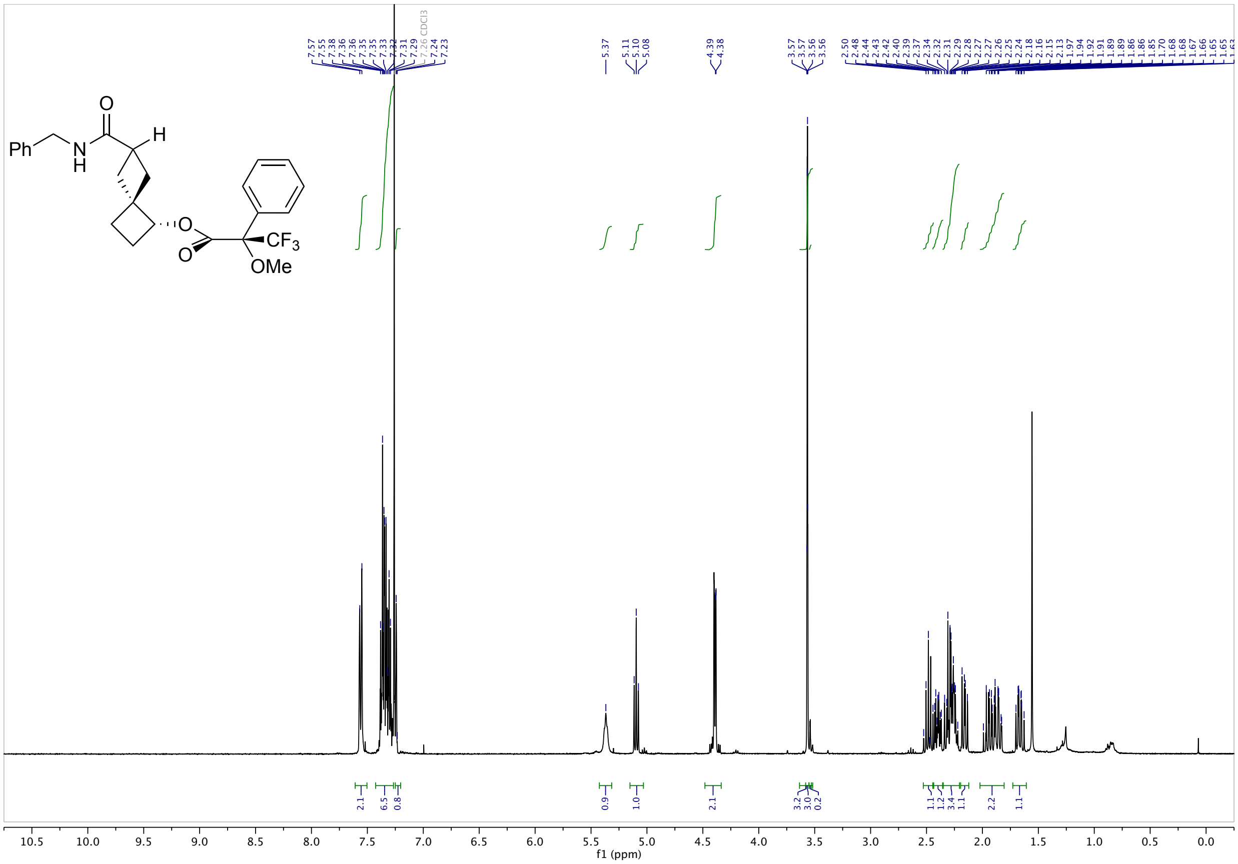

^{13}C NMR (101 MHz, $CDCl_3$) (S1)

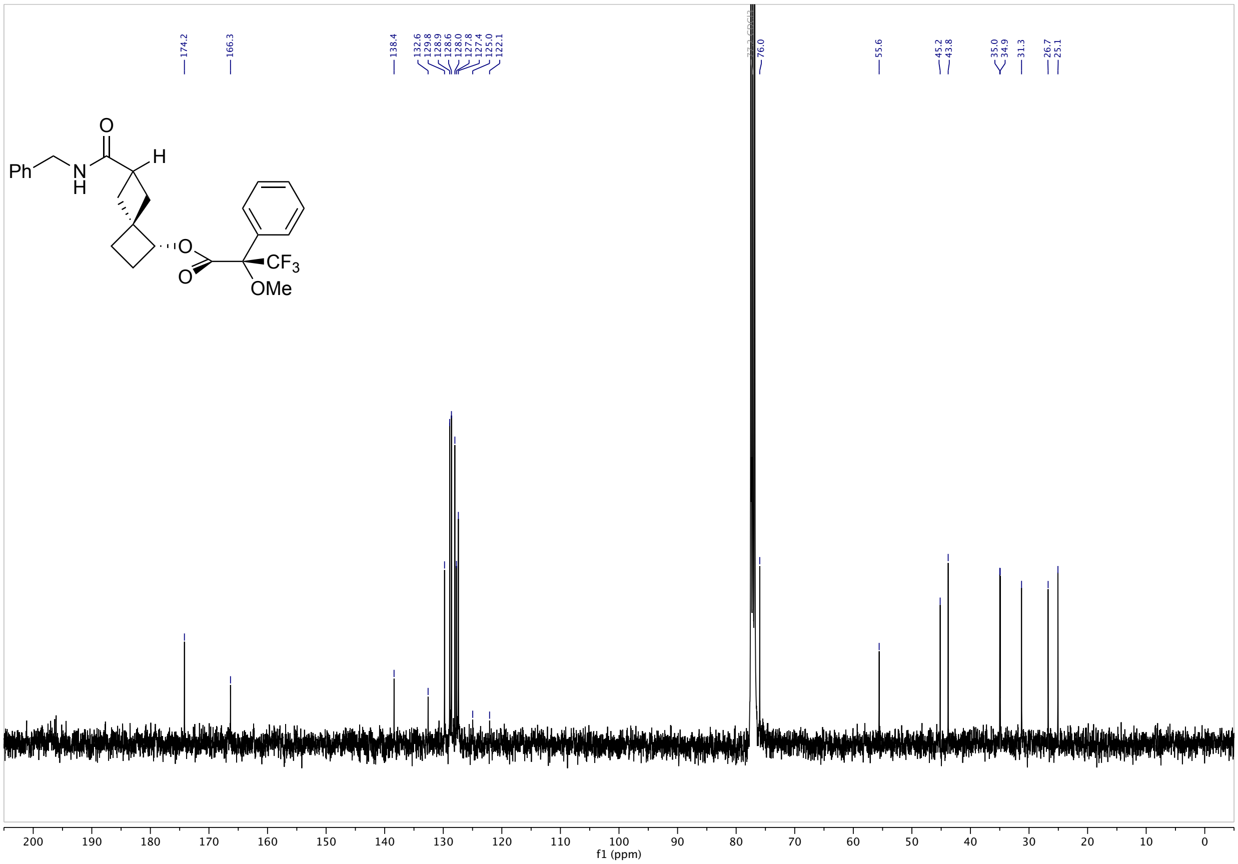

^{1}H NMR (400 MHz, CDCl$_3$) (S2)

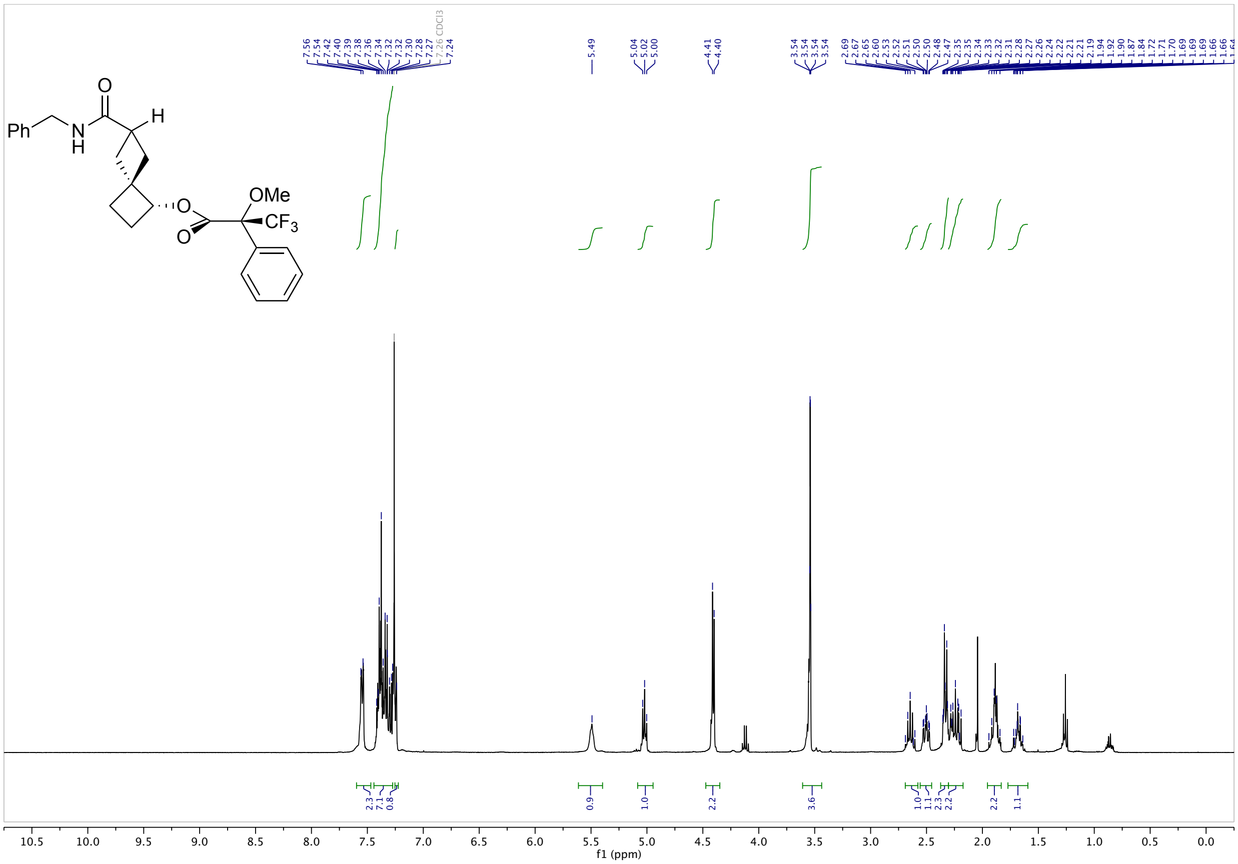

^{13}C NMR (101 MHz, CDCl$_3$) (S2)

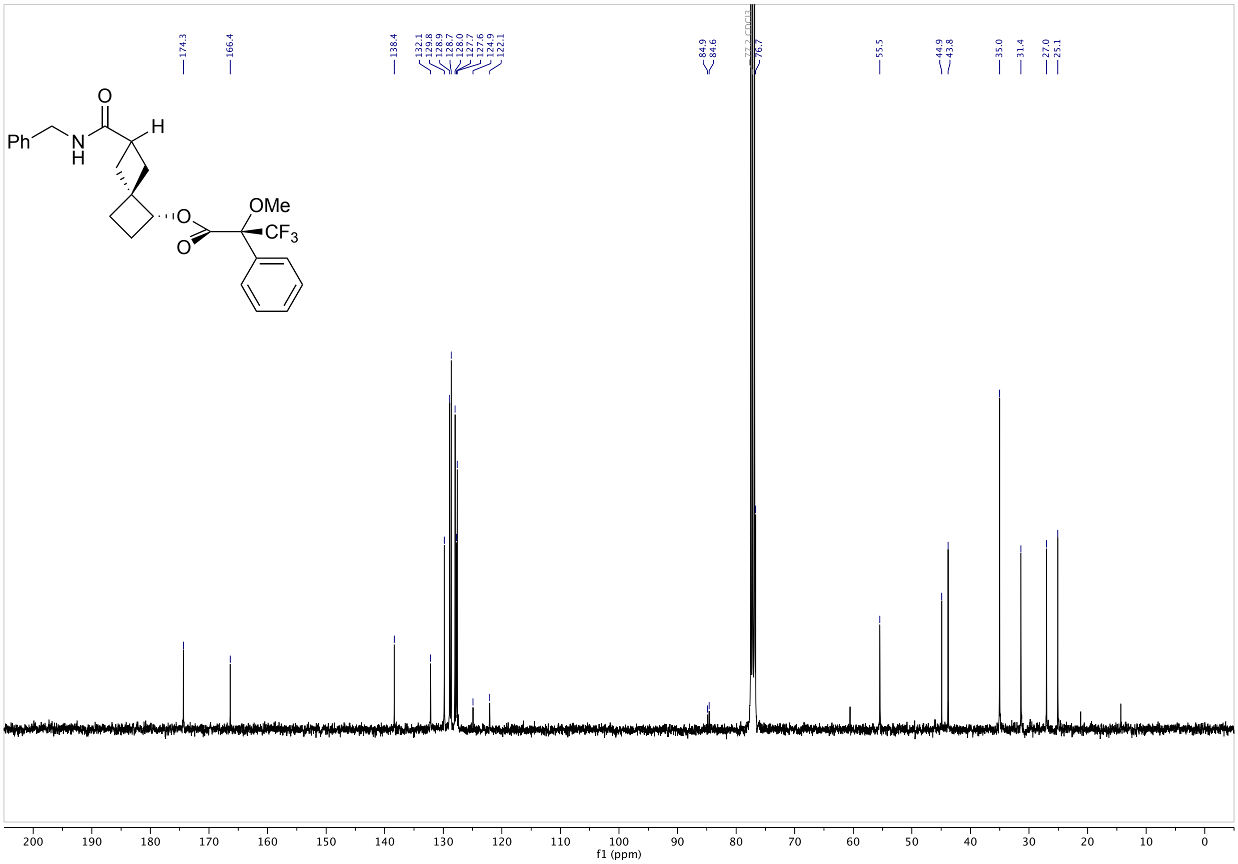

^{1}H NMR (400 MHz, $CDCl_3$) (S3)

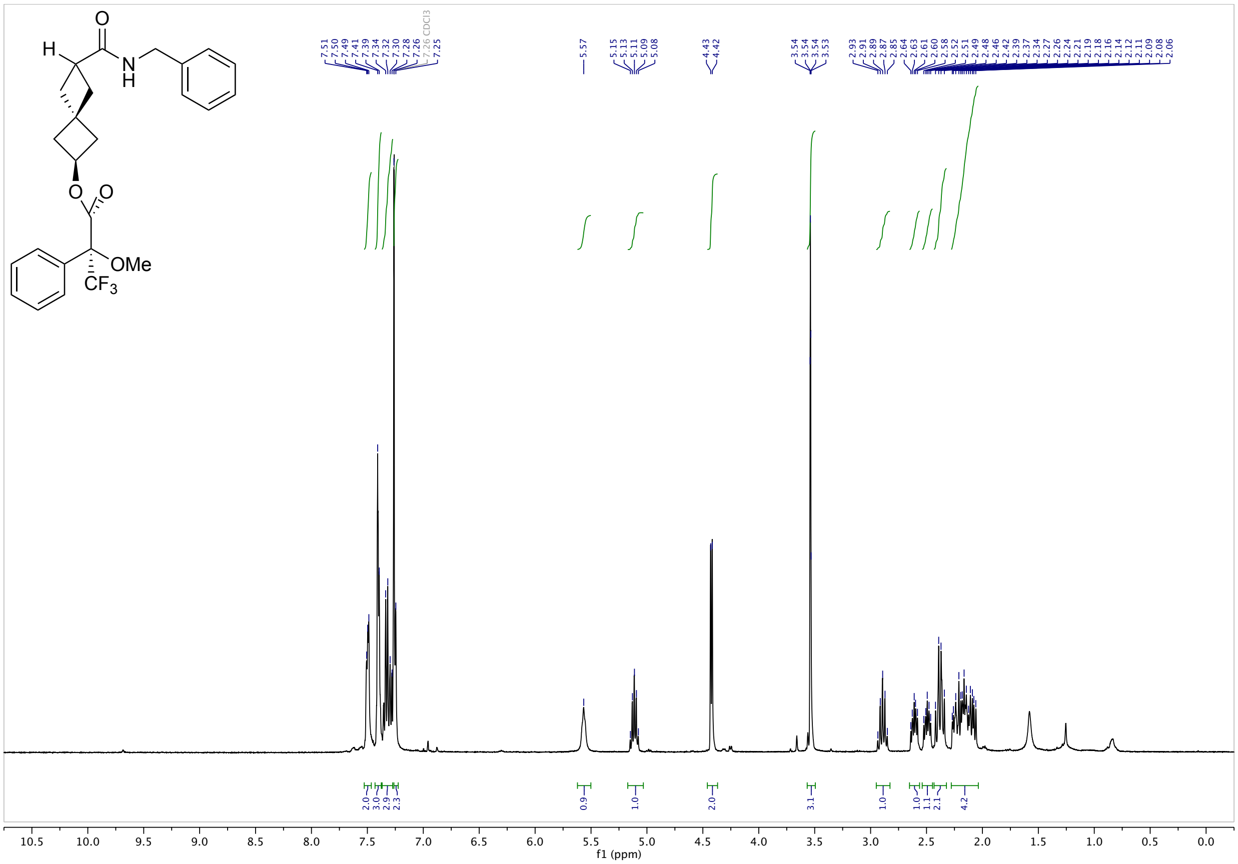

^{13}C NMR (101 MHz, $CDCl_3$) (S3)

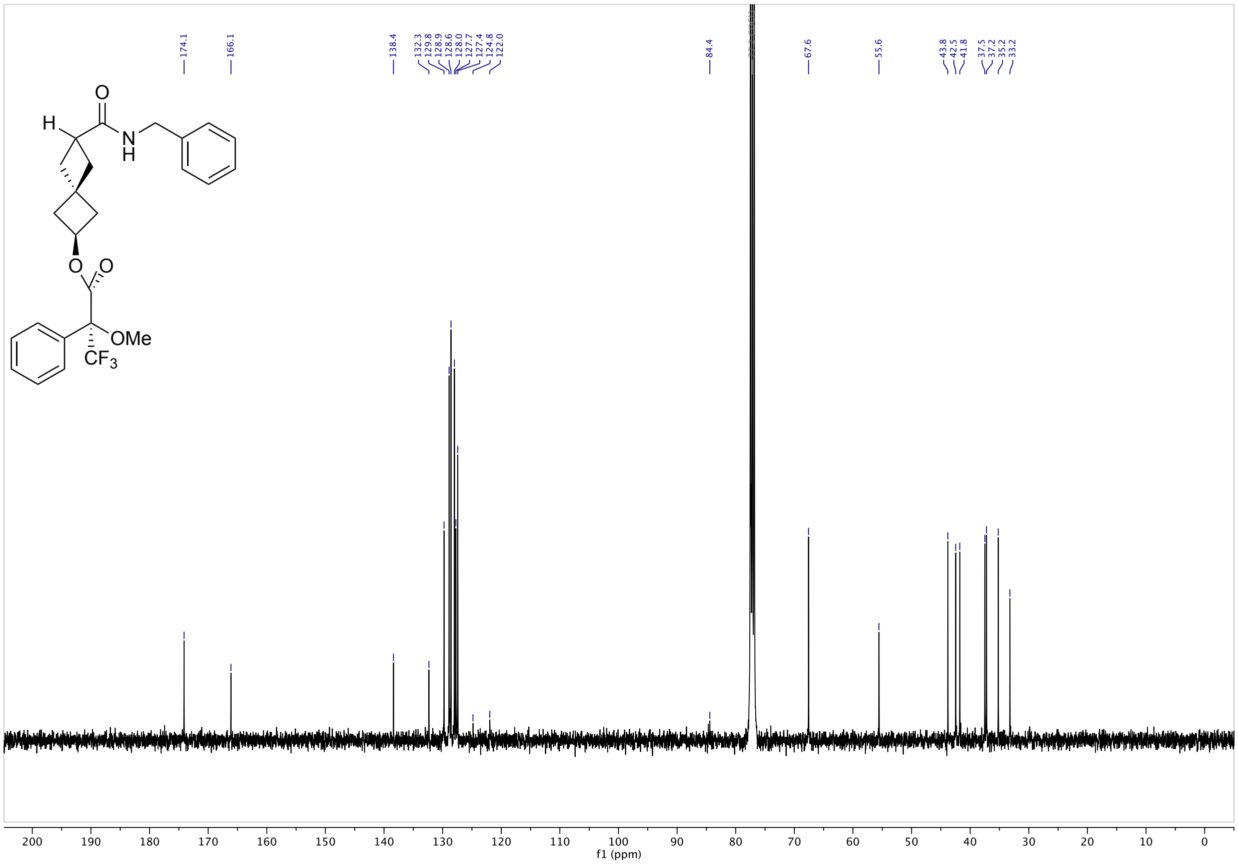

^{1}H NMR (400 MHz, $CDCl_3$) (S4)

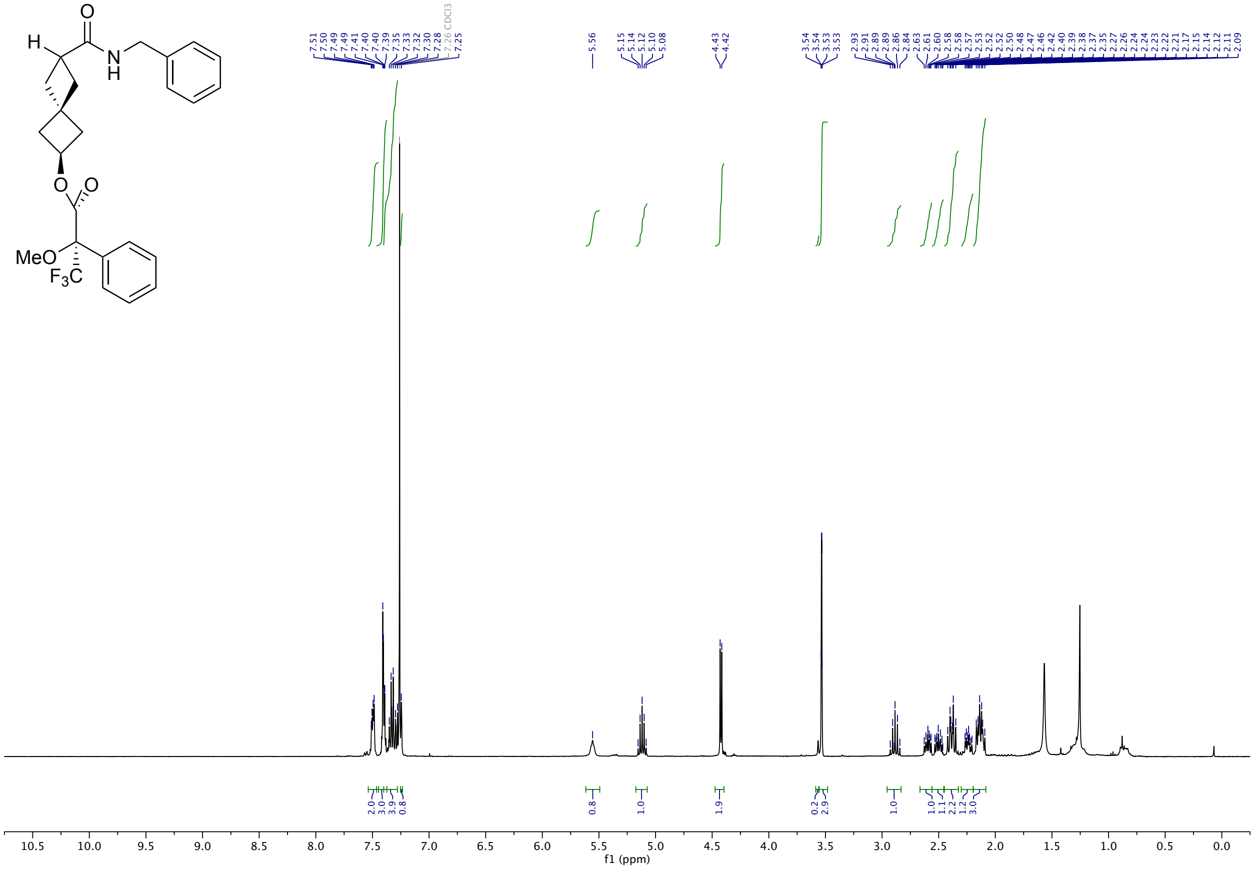

^{13}C NMR (101 MHz, $CDCl_3$) (S4)

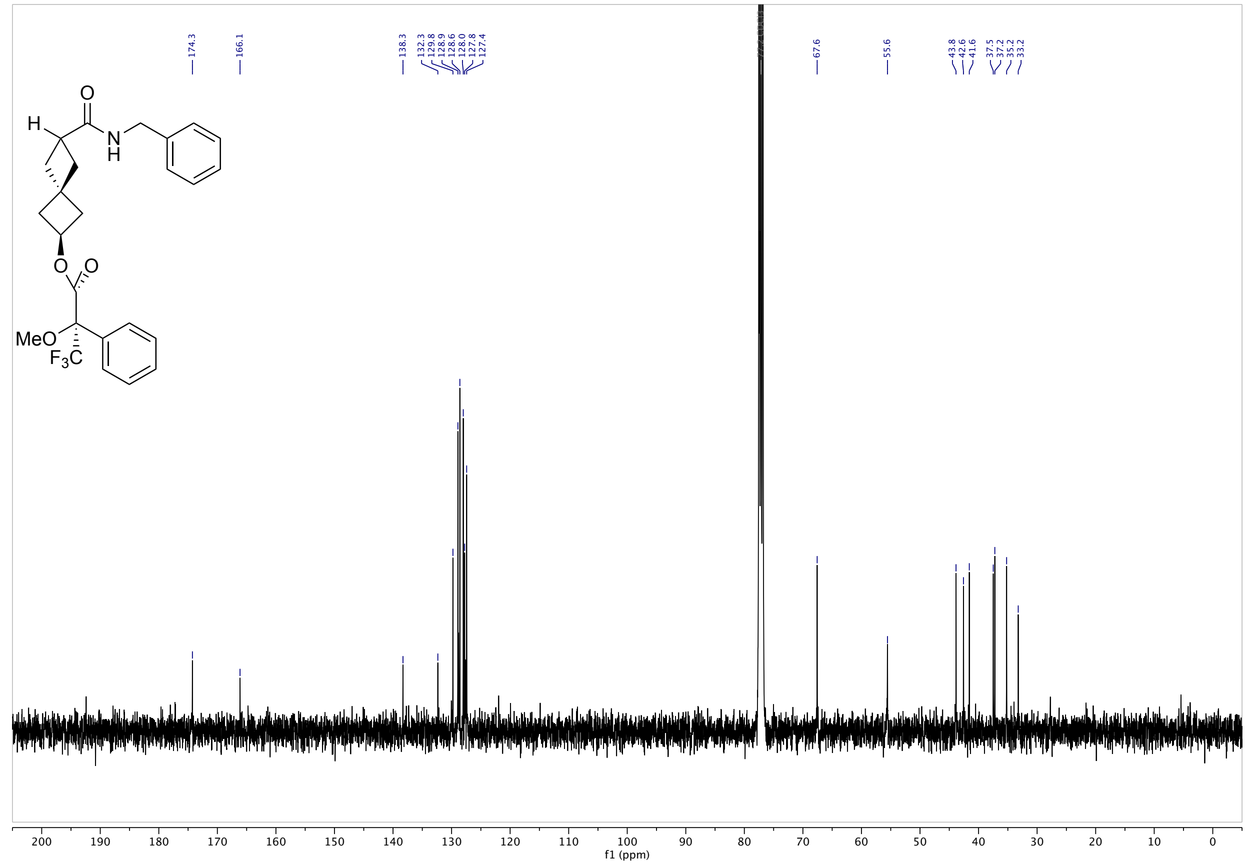

^{1}H NMR (400 MHz, $CDCl_3$) (S5)

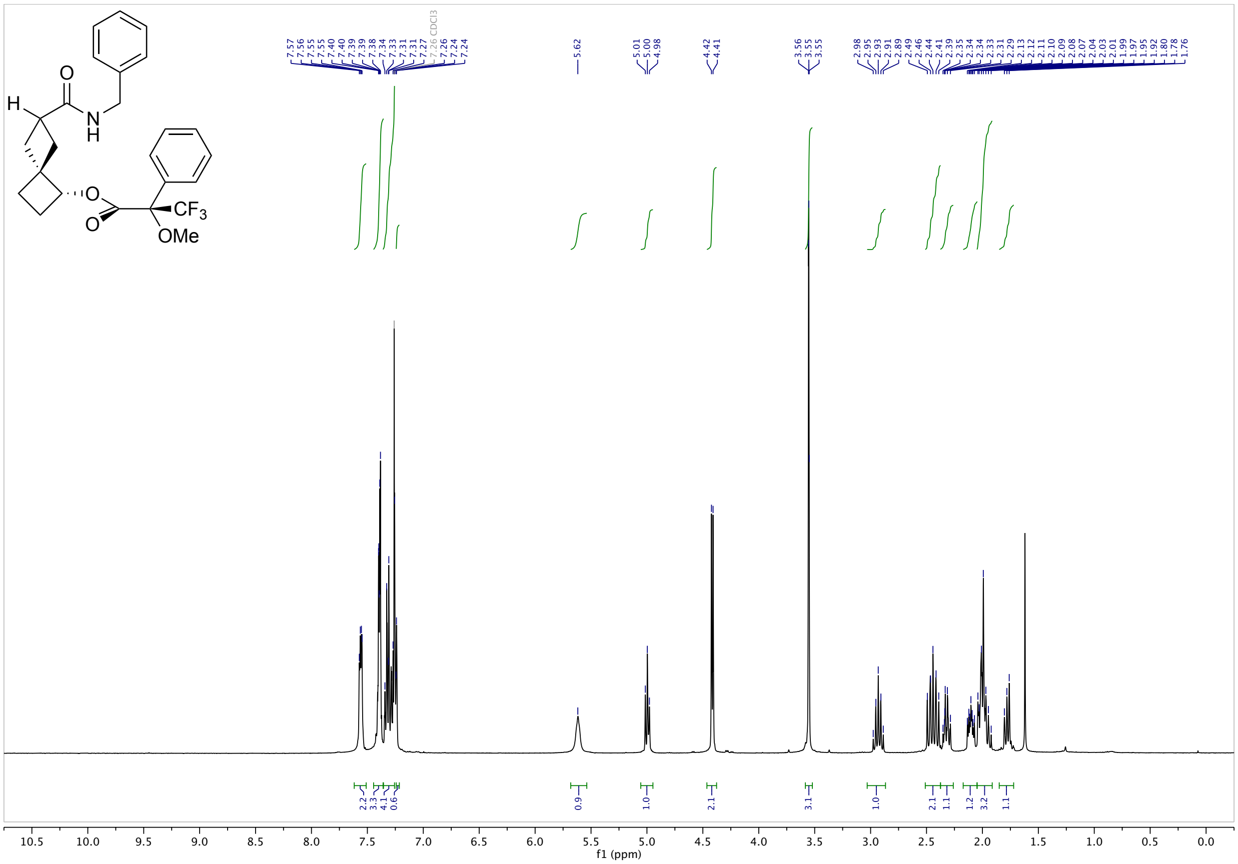

^{13}C NMR (101 MHz, $CDCl_3$) (S5)

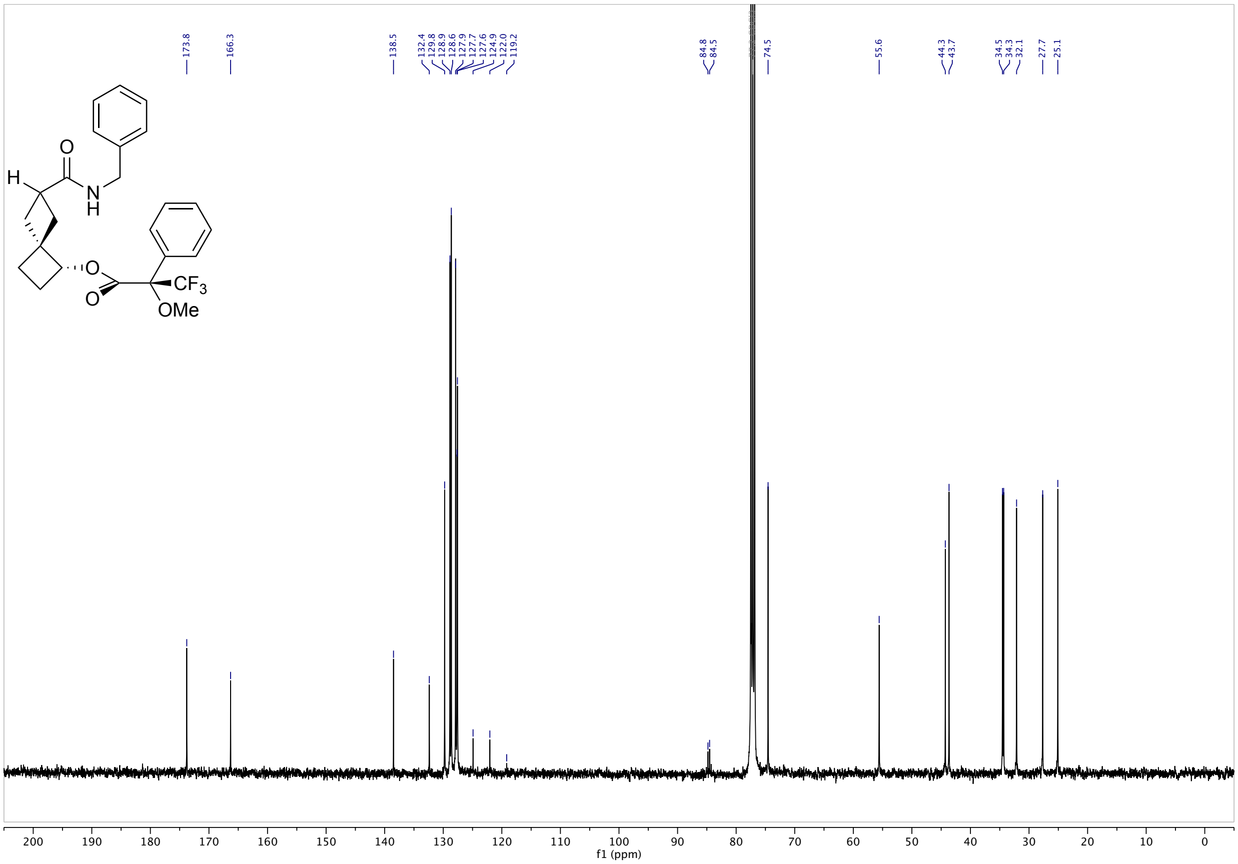

^{1}H NMR (400 MHz, $CDCl_3$) (S6)

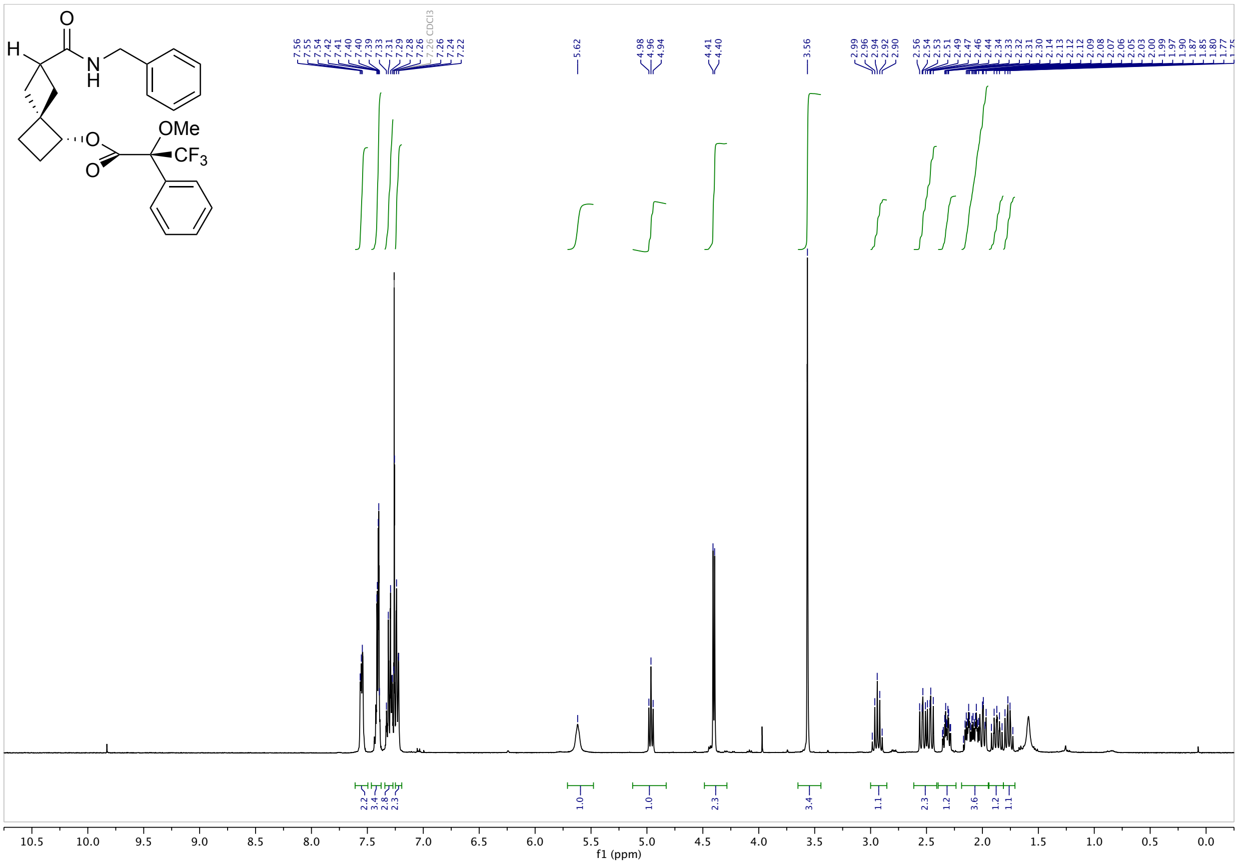

^{13}C NMR (101 MHz, $CDCl_3$) (S6)

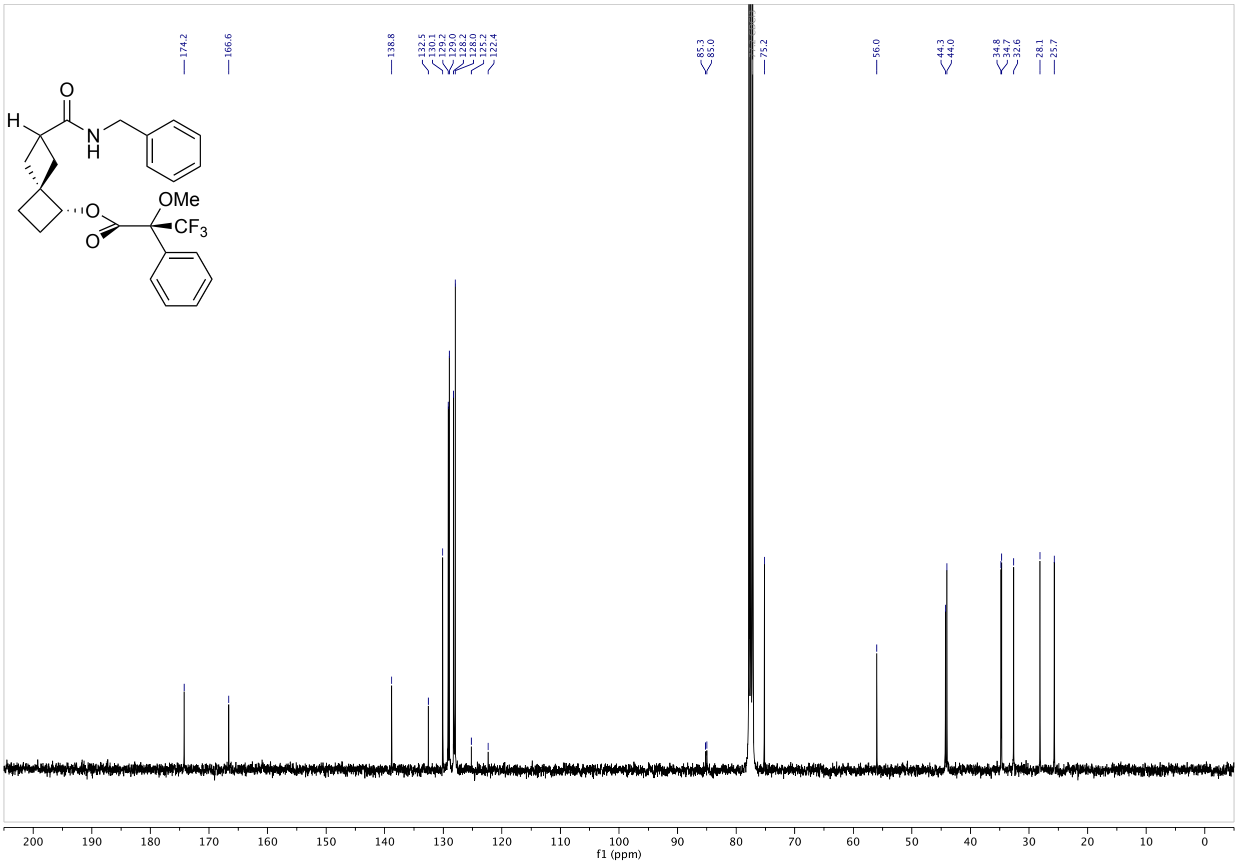

Supplement: Supplementary file 1 [file ol5c01265_si_001.pdf]
